# Study the Mechanism of Gualou Niubang Decoction in Treating Plasma Cell Mastitis Based on Network Pharmacology and Molecular Docking

**DOI:** 10.1155/2022/5780936

**Published:** 2022-06-15

**Authors:** Zhaojing Wu, Qing Yang, Hongbo Ma

**Affiliations:** ^1^First College of Clinical Medicine, Shandong University of Traditional Chinese Medicine, Jinan, Shandong 250355, China; ^2^Shandong Provincial Hospital Affiliated to Shandong First Medical University, Jinan, Shandong 250021, China

## Abstract

**Objective:**

Explore the potential molecular mechanisms behind the therapeutic functions of Gualou Niubang decoction (GLNBD) in the treatment of plasma cell mastitis (PCM) by network pharmacology and molecular docking.

**Methods:**

GLNBD is a formula of Chinese traditional medicine consisting of 12 herbs. The potential active ingredients of GLNBD and their target genes were obtained from the Traditional Chinese Medicine Systems Pharmacology Database and Analysis Platform database, and PCM-related target genes were obtained from GeneCards, OMIM, and NCBI databases, using R language to obtain intersection targets; then, the STRING database and Cytoscape software were used to establish protein-protein interaction networks and herb ingredient target networks. DAVID was used to perform GO and KEGG pathway enrichment analyses on the intersection target. PyMoL-2.5.0 and AutoDock Tools-1.5.6 were used to verify the molecular docking.

**Results:**

164 ingredients and 58 intersection targets were obtained in the treatment of PCM by GLNBD. Four key active compounds and four key proteins were identified. Then, Gene Ontology and Kyoto Encyclopedia of Genes and Genomes enrichment analyses showed that biological functions of potential target genes were associated with negative regulation of the apoptotic process, response to hypoxia, positive regulation of transcription, and DNA-templated, with related pathways involving the pathway in cancer, phosphatidylinositol 3-kinase (PI3K) Akt signaling pathway, and AGE-RAGE signaling pathway in diabetic complications. Moreover, the binding activities of key target genes and essential active compounds of Chinese herbal medicines in GLNBD were further validated by molecular docking. The results showed that the docking results were stable and had good binding ability.

**Conclusion:**

This study suggested that four potential key active components, including quercetin, luteolin, fisetin, and kaempferol, were identified in GLNBD, which could interact with ALB, EGFR, IL-6, and VEGFA modulating the activation of the pathway in cancer, PI3K-Akt pathway, and AGE-RAGE signaling pathway in diabetic complications.

## 1. Introduction

Plasma cell mastitis (PCM), also known as mammary gland duct dilation or occlusive mastitis, is a kind of chronic nonbacterial mammary gland inflammation. The incidence of PCM is mainly in nonpregnant and nonbreastfeeding women and occasionally occurs in males; the incidence rate of breast benign diseases is 4.1%~5.5 [1, 2]. The incidence rate of the disease has been increasing in recent years [3]. Its pathological basis is breast duct dilation, plasma cell infiltration, granuloma formation, etc. Clinical manifestations include irregular breast pain, nipple discharge (sometimes accompanied by the affected side of the axillary lymph node enlargement), areolar mass or subareolar nodules, skin redness, swelling and itching, nipple retraction, and areolar fistula. Because the clinical manifestations are not specific and the imaging manifestations are very similar to breast cancer, it is easy to misdiagnose and mistreat [4, 5]. At present, there is a lack of specific drugs in clinical treatment, and surgical treatment is mainly used. However, surgical treatment has a long course of disease and easy to relapse, postoperative scar affects the appearance, and the implementation of mastectomy will also bring mental and psychological harm to patients [6, 7]. PCM belongs to the category of “acne mammary carbuncle” of TCM [8]. Based on the theory of TCM, my tutor believes that the pathogenesis of this disease is mainly liver qi stagnation and heat toxin obstruction; the treatment should focus on soothing liver qi stagnation, clearing heat, removing swelling and lump and drainage of pus, and dissolving carbuncle which have a good clinical effect [9]; however, the underlying pharmacological mechanism of GLNBD against PCM is still illusive.

Gualou Niubang decoction (GLNBD) is derived from “Yizong Jinjian,” which is used to treat breast cellulitis and acute mastitis. The original formula is composed of Gua Louren (Fructus Trichosanthis), Niu Bangzi (Fructus arctii), Tian Huafen (Trichosanthin), Huangqin (Baikal skullcap), Zhizi (Gardenia), Lianqiao (Forsythia suspensa Vahl), Zaoci (Gleditsia sinensis), Jin Yinhua (honeysuckle), Gancao (licorice), Chenpi (dried tangerine peel), Qingpi (Pericarpium Citri Reticulatae Viride), and Chaihu (Radix bupleuri). Modern pharmacological research shows that Niu Bangzi has antibacterial, antiviral, antitumor, and other functions [10]. Huangqin can fight inflammation and allergic reaction, thus improving abscess [11]. Jin Yinhua, Zhizi, and Lianqiao all have anti-inflammatory effects [12]. Chaihu can relieve fever and is analgesic, anti-inflammatory, antibacterial, and antidepressant, which can reduce the pain caused by inflammation and facilitate wound recovery [13]. In this paper, network pharmacology and molecular docking technology will be used to predict the target of GLNBD in the treatment of PCM and then speculate the mechanism of its action in treating the disease, providing new ideas for follow-up clinical research and treatment.

**Figure 1:**
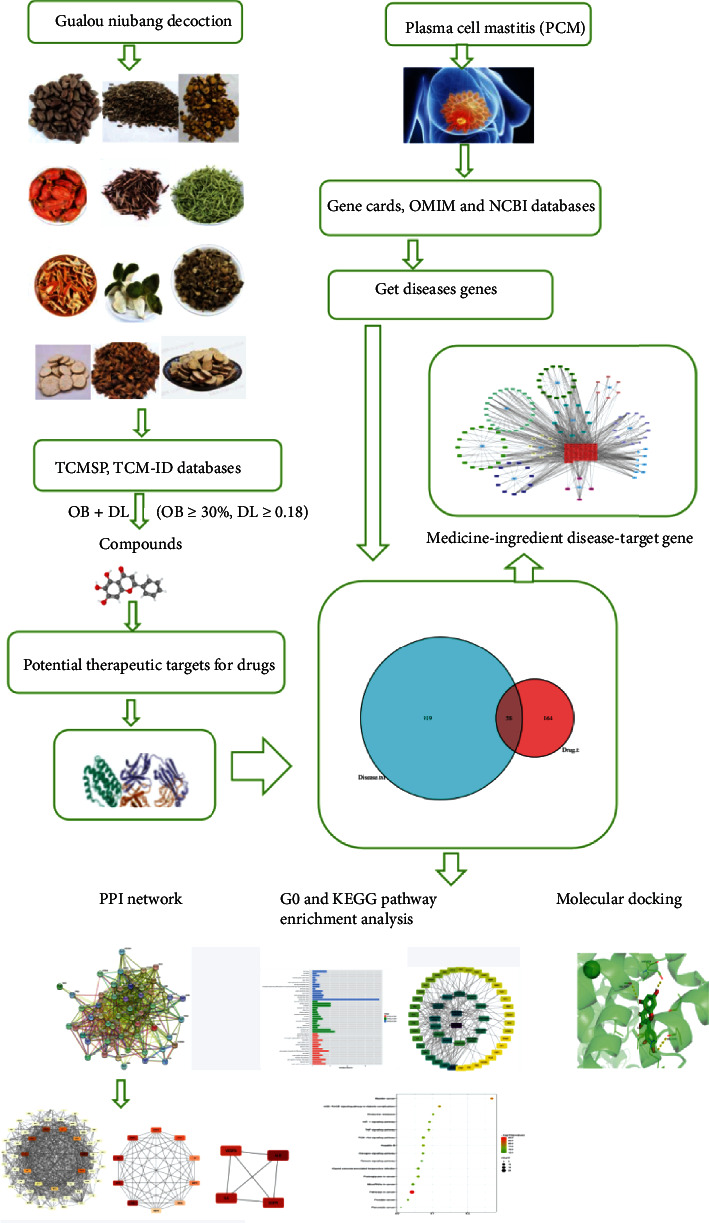
Framework based on an integration strategy of network pharmacology.

Network pharmacology was first proposed by British pharmacologist Andrew T·Hopkins in 2007 and described as a next-generation drug development model in 2008 [14], which is a new method that can determine how TCM works through pharmacokinetic evaluation, to study its molecular mechanism network pharmacology which can analyze the mechanism of action of multicomponent and multitarget TCM compounds and transform the single-target and single-drug model into network targets; the multicomponent treatment mode finally better reveals the pharmacodynamic material basis and molecular mechanism of TCM, to more effectively promote the in-depth research of TCM compounds [15]. Molecular docking is a theoretical simulation method to reveal the interaction between receptors and drug molecules and predict their affinity. In this study, we hope to investigate the possible molecular mechanism of GLNBD in the treatment of PCM through network pharmacology and molecular docking, providing a new method for clinical treatment. An outline of the method is shown in Figure 1.

## 2. Materials and Method

### 2.1. Collection of Main Components and Disease Targets of Gualou Niubang Decoction

All chemical ingredients and their related targets of each herb of GLNBD were collected from the Traditional Chinese Medicine Systems Pharmacology (TCMSP) (https://old.tcmspe.com/tcmsp.ph) [16] and TCM-ID (http://bidd.group/TCMID/) database [17]. Ingredients were screened conditional on ADME (“ADME” refers to the progress of absorption, distribution, metabolism, and excretion of exogenous chemicals by the body); the qualified active compounds and their targets were screened according to the two ADME attribute values of oral bioavailability (OB) ≥ 30% and drug likeness (DL) ≥ 0.18. The corresponding molecular structure, structural information, and PubChem CID of those active ingredients were further obtained from the “PubChem” online tool (https://pubchem.ncbi.nlm.nih.gov/) [18].

### 2.2. Construction of the PCM-Related Target Database

“Plasma cell mastitis”, “mastitis obliterans”, and “mammary duct ectasia” were searched as the search terms in GeneCards (https://www.genecards.org/) [19], NCBI (https://www.ncbi.NLM.Nih.gov/) [20], and OMIM (https://www.omim.org/) database [21], which were used to search for disease targets; retrieval results were combined to delete repeated targets and obtain PCM-related targets.

### 2.3. Obtain Intersection Target Genes and Draw a Venn Diagram

Validated human species-associated target genes were selected in the Uniprot database [22], and gene names were obtained with R language Perl scripts. The drug-related genes were intersected with disease-related genes with a Perl script, and an intersection Venn diagram was drawn. Finally, potential targets of PCM for GLNBD treatment were obtained.

### 2.4. Build Protein-Protein Interaction Network

The intersection target genes were imported into the STRING database (https://string-db.Org/) [23], and a protein interaction relationship file was obtained and imported into Cytoscape [24]. To map the PPI network of drugs for the treatment of PCM by network analysis, according to the degree obtained, the network of the top 4 genes was plotted.

### 2.5. Construction of a Prescription-Ingredient-Target-Disease Network for Gualou Niubang Decoction

The network of the visualization of GLNBD was generated by importing the two files above into Cytoscape 3.7.2. The top four active ingredients were selected as key ingredients based on the degree. Degree indicated the number of edges between a single node and other nodes in a network.

### 2.6. Gene Ontology and Kyoto Encyclopedia of Genes and Genomes Enrichment Analyses of Intersection Target Network

DAVID was used to analyze the GO biological process and KEGG signal pathway enrichment of intersection target proteins and obtain GO and KEGG signal pathways of GLNBD in the treatment of PCM.

### 2.7. Molecular Docking of Key Ingredients to Key Targets

The top four key active compound mol2 format files were downloaded from the ZINC database, and small molecules were extracted using Python. After removing the redundant water molecules from the protein crystal structure and adding hydrogen atoms, we batch processed them into pdbqt files with Openbable to complete ligand preparation. Receptor files were prepared by downloading pdb format files of four key targets from the Protein Data Bank (PDB) (http://www.pdb.org/) database [25] and converting them to pdbqt files using AutoDock Tools. The docking pocket is a possible binding site for ligands in the receptor. We set up a docking box large enough to cover the entire receiver so that it contains all possible docking pockets. The size of the docking box varies depending on different receptors. Bulk molecular docking was done with AutoDock Tools to predict the binding activity of the protein to the ingredient. The best combination of protein and component is the receptor and ligand with the minimum affinity. It is generally believed that the smaller the affinity of the receptor to the ligand and the stronger its stability, the better will be the docking. The active compound is molecular docking of the hypothetical target, and its free binding energy is measured. The interaction and binding modes of active compounds were visually analyzed by PyMOL [26].

## 3. Results

### 3.1. Predictive Results of Targets for Gualou Niubang Decoction in Plasma Cell Mastitis

A total of eight ingredients of Niu Bangzi, 2 ingredients of Tian Huafen, 15 ingredients of Zhizi, 11 ingredients of Zaoci, 5 ingredients of Qingpi, 17 ingredients of Chaihu, 36 ingredients of Huangqin, 23 ingredients of Jin Yinhua, 23 ingredients of Lianqiao, 5 ingredients of Chenpi, and 92 ingredients of Gancao were collected from the TCMSP database according to OB ≥ 30% and DL ≥ 0.18. Find the 3 active ingredients of GLR from TCM-ID. After deduplication and deletion of PubChem CID, 164 drug active components were obtained (Table 1, Tables S1 and S2).

### 3.2. Target Collection of PCM

977 disease-related targets were obtained from the GeneCards, OMIM, and NCBI databases; the target information was standardized for gene names.

### 3.3. Screening of Intersection Targets

**Figure 2:**
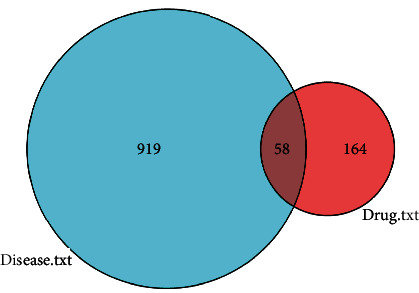
Venn diagram of drug component targets of GLNBD and PCM disease targets.

Take the intersection of the target gene results obtained from Section 3.1 and 3.2 screening to obtain 58 key intersection genes and draw the Venn diagram (Figure 2, Table S3).

### 3.4. Establishment of PPI Network

**Figure 3:**
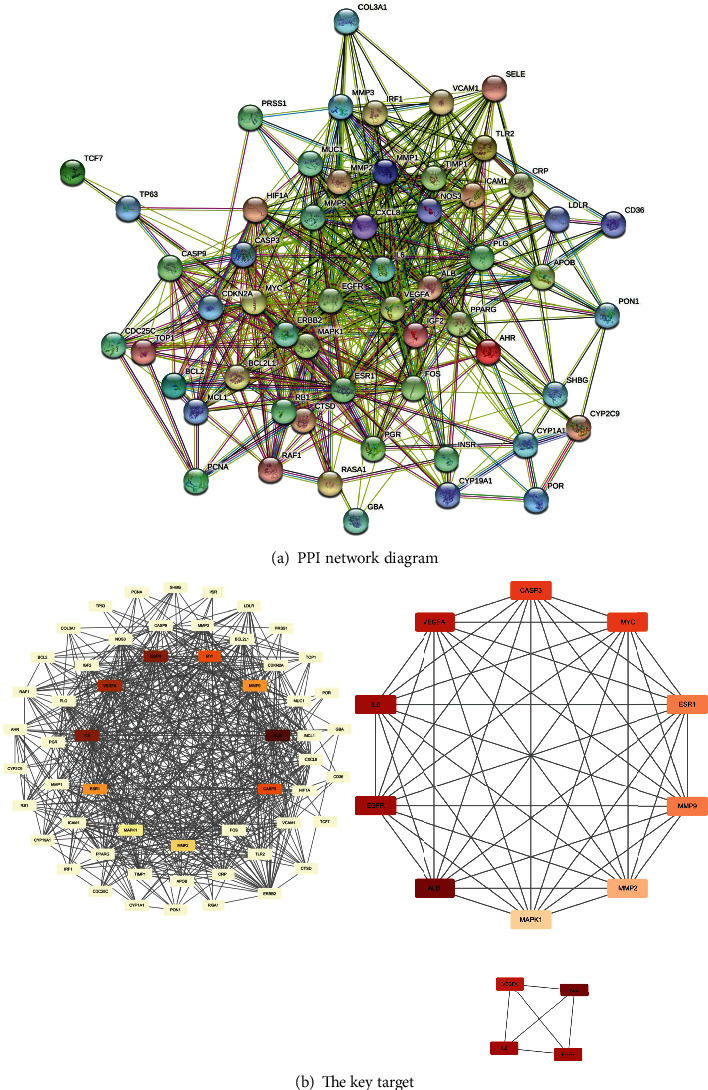
PPI network of common drug-disease targets.

Based on the STRING database, the protein interaction network of intersection targets was constructed (Figure 3(a)). The network contained 58 nodes and 591 edges, TSV files were saved, and a total of 29 nodes with a degree value greater than the average value 20.4 were statistically sorted according to the degree value of intermediate proximity (Table 2, Figure 3(b)). To further screen key proteins, TSV files were imported into Cytoscape, and the network was analyzed by the cytoNCA plug-in. The screening conditions were arranged according to degree value. The darkest targets are albumin (ALB), interleukin-6 (IL-6), epidermal growth factor receptor (EGFR), and vascular endothelial growth factor A (VEGFA). Then, there are Caspase-3 (CASP3), protooncogene (MYC), estrogen receptor 1 (ESR1), matrix metalloproteinase 9 (MMP9), matrix metalloproteinase 2 (MMP2), and mitogen-activated protein kinase 1 (MAPK1).

### 3.5. Network Construction

**Figure 4:**
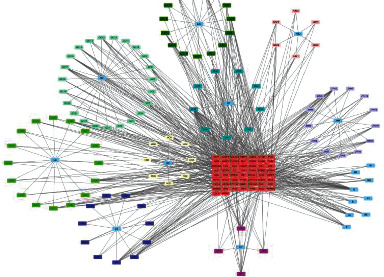
Red is the disease target, drug target (blue; Zhizi (ZZ); pink: Chenpi (CP), yellow: uangqin (HQ); green: LIanqiao (LQ); nattier blue: Gancao (GC); bollie green: Zao jiaoci (ZJC); light red: Niu bangzi (NBZ); purple: Jian yinhua (JYH); cyan: Chaihu (CH); the rest are common components).

Based on Cytoscape 3.7.2, a complete network of active component-ingredient-disease-target gene was constructed (Figure 4, Table S4), with 110 compound nodes and 58 protein nodes, and the average degree value was 10.6, a chemical component often corresponds to multiple intersecting gene targets, and an intersecting gene and target also correspond to multiple chemical components simultaneously, which indicates that GLNBD is effective in treating PCM through multiple components, multiple targets, and multiple pathways; among them, quercetin, luteolin, fisetin, and kaempferol were the main active ingredients with a degree value higher than or equal to the degree value (Table 3).

### 3.6. Results of Gene Ontology and Kyoto Encyclopedia of Genes and Genomes Enrichment Analyses

**Figure 5:**
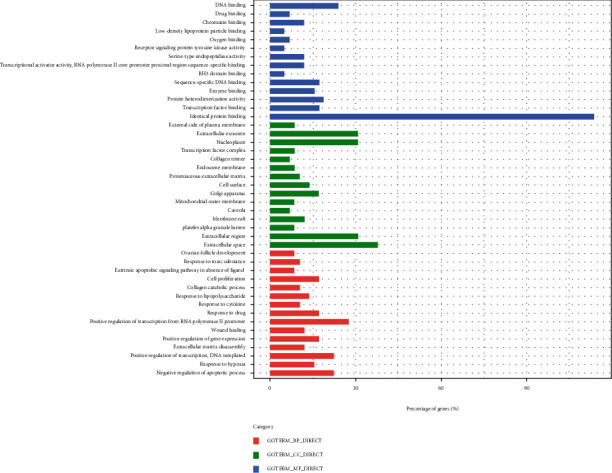
Bioanalysis of G0 potential target of GLNBD in the treatment of PCM.

In this study, GO enrichment analysis provided a more systematic perspective for elucidating the mechanisms of GLNBD in the treatment of PCM (Figure 5, Tables S5–S7). The GO program is a primary biological information method for expressing genes encoded in biological functions at the level of tissue systems, cells, and molecules. The results of cellular component (CC) showed that most of the targets were enriched in extracellular space, extracellular region, platelet alpha granule lumen, membrane raft, and axons with the molecular function (MF) of nerve impulse transmission. In terms of MF, these targets were obviously enriched in protein binding, identical protein binding, transcription factor binding, and protein heterodimerization activity. These findings implicated that various biological processes (BP) such as negative regulation of apoptotic process, response to hypoxia, positive regulation of transcription, DNA-templated, and extracellular matrix disassembly were involved in the multiple synergistic effects of GLNBD in the treatment of PCM.

**Figure 6:**
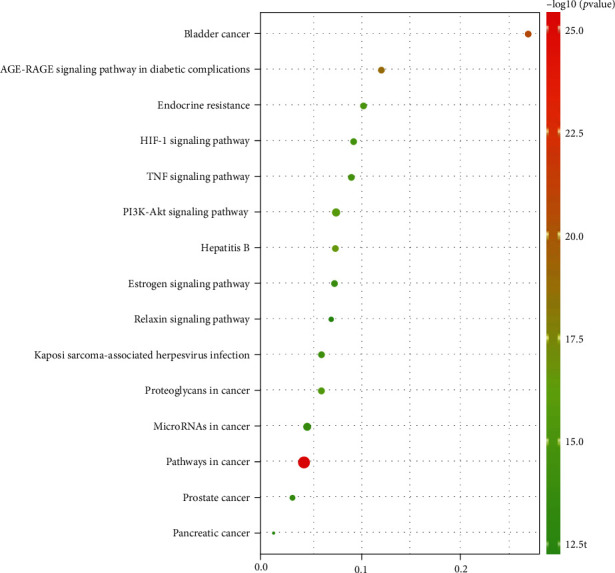
Enrichment of KEGG of potential target of GLNBD in treatment of PCM (Kyoto Encyclopedia of Genes and Genomes (KEGG) pathway enrichment. The *x*-axis represents the multiple of enrichment; the *y*-axis represents the name of the pathway; the color of the circles in the figure from green to red indicates that the *P* value increases from small to large; and circle sizes from small to large represent the number of genes from fewer to more. The circle size represents the count).

**Figure 7:**
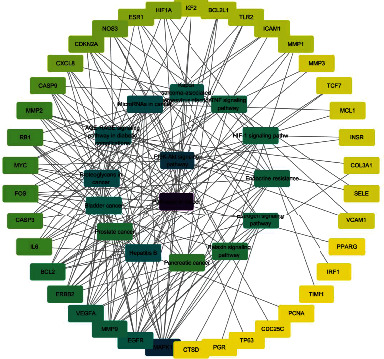
The top 15 signaling pathways and enrichment genes (the inner two rings are the signal pathway, the outermost ring is the gene enriched in the pathway, and the darker the color, the more obvious the enrichment).

To systematically explore the potential mechanisms of GLNBD active components, DAVID was used to extract all the pathways that interact with target proteins from the KEGG pathway database, producing 200 pathways. Most of the enriched top 15 pathways were not reported to be associated with PCM, such as pathways in cancer, bladder cancer, hepatitis B, proteoglycans in cancer, and bubble map visualization (Figure 6). Finally, three main pathways were involved in the treatment of PCM by GLNBD: PI3K/Akt signal pathway, HIF-1 signal pathway, TNF signal pathway, estrogen signal pathway, and AGE-RAGE signaling pathway in diabetic complications. The top ten generate a visual bubble map (Figure 6) and the relationship between the top 15 signaling pathways and protein genes (Figure 7, Table 4, and Table S8).

### 3.7. Molecular Docking

**Figure 8:**
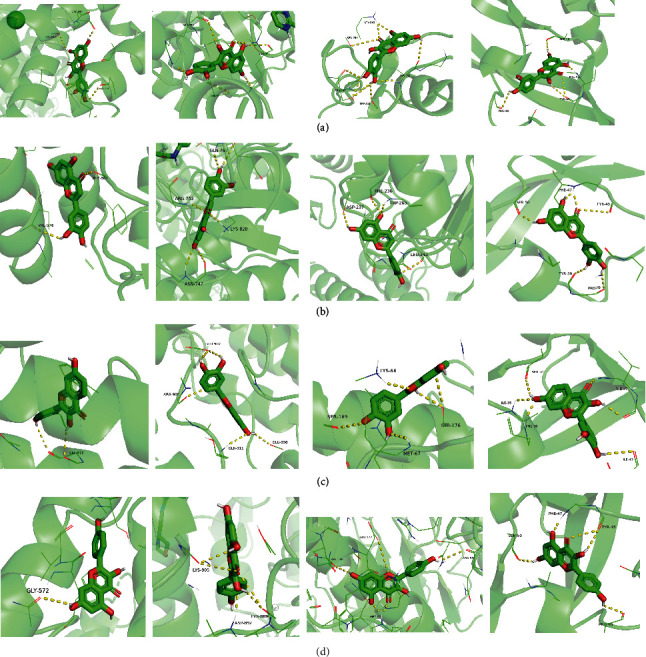
The protein-ligand of the docking simulation: (a) molecular docking results of quercetin with ALB, EFGR, IL-6, and VEGFA; (b) molecular docking results of luteinol with ALB, EFGR, IL-6, and VEGFA; (c) the molecular docking results of fisetin with ALB, EFGR, IL-6, and VEGFA; (d) molecular docking results of kaempferol with ALB, EFGR, IL-6 ,and VEGFA.

With the compound components, AutoDock Tools 1.5.6 was used to conduct molecular docking between the potential targets of GLNBD acting on PCM and the main compounds (quercetin, luteolin, kaempferol, and fisetin) determined by topology analysis (Figure 8, Table 5). The higher absolute value of a docking result indicates a stronger binding force between the active site of the protein receptor and the compound. The more stable the conformation of the ligand binding to the receptor is, the more likely it is to act. Docking scores are shown in Table 5 and Figure 8. Kaempferol which may be the main bioactive compound of GLNBD had the largest number of target points.

## 4. Discussion

In this study, the main active components of 12 traditional Chinese medicines of GLNBD were obtained according to TCMSP and TCM-ID database, ingredient screening, and target prediction; 164 ingredients of GLNBD were collected, with 222 drug corresponding treatment targets and 997 PCM disease-related targets. After intersection, 58 target genes were obtained, and then, GO and KEGG pathways were further enriched and analyzed.

### 4.1. Analysis of Active Components

The active components of quercetin, luteolin, fisetin, and kaempferol were predicted by network visualization. Quercetin is a dietary flavonoid with anti-inflammatory, antioxidant, and antifungal properties and has certain effects on inflammation, angiogenesis, and vascular inflammation. Experiments have confirmed that quercetin can enhance the transcriptional activity of NRF2-ARE by inhibiting the NF-*κ*B signaling pathway, thus playing a role in controlling bovine mastitis [27]. Quercetin has also been shown to play an anti-inflammatory role by inhibiting the NF-*κ*B and MAPK signaling pathways mediated by TLR4-MyD88 [28]. The anti-inflammatory mechanism of quercetin-nitric oxide (NO) is stimulated by inflammatory cytokines in macrophages and other cells to induce NO in inflammatory conditions; studies have shown that quercetin can reduce the expression of inducible nitric oxide synthase (iNOS) and regulate the production of inflammatory precursor NO. [29] Luteolin has significant systemic anti-inflammatory effects. It can regulate the TLR2 and TLR4 signaling pathways induced by Staphylococcus aureus, inhibit the phosphorylation of I*κ*B*α* and NF-*κ*B P65, regulate the expression of MMP2 and MMP9, and prevent mastitis [30]. Luteolin can reduce TNF-*α* by enhancing the expression of arginase (ARG-1) IL-10. The expression of IL-6 iNOS can also change the polarization of macrophages, thus playing an anti-inflammatory role [31]. Luteolin has been proved to have anti-inflammatory activity against ulcerative colitis in vivo and intestinal tract in vitro [32, 33]. Experiments have confirmed that luteolin can be used as a systemic and effective anti-inflammatory agent by regulating inflammatory mediators [34]. Fisetin is widely used in regulating long-term immune dysregulation of inflammation in the human body [35]. Fisetin can activate SIRT1 and inhibit the activation of the NF-*κ*B inflammatory pathway, thereby reducing the expression level of TNF-*α* and IL-6 and controlling the occurrence of inflammation [36]. In vivo study of laccflavin for nephritis, endometritis, and airway inflammation and in vitro study of inflammatory skin disease have a good anti-inflammatory effect [37–40]. Kaempferol can reduce the expression of IL-6 and TNF-*α* and ANGPTL2 in cells, prevent the occurrence of mastitis in mice, inhibit the phosphorylation of NF-*κ*B P65 subunit and the degradation of I*κ*B*α*, and play a therapeutic role in mastitis [41].

### 4.2. Analysis of Core Targets

PPI network analysis showed that the key genes were ALB, IL-6, EGFR, and VEGFA. ALB has been mentioned in many inflammatory studies, and the expression of the ALB gene can also be detected in peripheral blood monocytes. For example, ALB is lower than normal in patients with lymph node tuberculosis, and neutrophil ratio and red blood cell distribution width are closely related to ALB in patients with systemic lupus erythematosus (SLE); the expression of the ALB gene can reflect the function of differentiated liver cells in patients with hepatitis [42, 43]. In addition, studies have confirmed that activation of ALB expression can reduce the inflammatory damage of mastitis and inhibit the release of inflammatory cytokines [44]. IL-6 secretion is involved in the immune regulation of the body's inflammatory response, which can promote the development of helper T cells, inhibit the induction of regulatory T cells, and promote the self-reactive proinflammatory cell response [45]. It can also promote the differentiation of B lymphocytes into plasma cells by activating the IL-6/JAK2/STAT3 pathway in B lymphocytes. Some researchers successfully completed modeling of PCM mice by injecting IL-6 into mouse mammary tissue [46, 47]. Some scholars also conducted clinical observation studies and found that serum IL-6 increased significantly at the onset of PCM but decreased significantly after treatment [48]. EGFR is a member of the epidermal growth factor receptor (HER) family. EGFR dimerization activates its intracellular kinase pathway and regulates phosphorylation of downstream signaling pathways, including MAPK, Akt, and JNK pathways, which can not only play its immune function but also cause the proliferation of mammary epithelial cells and increase the expression of inflammatory repair factors. It is beneficial for tissue repair [49, 50]. VEGFA at present is considered to be the strongest vascular permeability factor; the immune response, inflammation, mastitis, tumor, ischemia, and hypoxia play a key role in many physiological and pathological cell infiltration, stimulate the production of endothelial cell matrix degradation of protease, cause hemal wall integrity being impaired, and increase permeability leading to the vascal process [53]. Research shows that VEGFA can cause mammary gland tissue inflammation injury, breast swelling, and increased inflammation; inhibition of VEGFA release can reduce the inflammatory response, which has therapeutic effects to a certain extent [51].

### 4.3. Biological Function and Pathway Enrichment Analysis

According to GO function enrichment analysis, BP is negative regulation of apoptotic process, response to hypoxia, positive regulation of transcription, DNA-templated, extracellular matrix disassembly, positive regulation of gene expression, and wound healing. MF is protein binding, identical protein binding, protein heterodimerization activity, enzyme binding, sequence-specific DNA binding, and BH3 domain binding. It can be seen that the treatment of PCM with GLNBD is related to the binding of protein; the inflammatory process is related to a variety of binding proteins, such as the binding protein is related to the airway inflammatory response of children's asthma [52]. Binding protein plays an important role in the treatment of sepsis and septic shock [53]/ Plasma fibronectin plays an important role in the pathogenesis of severe pneumonia in the elderly [54]. CC is extracellular space, extracellular region, platelet alpha granule lumen, caveola, and mitochondrial outer membrane GLNBD mainly playing an anti-inflammatory role in the extracellular area. In addition, membrane raft plays an important role in angiogenesis and the treatment of inflammatory diseases and affects the transmission of microbial pathogens and inflammation induction, which provides a new method for antibacteria and anti-inflammation [55].

The enrichment analysis of the KEGG pathway showed that the possible signal pathways for the treatment of PCM by GLNBD were the AGE-RAGE signaling pathway in diabetic complications and PI3K/Akt signal pathway. Studies have confirmed that after activation of the AGE-RAGE signaling pathway, a large amount of VEGF will be secreted and released to promote the generation of new blood vessels and contribute to the repair of PCM tissue. It also causes the expression and release of a large number of proinflammatory cytokines, such as IL-6 and TNF-*α*, and causes the destruction of tissue cells [37, 54]. AGE and RAGE can also activate downstream signal transduction pathways, such as the PI3K/Akt pathway. The PI3K/Akt signaling pathway plays an important role in metabolism, growth, proliferation, survival, transcription, and protein synthesis. It inhibits the PI3K/Akt signaling pathway and regulates tight junction protein expression to protect the integrity of the blood-milk barrier and reduces the incidence rate of mastitis in mice [56]. Akt activation is closely related to inflammatory response. Inhibiting Akt phosphorylation can prevent NF-*κ*B nuclear translocation, while inflammatory factors such as TNF-*α* and IL-6 were also inhibited [57]; therefore, the inflammatory response will also be controlled, which shows that the inflammation caused by proinflammatory factors can be controlled by inhibiting the pathway [58]. PCM tissues had higher levels of p-Akt than normal tissues. This suggests that the activated Akt signaling pathway may play an important role in PCM [59]. The activation of the PI3K/Akt signaling pathway can be mediated by JAK, and inflammatory factors may upregulate the PI3K-Akt signaling pathway by activating the JAK2/STAT3 signaling pathway [56]. RNA-SEQ was used to analyze the difference in exosome transcriptome expression between PCM and normal breast tissue. It was found that p-Akt was produced too much in the process of PCM, and inhibiting exosome secretion could significantly inhibit inflammatory cell infiltration, so that the breast duct in the PCM mouse model could maintain a good structure [60]. Overall, the above pathways may be closely relevant to GLNBD treatment of PCM.

## 5. Conclusion

In this study, the interactions of 164 bioactive compounds and 58 potential targets for GLNBD treatment of PCM were identified through network pharmacological analysis. These targets are closely related to biological processes such as negative regulation of apoptosis, hypoxia response, and wound repair. The AGE-RAGE signaling pathway and PI3K/Akt signal pathway in diabetic complications are involved in these biological processes. The binding ability of quercetin, luteolin, fisetin, and kastricol to ALB, IL-6, EGFR, and VEGFA was verified by molecular docking, while kaempferol showed a strong affinity for targets. This study provides important clues for further exploring the mechanism of GLNBD in the treatment of PCM. However, in vivo or in vitro experiments are needed to verify the mechanism of GLNBD in treating PCM by regulating the abovementioned core targets and pathways.

## Figures and Tables

**Table 1:** Information and PubChem CID of active ingredients in Gualou Niubang decoction (excluding duplication).

Herbs	MOLname	Components	OB	DL	PubChem CID
Niu Bangzi	MOL010868	Neoarctin A	39.99	0.27	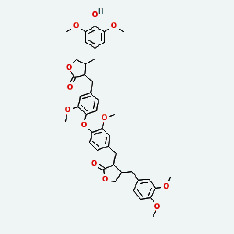
Niu Bangzi	MOL000522	Arctiin	34.45	0.84	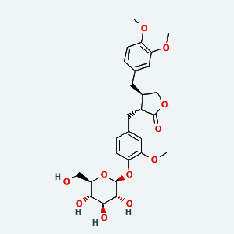
Niu Bangzi	MOL000358	Beta-sitosterol	36.91	0.75	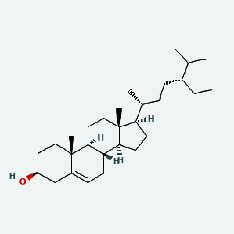
Niu Bangzi	MOL000422	Kaempferol	41.88	0.24	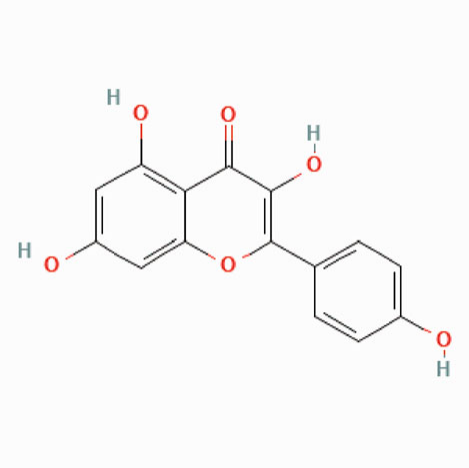
Niu Bangzi	MOL001506	Supraene	33.55	0.42	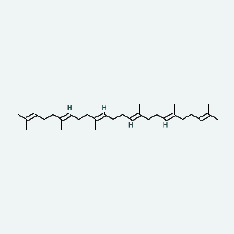
Niu Bangzi	MOL002773	Beta-carotene	37.18	0.58	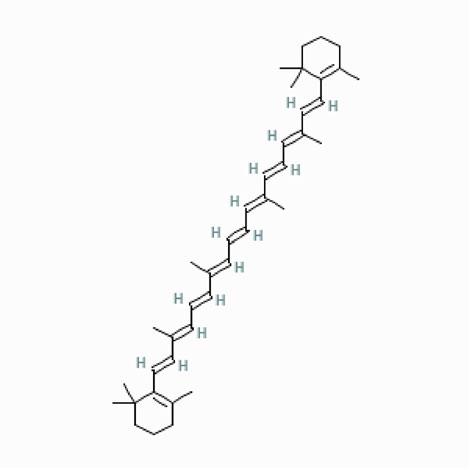
Niu Bangzi	MOL003290	(3R,4R)-3,4-Bis[(3,4-dimethoxyphenyl)methyl]oxolan-2-one	52.3	0.48	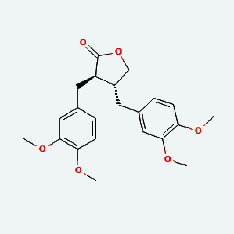
Niu Bangzi	MOL007326	Cynarin(e)	31.76	0.68	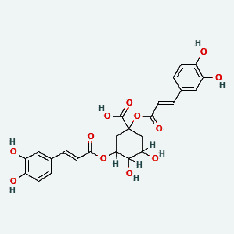
Tian Huafen	MOL004355	Spinasterol	42.98	0.76	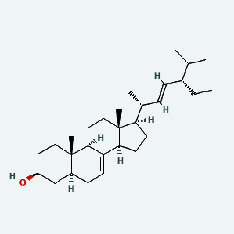
Tian Huafen	MOL006756	Schottenol	37.42	0.75	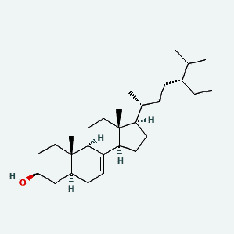
Zhizi	MOL001406	Crocetin	35.3	0.26	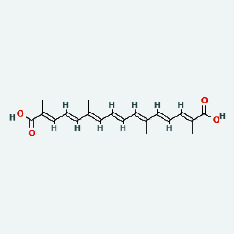
Zhizi	MOL004561	Sudan III	84.07	0.59	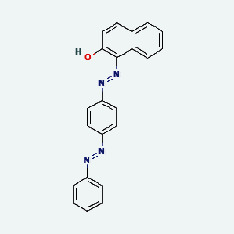
Zhizi	MOL000098	Quercetin	46.43	0.28	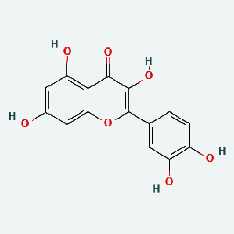
Zhizi	MOL000449	Stigmasterol	43.83	0.76	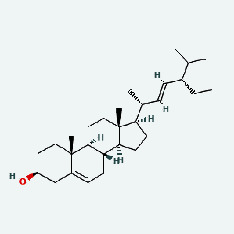
Zhizi	MOL001494	Mandenol	42	0.19	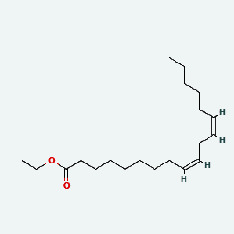
Zhizi	MOL001506	Supraene	33.55	0.42	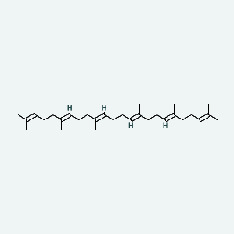
Zhizi	MOL001942	Isoimperatorin	45.46	0.23	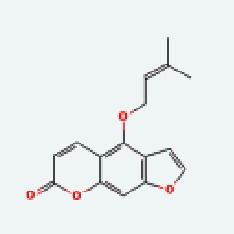
Zhizi	MOL002883	Ethyl oleate (NF)	32.4	0.19	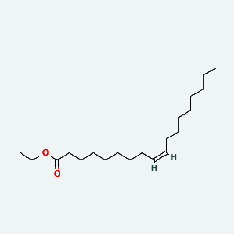
Zhizi	MOL007245	3-Methylkempferol	60.16	0.26	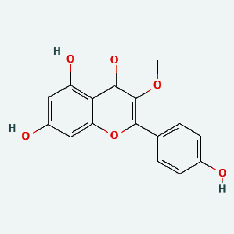
Zhizi	MOL009038	GBGB	45.58	0.83	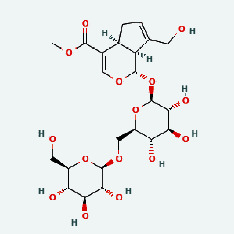
Zaoci	MOL013179	Fisetin	52.6	0.24	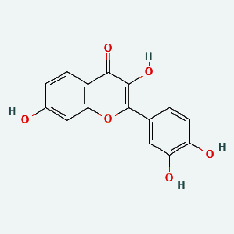
Zaoci	MOL013296	Fustin	50.91	0.24	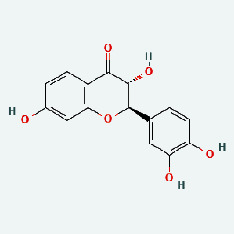
Zaoci	MOL001736	(-)-Taxifolin	60.51	0.27	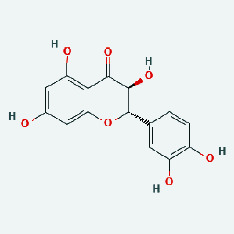
Zaoci	MOL002914	Eriodyctiol (flavanone)	41.35	0.24	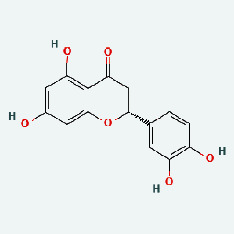
Zaoci	MOL000359	Sitosterol	36.91	0.75	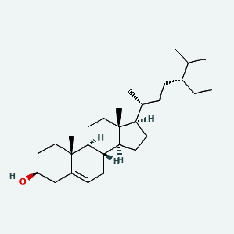
Zaoci	MOL006358	Stigmast-4-ene-3,6-dione	39.12	0.79	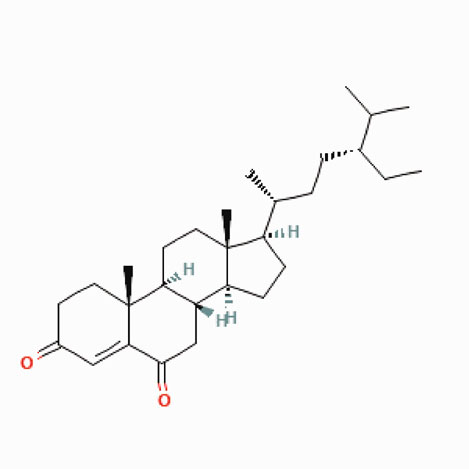
Zaoci	MOL000073	Ent-epicatechin	48.96	0.24	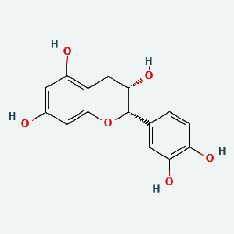
Qingpi	MOL001803	Sinensetin	50.56	0.45	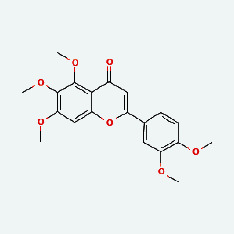
Qingpi	MOL005100	5,7-Dihydroxy-2-(3-hydroxy-4-methoxyphenyl)chroman-4-one	47.74	0.27	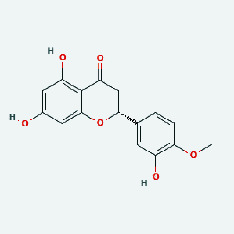
Qingpi	MOL010868	Neoarctin A	39.99	0.27	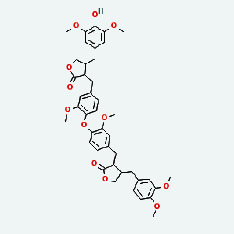
Qingpi	MOL000522	Arctiin	34.45	0.84	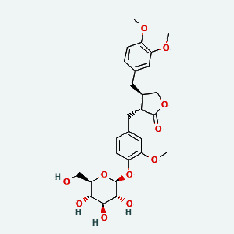
Chaihu	MOL000358	Beta-sitosterol	36.91	0.75	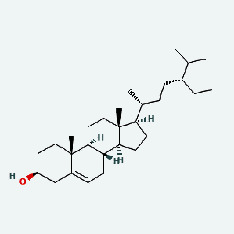
Chaihu	MOL000422	Kaempferol	41.88	0.24	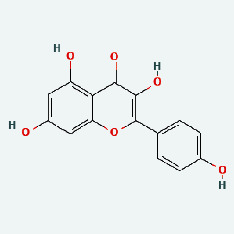
Chaihu	MOL001506	Supraene	33.55	0.42	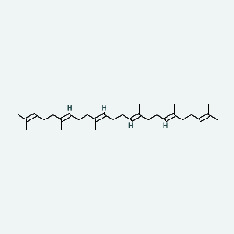
Chaihu	MOL002773	Beta-carotene	37.18	0.58	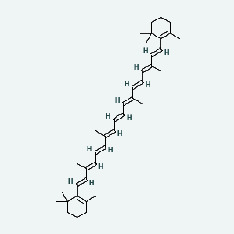
Chaihu	MOL003290	(3R,4R)-3,4-Bis[(3,4-dimethoxyphenyl)methyl]oxolan-2-one	52.3	0.48	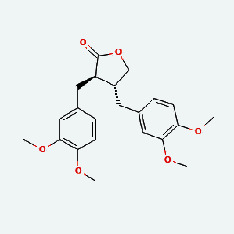
Chaihu	MOL007326	Cynarin(e)	31.76	0.68	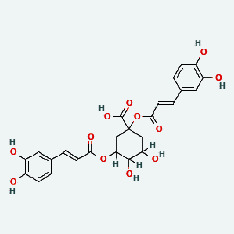
Chaihu	MOL004355	Spinasterol	42.98	0.76	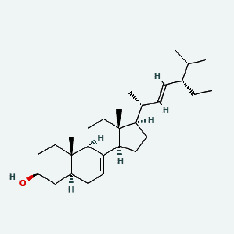
Chaihu	MOL006756	Schottenol	37.42	0.75	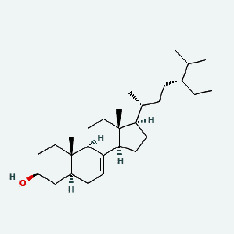
Chaihu	MOL001406	Crocetin	35.3	0.26	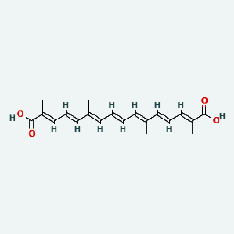
Chaihu	MOL004561	Sudan III	84.07	0.59	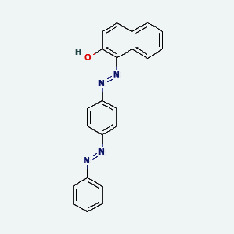
Chaihu	MOL000098	Quercetin	46.43	0.28	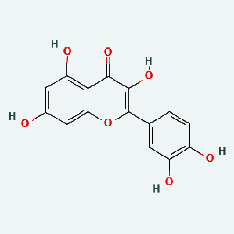

**Table 2:** List of key target proteins.

Target	Degree	Betweenness	Closeness
ALB	44	245.30997	0.8235294
IL6	42	171.66898	0.8
EGFR	42	172.46779	0.8
VEGFA	41	142.68523	0.7887324
CASP3	39	144.2639	0.7671233
MYC	39	169.84193	0.7671233
ESR1	36	108.049736	0.7368421
MMP9	36	67.874886	0.7368421
MMP2	34	42.788708	0.71794873
MAPK1	33	70.23675	0.70886075
FOS	32	92.012405	0.7
ERBB2	32	52.1215	0.7
CXCL8	32	35.600197	0.7
PPARG	31	87.4884	0.69135803
PLG	29	34.790962	0.67469877
ICAM1	28	19.573597	0.6666667
CDKN2A	27	49.359043	0.65882355
TIMP1	27	20.058231	0.65882355
MMP1	27	31.013016	0.65882355
BCL2L1	26	23.144257	0.6511628
HIF1A	25	26.408728	0.6436782
TLR2	25	27.443367	0.6436782
NOS3	23	14.741622	0.6292135
MMP3	23	20.958696	0.6292135
VCAM1	23	15.891444	0.62222224
CRP	23	16.9276	0.61538464
PGR	23	22.414396	0.6292135
CASP9	22	14.892768	0.62222224
IGF2	21	24.814903	0.61538464

**Table 3:** The top 10 active ingredients.

TCM	Ingedient	Degree	Betweenness
ZZ	Quercetin	31	2490.5723
LQ	Luteolin	14	599.8465
ZJC	Fisetin	11	480.52222
NBZ	Kaempferol	11	421.71927
HQ	Baicalein	10	331.7121
HQ	Wogonin	10	259.2096
CP	Nobiletin	8	233.14877
QP	Naringenin	7	277.06705
JYH	Beta-carotene	7	198.1999
NBZ	Beta-sitosterol	7	136.29044

**Table 4:** Basic information of the top 15 pathways with the most target genes.

ID	Term	Count	*P* value	Input
hsa05200	Pathways in cancer	22	4.08*E* − 26	BCL2L1/ERBB2/EGFR/HIF1A/RB1/MAPK1/MYC/IGF2/TCF7/CDKN2A/FOS/PPARG/VEGFA/CASP3/ESR1/CXCL8/CASP9/IL6/MMP9/MMP1/MMP2/BCL2
hsa05219	Bladder cancer	11	1.52*E* − 21	ERBB2/CDKN2A/CXCL8/EGFR/RB1/MAPK1/VEGFA/MMP9/MYC/MMP2/MMP1
hsa04933	AGE-RAGE signaling pathway in diabetic complications	12	1.25*E* − 19	NOS3/CASP3/CXCL8/IL6/MAPK1/VEGFA/VCAM1/ICAM1/SELE/COL3A1/MMP2/BCL2
hsa05161	Hepatitis B	12	3.09*E* − 17	PCNA/TLR2/CASP3/FOS/CXCL8/RB1/CASP9/IL6/MAPK1/MMP9/MYC/BCL2
hsa04151	PI3K-Akt signaling pathway	14	2.17*E* − 16	IGF2/BCL2L1/NOS3/ERBB2/EGFR/CASP9/IL6/INSR/MAPK1/VEGFA/MCL1/TLR2/MYC/BCL2
hsa05205	Proteoglycans in cancer	12	3.77*E* − 16	IGF2/ERBB2/CASP3/ESR1/EGFR/HIF1A/TLR2/MAPK1/VEGFA/MMP9/MYC/MMP2
hsa01522	Endocrine resistance	10	7.49*E* − 16	ERBB2/CDKN2A/ESR1/FOS/EGFR/RB1/MAPK1/MMP9/MMP2/BCL2
hsa04066	HIF-1 signaling pathway	10	2.03*E* − 15	ERBB2/NOS3/TIMP1/EGFR/HIF1A/IL6/INSR/MAPK1/VEGFA/BCL2
hsa04668	TNF signaling pathway	10	2.62*E* − 15	IRF1/CASP3/FOS/SELE/IL6/MAPK1/VCAM1/ICAM1/MMP9/MMP3
hsa05167	Kaposi sarcoma-associated herpesvirus infection	11	7.02*E* − 15	CASP3/FOS/CXCL8/HIF1A/CASP9/IL6/MAPK1/VEGFA/RB1/ICAM1/MYC
hsa04915	Estrogen signaling pathway	10	1.88*E* − 14	NOS3/FOS/EGFR/ESR1/CTSD/MAPK1/PGR/MMP9/MMP2/BCL2
hsa05206	MicroRNAs in cancer	12	3.14*E* − 14	ERBB2/TP63/CDKN2A/CASP3/CDC25C/EGFR/MAPK1/VEGFA/MCL1/MMP9/MYC/BCL2
hsa05215	Prostate cancer	9	5.09*E* − 14	ERBB2/TCF7/EGFR/RB1/CASP9/MAPK1/MMP9/MMP3/BCL2
hsa05212	Pancreatic cancer	8	4.92*E* − 13	BCL2L1/ERBB2/CDKN2A/EGFR/RB1/CASP9/MAPK1/VEGFA
hsa04926	Relaxin signaling pathway	9	6.12*E* − 13	NOS3/FOS/EGFR/MAPK1/VEGFA/MMP9/COL3A1/MMP2/MMP1

**Table 5:** Binding energy between active compound and target.

Compound	Affinity (kcal mol^−1^)			
	ALB	IL-6	EGFR	VEGFA
Quercetin	-6.82	-5.86	-7.29	-6.68
Luteolin	-7.06	-6.54	-5.82	-7.71
Fisetin	-6.41	-5.54	-6.69	-6.88
Kaempferol	-6.78	-6.99	-6.14	-7.88

## Data Availability

The data that supports the finding of this study is available in the supplementary material.

## Supplementary Materials

Supplementary Materials S1: 240 active components of Trichosanthis Niubang decoction (including repeated values). S2: PubChem CID information of 151 active components of Trichosanthes Niubang decoction (excluding duplication). S3: Venn diagram of intersection of drugs and diseases. S4: component-ingredient-disease-target gene network data. S5: G0 enrichment analysis (35 cell compositions). S6: G0 enrichment analysis (242 biological processes). S7: G0 enrichment analysis (59 molecular functions). S8: 200 KEGG pathway enrichment analyses.
