# Supplementary material for: Study the Mechanism of Gualou Niubang Decoction in Treating Plasma Cell Mastitis Based on Network Pharmacology and Molecular Docking
Source: Biomed Res Int. 2022 Jun 15;2022:5780936. doi: 10.1155/2022/5780936 (PMC9217541; doi:10.1155/2022/5780936)
Supplement: Supplementary Materials — S1: 240 active components of Trichosanthis Niubang decoction (including repeated values). S2: PubChem CID information of 151 active components of Trichosanthes Niubang decoction (excluding duplication). S3: Venn diagram of intersection of drugs and diseases. S4: component-ingredient-disease-target gene network data. S5: G0 enrichment analysis (35 cell compositions). S6: G0 enrichment analysis (242 biological processes). S7: G0 enrichment analysis (59 molecular functions). S8: 200 KEGG pathway enrichment analyses. [file 5780936.f1.zip › Table S 6 GO-BP.docx]

S 6 G0 enrichment analysis (242 biological process).

| Term | Count | PValue | Genes |
| --- | --- | --- | --- |
| GO:0043066~negative regulation of apoptotic process | 13 | 2.57E-08 | MMP9, EGFR, VEGFA, IL6, MYC, CASP3, ALB, BCL2, TIMP1, RAF1, TP63, MCL1, BCL2L1 |
| GO:0001666~response to hypoxia | 9 | 9.62E-08 | MUC1, VCAM1, CASP3, MMP2, CYP1A1, RAF1, HIF1A, TLR2, VEGFA |
| GO:0045893~positive regulation of transcription, DNA-templated | 13 | 9.96E-08 | RB1, CDKN2A, INSR, AHR, FOS, HIF1A, ESR1, IL6, MYC, IRF1, MAPK1, PPARG, TP63 |
| GO:0022617~extracellular matrix disassembly | 7 | 1.91E-07 | PRSS1, MMP1, MMP2, MMP3, PLG, TIMP1, MMP9 |
| GO:0010628~positive regulation of gene expression | 10 | 1.91E-07 | CRP, IL6, MYC, ERBB2, GBA, APOB, HIF1A, LDLR, TLR2, VEGFA |
| GO:0042060~wound healing | 7 | 2.60E-07 | COL3A1, IL6, CASP3, ERBB2, TIMP1, RAF1, EGFR |
| GO:0045944~positive regulation of transcription from RNA polymerase II promoter | 16 | 4.80E-07 | RB1, CDKN2A, AHR, FOS, HIF1A, ESR1, EGFR, VEGFA, IL6, MYC, IRF1, PGR, PPARG, RAF1, TP63, TLR2 |
| GO:0042493~response to drug | 10 | 6.66E-07 | IL6, POR, MYC, CASP3, BCL2, CYP1A1, PPARG, FOS, TOP1, ICAM1 |
| GO:0034097~response to cytokine | 6 | 7.93E-07 | COL3A1, BCL2, FOS, TIMP1, BCL2L1, MCL1 |
| GO:0032496~response to lipopolysaccharide | 8 | 1.16E-06 | CASP9, VCAM1, CASP3, CYP1A1, FOS, APOB, SELE, TLR2 |
| GO:0030574~collagen catabolic process | 6 | 2.26E-06 | COL3A1, MMP1, MMP2, MMP3, CTSD, MMP9 |
| GO:0008283~cell proliferation | 10 | 3.09E-06 | CHRM3, PCNA, MYC, ERBB2, BCL2, CYP1A1, CDC25C, RAF1, EGFR, BCL2L1 |
| GO:0097192~extrinsic apoptotic signaling pathway in absence of ligand | 5 | 4.77E-06 | CASP9, CASP3, BCL2, BCL2L1, MCL1 |
| GO:0009636~response to toxic substance | 6 | 9.20E-06 | PON1, BCL2, MAPK1, FOS, AHR, TLR2 |
| GO:0001541~ovarian follicle development | 5 | 1.13E-05 | MYC, BCL2, BCL2L1, ICAM1, VEGFA |
| GO:0045429~positive regulation of nitric oxide biosynthetic process | 5 | 1.24E-05 | IL6, INSR, ESR1, EGFR, ICAM1 |
| GO:0006915~apoptotic process | 11 | 1.56E-05 | RB1, CASP9, CDKN2A, CASP3, IRF1, BCL2, MAPK1, AHR, RAF1, TP63, TLR2 |
| GO:0007568~aging | 7 | 1.78E-05 | CASP9, COL3A1, IL6, VCAM1, CYP1A1, FOS, TIMP1 |
| GO:0008284~positive regulation of cell proliferation | 10 | 2.15E-05 | IL6, MYC, INSR, BCL2, IGF2, MAPK1, TIMP1, EGFR, BCL2L1, VEGFA |
| GO:0002576~platelet degranulation | 6 | 2.35E-05 | ALB, IGF2, PLG, TIMP1, CD36, VEGFA |
| GO:0071356~cellular response to tumor necrosis factor | 6 | 3.22E-05 | IL6, VCAM1, CXCL8, GBA, APOB, ICAM1 |
| GO:0008285~negative regulation of cell proliferation | 9 | 4.70E-05 | RB1, IL6, CXCL8, CDKN2A, NOS3, IRF1, PLG, RAF1, TLR2 |
| GO:0050679~positive regulation of epithelial cell proliferation | 5 | 4.71E-05 | IL6, MYC, ERBB2, EGFR, VEGFA |
| GO:2001243~negative regulation of intrinsic apoptotic signaling pathway | 4 | 5.17E-05 | BCL2, MMP9, BCL2L1, MCL1 |
| GO:0001934~positive regulation of protein phosphorylation | 6 | 6.41E-05 | INSR, ERBB2, IGF2, MMP9, EGFR, VEGFA |
| GO:0071347~cellular response to interleukin-1 | 5 | 9.13E-05 | IL6, CXCL8, MYC, HIF1A, ICAM1 |
| GO:0034349~glial cell apoptotic process | 3 | 1.09E-04 | RB1, CASP9, CASP3 |
| GO:0050731~positive regulation of peptidyl-tyrosine phosphorylation | 5 | 1.60E-04 | IL6, IGF2, CD36, ICAM1, VEGFA |
| GO:0046677~response to antibiotic | 4 | 1.63E-04 | CASP9, IL6, CASP3, CYP1A1 |
| GO:0051402~neuron apoptotic process | 4 | 1.79E-04 | RB1, BCL2, TP63, BCL2L1 |
| GO:0006338~chromatin remodeling | 5 | 1.92E-04 | RB1, MYC, TOP1, ESR1, TP63 |
| GO:0006508~proteolysis | 9 | 2.36E-04 | CASP9, PRSS1, MMP1, CASP3, MMP2, MMP3, PLG, CTSD, MMP9 |
| GO:0032355~response to estradiol | 5 | 2.39E-04 | CASP9, PCNA, MYC, CASP3, ESR1 |
| GO:0009812~flavonoid metabolic process | 3 | 3.02E-04 | POR, MYC, CYP1A1 |
| GO:0034383~low-density lipoprotein particle clearance | 3 | 3.02E-04 | CD36, APOB, LDLR |
| GO:0045740~positive regulation of DNA replication | 4 | 3.68E-04 | IL6, PCNA, INSR, EGFR |
| GO:0001701~in utero embryonic development | 6 | 3.91E-04 | MUC1, NOS3, MYC, APOB, BCL2L1, VEGFA |
| GO:0034644~cellular response to UV | 4 | 4.22E-04 | CASP9, PCNA, MYC, TP63 |
| GO:0008630~intrinsic apoptotic signaling pathway in response to DNA damage | 4 | 5.13E-04 | CASP9, BCL2, BCL2L1, MCL1 |
| GO:0071230~cellular response to amino acid stimulus | 4 | 5.13E-04 | COL3A1, MMP2, EGFR, BCL2L1 |
| GO:0042981~regulation of apoptotic process | 6 | 7.08E-04 | CASP9, RAF1, ESR1, TP63, BCL2L1, MCL1 |
| GO:0050900~leukocyte migration | 5 | 7.28E-04 | MMP1, APOB, SELE, MMP9, ICAM1 |
| GO:0048546~digestive tract morphogenesis | 3 | 8.32E-04 | BCL2, HIF1A, EGFR |
| GO:0045821~positive regulation of glycolytic process | 3 | 9.69E-04 | MYC, INSR, HIF1A |
| GO:0042953~lipoprotein transport | 3 | 0.001115473 | PPARG, CD36, APOB |
| GO:0010629~negative regulation of gene expression | 5 | 0.001121212 | RB1, GBA, PGR, ESR1, LDLR |
| GO:0007050~cell cycle arrest | 5 | 0.001247254 | RB1, CXCL8, CDKN2A, MYC, IRF1 |
| GO:0030301~cholesterol transport | 3 | 0.001272101 | CD36, APOB, LDLR |
| GO:0043627~response to estrogen | 4 | 0.001325534 | GBA, MAPK1, PPARG, ESR1 |
| GO:0051384~response to glucocorticoid | 4 | 0.001325534 | IL6, CASP3, GBA, BCL2 |
| GO:0007165~signal transduction | 12 | 0.001369755 | CHRM3, CXCL8, RASA1, ERBB2, MAPK1, PGR, PPARG, RAF1, HIF1A, ESR1, EGFR, TLR2 |
| GO:2000811~negative regulation of anoikis | 3 | 0.001438633 | BCL2, BCL2L1, MCL1 |
| GO:0070542~response to fatty acid | 3 | 0.001438633 | PON1, CD36, TLR2 |
| GO:0032722~positive regulation of chemokine production | 3 | 0.001438633 | IL6, HIF1A, TLR2 |
| GO:0043154~negative regulation of cysteine-type endopeptidase activity involved in apoptotic process | 4 | 0.001575077 | IL6, POR, RAF1, VEGFA |
| GO:0033138~positive regulation of peptidyl-serine phosphorylation | 4 | 0.001641736 | IL6, BCL2, RAF1, VEGFA |
| GO:0030855~epithelial cell differentiation | 4 | 0.001641736 | MUC1, PCNA, PPARG, VEGFA |
| GO:0000165~MAPK cascade | 6 | 0.001776781 | MYC, RASA1, ERBB2, MAPK1, RAF1, EGFR |
| GO:0008637~apoptotic mitochondrial changes | 3 | 0.001801147 | CDKN2A, BCL2L1, MCL1 |
| GO:0035902~response to immobilization stress | 3 | 0.002202497 | CYP1A1, PPARG, FOS |
| GO:0001503~ossification | 4 | 0.002406849 | BCL2, IGF2, MMP9, EGFR |
| GO:0070374~positive regulation of ERK1 and ERK2 cascade | 5 | 0.002745227 | IL6, CD36, EGFR, ICAM1, VEGFA |
| GO:0051897~positive regulation of protein kinase B signaling | 4 | 0.002765208 | IL6, INSR, IGF2, EGFR |
| GO:0050830~defense response to Gram-positive bacterium | 4 | 0.002859651 | CRP, IL6, CD36, TLR2 |
| GO:0007159~leukocyte cell-cell adhesion | 3 | 0.00311965 | VCAM1, SELE, ICAM1 |
| GO:0016032~viral process | 6 | 0.003148822 | RB1, VCAM1, MMP1, MAPK1, TOP1, CDC25C |
| GO:0007507~heart development | 5 | 0.003223751 | COL3A1, PCNA, ERBB2, PPARG, RAF1 |
| GO:0002053~positive regulation of mesenchymal cell proliferation | 3 | 0.003372412 | MYC, TP63, VEGFA |
| GO:0006898~receptor-mediated endocytosis | 5 | 0.003416997 | ALB, CD36, APOB, LDLR, EGFR |
| GO:0008584~male gonad development | 4 | 0.003799815 | INSR, BCL2, ESR1, BCL2L1 |
| GO:2000145~regulation of cell motility | 3 | 0.003905666 | ERBB2, RAF1, EGFR |
| GO:0071456~cellular response to hypoxia | 4 | 0.00403134 | BCL2, HIF1A, ICAM1, VEGFA |
| GO:0071549~cellular response to dexamethasone stimulus | 3 | 0.004186032 | CASP9, IL6, EGFR |
| GO:0097421~liver regeneration | 3 | 0.004186032 | PCNA, MYC, EGFR |
| GO:0010468~regulation of gene expression | 4 | 0.00451976 | MYC, BCL2, AHR, HIF1A |
| GO:0042593~glucose homeostasis | 4 | 0.004647216 | IL6, INSR, PPARG, HIF1A |
| GO:0043200~response to amino acid | 3 | 0.004773938 | IL6, CASP3, ICAM1 |
| GO:0071392~cellular response to estradiol stimulus | 3 | 0.004773938 | IL6, ESR1, EGFR |
| GO:0010332~response to gamma radiation | 3 | 0.004773938 | MYC, BCL2, TP63 |
| GO:0006974~cellular response to DNA damage stimulus | 5 | 0.005080679 | CASP9, MYC, BCL2, MAPK1, TP63 |
| GO:0031663~lipopolysaccharide-mediated signaling pathway | 3 | 0.005081353 | NOS3, MAPK1, TLR2 |
| GO:0071364~cellular response to epidermal growth factor stimulus | 3 | 0.005397664 | MYC, ERBB2, EGFR |
| GO:1902042~negative regulation of extrinsic apoptotic signaling pathway via death domain receptors | 3 | 0.005397664 | NOS3, RAF1, ICAM1 |
| GO:0048565~digestive tract development | 3 | 0.006056724 | RB1, COL3A1, CYP1A1 |
| GO:0071222~cellular response to lipopolysaccharide | 4 | 0.006348212 | IL6, CXCL8, CD36, ICAM1 |
| GO:0009409~response to cold | 3 | 0.006399355 | IL6, PPARG, FOS |
| GO:0010507~negative regulation of autophagy | 3 | 0.006399355 | BCL2, BCL2L1, MCL1 |
| GO:0001525~angiogenesis | 5 | 0.006483208 | CXCL8, NOS3, MMP2, HIF1A, VEGFA |
| GO:0045766~positive regulation of angiogenesis | 4 | 0.00666317 | CXCL8, NOS3, HIF1A, VEGFA |
| GO:0030168~platelet activation | 4 | 0.00666317 | COL3A1, IL6, MAPK1, RAF1 |
| GO:0035094~response to nicotine | 3 | 0.006750639 | VCAM1, CASP3, BCL2 |
| GO:2001240~negative regulation of extrinsic apoptotic signaling pathway in absence of ligand | 3 | 0.006750639 | BCL2, BCL2L1, MCL1 |
| GO:0006366~transcription from RNA polymerase II promoter | 7 | 0.007037152 | MYC, IRF1, FOS, AHR, HIF1A, ESR1, TP63 |
| GO:0044267~cellular protein metabolic process | 4 | 0.007152834 | MMP1, MMP2, IGF2, PLG |
| GO:0006954~inflammatory response | 6 | 0.008490093 | CRP, IL6, CXCL8, FOS, SELE, TLR2 |
| GO:0048538~thymus development | 3 | 0.009036764 | BCL2, MAPK1, RAF1 |
| GO:0000122~negative regulation of transcription from RNA polymerase II promoter | 8 | 0.009664699 | RB1, MYC, TCF7, PPARG, CD36, ESR1, TP63, VEGFA |
| GO:0032755~positive regulation of interleukin-6 production | 3 | 0.009865376 | IL6, CD36, TLR2 |
| GO:1990646~cellular response to prolactin | 2 | 0.009972028 | IL6, MYC |
| GO:0071221~cellular response to bacterial lipopeptide | 2 | 0.009972028 | CD36, TLR2 |
| GO:0019087~transformation of host cell by virus | 2 | 0.009972028 | MYC, INSR |
| GO:0001501~skeletal system development | 4 | 0.010746621 | COL3A1, IGF2, MMP9, TP63 |
| GO:0008360~regulation of cell shape | 4 | 0.011393619 | IL6, RASA1, ICAM1, VEGFA |
| GO:0007157~heterophilic cell-cell adhesion via plasma membrane cell adhesion molecules | 3 | 0.012078177 | VCAM1, SELE, ICAM1 |
| GO:0061419~positive regulation of transcription from RNA polymerase II promoter in response to hypoxia | 2 | 0.013274283 | HIF1A, VEGFA |
| GO:0071726~cellular response to diacyl bacterial lipopeptide | 2 | 0.013274283 | CD36, TLR2 |
| GO:0038124~toll-like receptor TLR6:TLR2 signaling pathway | 2 | 0.013274283 | CD36, TLR2 |
| GO:0042159~lipoprotein catabolic process | 2 | 0.013274283 | APOB, LDLR |
| GO:0070988~demethylation | 2 | 0.013274283 | POR, CYP1A1 |
| GO:0045727~positive regulation of translation | 3 | 0.013500481 | IL6, ERBB2, MAPK1 |
| GO:0048146~positive regulation of fibroblast proliferation | 3 | 0.01399002 | MYC, ESR1, EGFR |
| GO:0043401~steroid hormone mediated signaling pathway | 3 | 0.015504184 | PGR, PPARG, ESR1 |
| GO:0043406~positive regulation of MAP kinase activity | 3 | 0.016551042 | ERBB2, EGFR, VEGFA |
| GO:0022614~membrane to membrane docking | 2 | 0.01656572 | VCAM1, ICAM1 |
| GO:0033088~negative regulation of immature T cell proliferation in thymus | 2 | 0.01656572 | CDKN2A, ERBB2 |
| GO:0048661~positive regulation of smooth muscle cell proliferation | 3 | 0.017085535 | IL6, MYC, EGFR |
| GO:0006977~DNA damage response, signal transduction by p53 class mediator resulting in cell cycle arrest | 3 | 0.018176382 | MUC1, PCNA, CDC25C |
| GO:0070508~cholesterol import | 2 | 0.019846373 | CD36, LDLR |
| GO:0032025~response to cobalt ion | 2 | 0.019846373 | CASP9, CASP3 |
| GO:0050776~regulation of immune response | 4 | 0.021535535 | COL3A1, VCAM1, IRF1, ICAM1 |
| GO:0008203~cholesterol metabolic process | 3 | 0.021620051 | PON1, APOB, LDLR |
| GO:0071300~cellular response to retinoic acid | 3 | 0.022823604 | MUC1, MYC, PPARG |
| GO:0031647~regulation of protein stability | 3 | 0.022823604 | CDKN2A, BCL2, MAPK1 |
| GO:0010886~positive regulation of cholesterol storage | 2 | 0.023116277 | CD36, APOB |
| GO:0071312~cellular response to alkaloid | 2 | 0.023116277 | BCL2L1, ICAM1 |
| GO:0060333~interferon-gamma-mediated signaling pathway | 3 | 0.023435589 | VCAM1, IRF1, ICAM1 |
| GO:0030335~positive regulation of cell migration | 4 | 0.023471787 | INSR, MAPK1, EGFR, VEGFA |
| GO:0050729~positive regulation of inflammatory response | 3 | 0.024679722 | LDLR, EGFR, TLR2 |
| GO:0045892~negative regulation of transcription, DNA-templated | 6 | 0.025050871 | RB1, CDKN2A, IRF1, PPARG, AHR, TP63 |
| GO:0007584~response to nutrient | 3 | 0.025311771 | POR, VCAM1, PPARG |
| GO:2001020~regulation of response to DNA damage stimulus | 2 | 0.026375466 | CASP9, MCL1 |
| GO:0060100~positive regulation of phagocytosis, engulfment | 2 | 0.026375466 | PPARG, CD36 |
| GO:0010888~negative regulation of lipid storage | 2 | 0.026375466 | CRP, IL6 |
| GO:0032966~negative regulation of collagen biosynthetic process | 2 | 0.026375466 | IL6, PPARG |
| GO:0033993~response to lipid | 2 | 0.026375466 | PPARG, CD36 |
| GO:0009308~amine metabolic process | 2 | 0.026375466 | VCAM1, CYP1A1 |
| GO:0030324~lung development | 3 | 0.026595585 | NOS3, EGFR, VEGFA |
| GO:0032869~cellular response to insulin stimulus | 3 | 0.027247252 | MYC, INSR, PPARG |
| GO:0006805~xenobiotic metabolic process | 3 | 0.027905361 | CYP2C9, POR, AHR |
| GO:0014066~regulation of phosphatidylinositol 3-kinase signaling | 3 | 0.027905361 | ERBB2, MAPK1, EGFR |
| GO:0043589~skin morphogenesis | 2 | 0.029623975 | GBA, TP63 |
| GO:0032930~positive regulation of superoxide anion generation | 2 | 0.029623975 | CRP, EGFR |
| GO:0030299~intestinal cholesterol absorption | 2 | 0.029623975 | CD36, LDLR |
| GO:0008635~activation of cysteine-type endopeptidase activity involved in apoptotic process by cytochrome c | 2 | 0.029623975 | CASP9, CASP3 |
| GO:0097267~omega-hydroxylase P450 pathway | 2 | 0.029623975 | CYP2C9, CYP1A1 |
| GO:0002237~response to molecule of bacterial origin | 2 | 0.029623975 | CXCL8, TLR2 |
| GO:0071223~cellular response to lipoteichoic acid | 2 | 0.029623975 | CD36, TLR2 |
| GO:0043410~positive regulation of MAPK cascade | 3 | 0.029917852 | IL6, INSR, IGF2 |
| GO:0006919~activation of cysteine-type endopeptidase activity involved in apoptotic process | 3 | 0.031290837 | CDKN2A, CASP3, PPARG |
| GO:0030307~positive regulation of cell growth | 3 | 0.031986584 | ERBB2, BCL2, EGFR |
| GO:0046902~regulation of mitochondrial membrane permeability | 2 | 0.032861838 | BCL2, BCL2L1 |
| GO:0048013~ephrin receptor signaling pathway | 3 | 0.033396352 | RASA1, MMP2, MMP9 |
| GO:0007565~female pregnancy | 3 | 0.035555987 | MUC1, BCL2, FOS |
| GO:0002064~epithelial cell development | 2 | 0.03608909 | ESR1, TP63 |
| GO:0031669~cellular response to nutrient levels | 2 | 0.039305764 | IL6, ICAM1 |
| GO:0031000~response to caffeine | 2 | 0.039305764 | IL6, PPARG |
| GO:0007169~transmembrane receptor protein tyrosine kinase signaling pathway | 3 | 0.040798636 | INSR, ERBB2, EGFR |
| GO:0051926~negative regulation of calcium ion transport | 2 | 0.042511895 | NOS3, ICAM1 |
| GO:0050901~leukocyte tethering or rolling | 2 | 0.042511895 | VCAM1, SELE |
| GO:0097284~hepatocyte apoptotic process | 2 | 0.042511895 | RB1, BCL2L1 |
| GO:0071391~cellular response to estrogen stimulus | 2 | 0.042511895 | MYC, ESR1 |
| GO:0002526~acute inflammatory response | 2 | 0.042511895 | IL6, VCAM1 |
| GO:0010745~negative regulation of macrophage derived foam cell differentiation | 2 | 0.042511895 | CRP, PPARG |
| GO:0006469~negative regulation of protein kinase activity | 3 | 0.043129772 | RB1, IL6, CDKN2A |
| GO:0031175~neuron projection development | 3 | 0.043917732 | RB1, IL6, GBA |
| GO:0016337~single organismal cell-cell adhesion | 3 | 0.044711075 | VCAM1, EGFR, ICAM1 |
| GO:0000082~G1/S transition of mitotic cell cycle | 3 | 0.04550976 | RB1, PCNA, CDKN2A |
| GO:0032461~positive regulation of protein oligomerization | 2 | 0.045707516 | MMP1, MMP3 |
| GO:0019395~fatty acid oxidation | 2 | 0.045707516 | POR, PPARG |
| GO:0055114~oxidation-reduction process | 6 | 0.04687857 | CYP2C9, POR, VCAM1, NOS3, CYP1A1, CYP19A1 |
| GO:0051091~positive regulation of sequence-specific DNA binding transcription factor activity | 3 | 0.047937432 | IL6, PPARG, ESR1 |
| GO:0045471~response to ethanol | 3 | 0.047937432 | VCAM1, MYC, ICAM1 |
| GO:0001953~negative regulation of cell-matrix adhesion | 2 | 0.048892662 | CDKN2A, RASA1 |
| GO:0048714~positive regulation of oligodendrocyte differentiation | 2 | 0.048892662 | PPARG, TLR2 |
| GO:0050930~induction of positive chemotaxis | 2 | 0.048892662 | CXCL8, VEGFA |
| GO:0045725~positive regulation of glycogen biosynthetic process | 2 | 0.048892662 | INSR, IGF2 |
| GO:1904707~positive regulation of vascular smooth muscle cell proliferation | 2 | 0.048892662 | MMP2, MMP9 |
| GO:0006955~immune response | 5 | 0.051576726 | IL6, CXCL8, TCF7, CD36, TLR2 |
| GO:0008209~androgen metabolic process | 2 | 0.052067366 | ESR1, CYP19A1 |
| GO:0035994~response to muscle stretch | 2 | 0.052067366 | FOS, RAF1 |
| GO:0048011~neurotrophin TRK receptor signaling pathway | 2 | 0.052067366 | CASP3, RAF1 |
| GO:0030949~positive regulation of vascular endothelial growth factor receptor signaling pathway | 2 | 0.052067366 | HIF1A, VEGFA |
| GO:0006978~DNA damage response, signal transduction by p53 class mediator resulting in transcription of p21 class mediator | 2 | 0.052067366 | MUC1, TP63 |
| GO:0010744~positive regulation of macrophage derived foam cell differentiation | 2 | 0.052067366 | CD36, APOB |
| GO:0060749~mammary gland alveolus development | 2 | 0.055231662 | ESR1, VEGFA |
| GO:0030224~monocyte differentiation | 2 | 0.055231662 | PPARG, VEGFA |
| GO:0016925~protein sumoylation | 3 | 0.058102223 | PCNA, CDKN2A, TOP1 |
| GO:0019373~epoxygenase P450 pathway | 2 | 0.058385583 | CYP2C9, CYP1A1 |
| GO:0030225~macrophage differentiation | 2 | 0.058385583 | MMP9, VEGFA |
| GO:0060324~face development | 2 | 0.058385583 | MAPK1, RAF1 |
| GO:0033189~response to vitamin A | 2 | 0.058385583 | CYP1A1, PPARG |
| GO:0006357~regulation of transcription from RNA polymerase II promoter | 5 | 0.059220272 | RB1, TCF7, FOS, AHR, VEGFA |
| GO:0030220~platelet formation | 2 | 0.061529163 | CASP9, CASP3 |
| GO:0046716~muscle cell cellular homeostasis | 2 | 0.061529163 | IL6, HIF1A |
| GO:0007263~nitric oxide mediated signal transduction | 2 | 0.061529163 | NOS3, CD36 |
| GO:0030308~negative regulation of cell growth | 3 | 0.06164365 | CDKN2A, BCL2, PPARG |
| GO:0051726~regulation of cell cycle | 3 | 0.064347329 | RB1, IRF1, CDC25C |
| GO:1902895~positive regulation of pri-miRNA transcription from RNA polymerase II promoter | 2 | 0.064662435 | FOS, HIF1A |
| GO:0002052~positive regulation of neuroblast proliferation | 2 | 0.064662435 | HIF1A, VEGFA |
| GO:0014911~positive regulation of smooth muscle cell migration | 2 | 0.064662435 | MYC, BCL2 |
| GO:0001892~embryonic placenta development | 2 | 0.064662435 | HIF1A, EGFR |
| GO:0048844~artery morphogenesis | 2 | 0.067785432 | APOB, VEGFA |
| GO:2000378~negative regulation of reactive oxygen species metabolic process | 2 | 0.067785432 | BCL2, HIF1A |
| GO:0007399~nervous system development | 4 | 0.070685927 | CHRM3, FOS, APOB, VEGFA |
| GO:0051000~positive regulation of nitric-oxide synthase activity | 2 | 0.070898187 | HIF1A, ESR1 |
| GO:0071542~dopaminergic neuron differentiation | 2 | 0.070898187 | HIF1A, VEGFA |
| GO:0010165~response to X-ray | 2 | 0.070898187 | CASP3, TP63 |
| GO:0043524~negative regulation of neuron apoptotic process | 3 | 0.071748197 | RASA1, BCL2, BCL2L1 |
| GO:0051092~positive regulation of NF-kappaB transcription factor activity | 3 | 0.072692168 | IL6, ICAM1, TLR2 |
| GO:0001836~release of cytochrome c from mitochondria | 2 | 0.074000733 | BCL2, BCL2L1 |
| GO:0016239~positive regulation of macroautophagy | 2 | 0.074000733 | GBA, HIF1A |
| GO:0000083~regulation of transcription involved in G1/S transition of mitotic cell cycle | 2 | 0.074000733 | RB1, PCNA |
| GO:0035924~cellular response to vascular endothelial growth factor stimulus | 2 | 0.074000733 | VCAM1, VEGFA |
| GO:0002092~positive regulation of receptor internalization | 2 | 0.077093104 | SELE, VEGFA |
| GO:0034612~response to tumor necrosis factor | 2 | 0.080175331 | CASP3, SELE |
| GO:2000134~negative regulation of G1/S transition of mitotic cell cycle | 2 | 0.080175331 | RB1, BCL2 |
| GO:0051090~regulation of sequence-specific DNA binding transcription factor activity | 2 | 0.080175331 | MAPK1, FOS |
| GO:0032735~positive regulation of interleukin-12 production | 2 | 0.080175331 | CD36, TLR2 |
| GO:0030878~thyroid gland development | 2 | 0.080175331 | MAPK1, RAF1 |
| GO:0007569~cell aging | 2 | 0.080175331 | BCL2, ICAM1 |
| GO:0007202~activation of phospholipase C activity | 2 | 0.083247448 | SELE, EGFR |
| GO:0050999~regulation of nitric-oxide synthase activity | 2 | 0.083247448 | NOS3, EGFR |
| GO:0045840~positive regulation of mitotic nuclear division | 2 | 0.083247448 | INSR, IGF2 |
| GO:0032728~positive regulation of interferon-beta production | 2 | 0.086309486 | IRF1, TLR2 |
| GO:0031069~hair follicle morphogenesis | 2 | 0.086309486 | BCL2, TP63 |
| GO:0017144~drug metabolic process | 2 | 0.086309486 | CYP2C9, CYP1A1 |
| GO:0035987~endodermal cell differentiation | 2 | 0.086309486 | MMP2, MMP9 |
| GO:0040007~growth | 2 | 0.08936148 | BCL2L1, VEGFA |
| GO:0009314~response to radiation | 2 | 0.08936148 | COL3A1, BCL2 |
| GO:0061418~regulation of transcription from RNA polymerase II promoter in response to hypoxia | 2 | 0.08936148 | HIF1A, VEGFA |
| GO:0051881~regulation of mitochondrial membrane potential | 2 | 0.08936148 | BCL2, BCL2L1 |
| GO:0043388~positive regulation of DNA binding | 2 | 0.08936148 | MYC, MMP9 |
| GO:0006367~transcription initiation from RNA polymerase II promoter | 3 | 0.091359899 | PGR, PPARG, ESR1 |
| GO:0018108~peptidyl-tyrosine phosphorylation | 3 | 0.092378517 | INSR, ERBB2, EGFR |
| GO:0051973~positive regulation of telomerase activity | 2 | 0.09240346 | MYC, MAPK1 |
| GO:0045909~positive regulation of vasodilation | 2 | 0.09240346 | NOS3, EGFR |
| GO:0006260~DNA replication | 3 | 0.09442584 | PCNA, TOP1, CDC25C |
| GO:0044344~cellular response to fibroblast growth factor stimulus | 2 | 0.095435459 | CXCL8, MYC |
| GO:1900740~positive regulation of protein insertion into mitochondrial membrane involved in apoptotic signaling pathway | 2 | 0.095435459 | BCL2, TP63 |
| GO:0045739~positive regulation of DNA repair | 2 | 0.095435459 | PCNA, EGFR |
| GO:0006629~lipid metabolic process | 3 | 0.096486397 | PPARG, CD36, LDLR |
| GO:0045765~regulation of angiogenesis | 2 | 0.09845751 | IL6, ERBB2 |
