# Supplementary material for: Study the Mechanism of Gualou Niubang Decoction in Treating Plasma Cell Mastitis Based on Network Pharmacology and Molecular Docking
Source: Biomed Res Int. 2022 Jun 15;2022:5780936. doi: 10.1155/2022/5780936 (PMC9217541; doi:10.1155/2022/5780936)
Supplement: Supplementary Materials — S1: 240 active components of Trichosanthis Niubang decoction (including repeated values). S2: PubChem CID information of 151 active components of Trichosanthes Niubang decoction (excluding duplication). S3: Venn diagram of intersection of drugs and diseases. S4: component-ingredient-disease-target gene network data. S5: G0 enrichment analysis (35 cell compositions). S6: G0 enrichment analysis (242 biological processes). S7: G0 enrichment analysis (59 molecular functions). S8: 200 KEGG pathway enrichment analyses. [file 5780936.f1.zip › Table S 7 GO-MF.docx]

S 7 G0 enrichment analysis (59 molecular function).

| Term | Count | PValue | Genes |
| --- | --- | --- | --- |
| GO:0005515~protein binding | 51 | 1.13E-08 | RB1, CHRM3, CXCL8, AHR, ICAM1, CASP9, MYC, CASP3, TIMP1, CD36, CTSD, TP63, MMP2, MMP3, FOS, CDC25C, MMP9, POR, RASA1, IRF1, PPARG, PGR, TOP1, RAF1, TLR2, CRP, PCNA, TCF7, GBA, PLG, HIF1A, EGFR, MUC1, ERBB2, MAPK1, APOB, LDLR, MCL1, CDKN2A, NOS3, INSR, IGF2, SELE, ESR1, VEGFA, COL3A1, IL6, ALB, CYP1A1, BCL2, BCL2L1 |
| GO:0042802~identical protein binding | 15 | 1.32E-07 | RB1, PCNA, ESR1, MMP9, EGFR, VEGFA, ERBB2, ALB, BCL2, MAPK1, PPARG, RAF1, LDLR, TP63, BCL2L1 |
| GO:0008134~transcription factor binding | 10 | 4.22E-07 | RB1, CDKN2A, MYC, BCL2, MAPK1, PPARG, FOS, AHR, HIF1A, ESR1 |
| GO:0046982~protein heterodimerization activity | 11 | 3.09E-06 | ERBB2, BCL2, FOS, AHR, RAF1, HIF1A, EGFR, BCL2L1, TLR2, MCL1, VEGFA |
| GO:0019899~enzyme binding | 9 | 1.50E-05 | POR, PCNA, CYP1A1, PGR, PPARG, RAF1, HIF1A, ESR1, EGFR |
| GO:0043565~sequence-specific DNA binding | 10 | 5.45E-05 | MYC, IRF1, TCF7, BCL2, PGR, PPARG, FOS, HIF1A, ESR1, TP63 |
| GO:0051434~BH3 domain binding | 3 | 6.69E-05 | BCL2, BCL2L1, MCL1 |
| GO:0001077~transcriptional activator activity, RNA polymerase II core promoter proximal region sequence-specific binding | 7 | 1.40E-04 | MYC, IRF1, PGR, FOS, HIF1A, ESR1, TP63 |
| GO:0004252~serine-type endopeptidase activity | 7 | 2.13E-04 | PRSS1, MMP1, MMP2, MMP3, PLG, CTSD, MMP9 |
| GO:0004716~receptor signaling protein tyrosine kinase activity | 3 | 4.95E-04 | INSR, ERBB2, EGFR |
| GO:0019825~oxygen binding | 4 | 5.33E-04 | CYP2C9, ALB, CYP1A1, CYP19A1 |
| GO:0030169~low-density lipoprotein particle binding | 3 | 0.001143459 | CRP, CD36, LDLR |
| GO:0003682~chromatin binding | 7 | 0.001990598 | PCNA, PPARG, FOS, TOP1, ESR1, TP63, EGFR |
| GO:0008144~drug binding | 4 | 0.002154841 | CYP2C9, CHRM3, ALB, PPARG |
| GO:0003677~DNA binding | 14 | 0.00326617 | RB1, PCNA, CDKN2A, AHR, FOS, ESR1, MYC, IRF1, ALB, MAPK1, PGR, PPARG, TOP1, TP63 |
| GO:0008395~steroid hydroxylase activity | 3 | 0.003455958 | CYP2C9, CYP1A1, CYP19A1 |
| GO:0005496~steroid binding | 3 | 0.003724371 | PGR, SHBG, ESR1 |
| GO:0003700~transcription factor activity, sequence-specific DNA binding | 10 | 0.004622633 | RB1, MYC, IRF1, PGR, PPARG, FOS, AHR, HIF1A, ESR1, TP63 |
| GO:0002020~protease binding | 4 | 0.004813301 | CASP3, BCL2, TIMP1, LDLR |
| GO:0005178~integrin binding | 4 | 0.005363179 | COL3A1, VCAM1, EGFR, ICAM1 |
| GO:0044212~transcription regulatory region DNA binding | 5 | 0.005774568 | TCF7, PPARG, FOS, AHR, TP63 |
| GO:0004888~transmembrane signaling receptor activity | 5 | 0.005869974 | ERBB2, SELE, EGFR, ICAM1, TLR2 |
| GO:0004879~RNA polymerase II transcription factor activity, ligand-activated sequence-specific DNA binding | 3 | 0.006556063 | PPARG, AHR, ESR1 |
| GO:0004222~metalloendopeptidase activity | 4 | 0.006572643 | MMP1, MMP2, MMP3, MMP9 |
| GO:0000978~RNA polymerase II core promoter proximal region sequence-specific DNA binding | 6 | 0.006842666 | MUC1, MYC, IRF1, PGR, FOS, ESR1 |
| GO:0004714~transmembrane receptor protein tyrosine kinase activity | 3 | 0.007284236 | INSR, ERBB2, EGFR |
| GO:0019901~protein kinase binding | 6 | 0.008660487 | CASP9, CDKN2A, CDC25C, HIF1A, EGFR, BCL2L1 |
| GO:0001046~core promoter sequence-specific DNA binding | 3 | 0.009256263 | PPARG, TOP1, ESR1 |
| GO:0020037~heme binding | 4 | 0.011118322 | CYP2C9, NOS3, CYP1A1, CYP19A1 |
| GO:0004175~endopeptidase activity | 3 | 0.014325486 | MMP1, MMP3, MMP9 |
| GO:0005506~iron ion binding | 4 | 0.014946907 | CYP2C9, NOS3, CYP1A1, CYP19A1 |
| GO:0003707~steroid hormone receptor activity | 3 | 0.015350573 | PGR, PPARG, ESR1 |
| GO:0004497~monooxygenase activity | 3 | 0.016406341 | CYP2C9, CYP1A1, CYP19A1 |
| GO:0008083~growth factor activity | 4 | 0.017389491 | IL6, IGF2, TIMP1, VEGFA |
| GO:0004197~cysteine-type endopeptidase activity | 3 | 0.018046551 | CASP9, CASP3, CTSD |
| GO:0019903~protein phosphatase binding | 3 | 0.019177087 | ERBB2, PPARG, EGFR |
| GO:0001948~glycoprotein binding | 3 | 0.020336767 | RASA1, LDLR, EGFR |
| GO:0002039~p53 binding | 3 | 0.02152516 | MUC1, CDKN2A, TP63 |
| GO:0030235~nitric-oxide synthase regulator activity | 2 | 0.026701 | ESR1, EGFR |
| GO:0016491~oxidoreductase activity | 4 | 0.030032948 | CYP2C9, POR, NOS3, CYP1A1 |
| GO:0008237~metallopeptidase activity | 3 | 0.030612694 | MMP2, MMP3, MMP9 |
| GO:0003690~double-stranded DNA binding | 3 | 0.030612694 | MYC, TP63, EGFR |
| GO:0004872~receptor activity | 4 | 0.036908028 | CHRM3, LDLR, ICAM1, TLR2 |
| GO:0005041~low-density lipoprotein receptor activity | 2 | 0.043032111 | CD36, LDLR |
| GO:0097153~cysteine-type endopeptidase activity involved in apoptotic process | 2 | 0.043032111 | CASP9, CASP3 |
| GO:0000989~transcription factor activity, transcription factor binding | 2 | 0.046265877 | HIF1A, TP63 |
| GO:0016301~kinase activity | 4 | 0.047870762 | CDKN2A, ERBB2, MAPK1, RAF1 |
| GO:0005159~insulin-like growth factor receptor binding | 2 | 0.049488907 | INSR, IGF2 |
| GO:0016712~oxidoreductase activity, acting on paired donors, with incorporation or reduction of molecular oxygen, reduced flavin or flavoprotein as one donor, and incorporation of one atom of oxygen | 2 | 0.049488907 | CYP1A1, CYP19A1 |
| GO:0010181~FMN binding | 2 | 0.049488907 | POR, NOS3 |
| GO:0050750~low-density lipoprotein particle receptor binding | 2 | 0.052701235 | CRP, APOB |
| GO:0004709~MAP kinase kinase kinase activity | 2 | 0.071752424 | RAF1, EGFR |
| GO:0031625~ubiquitin protein ligase binding | 4 | 0.072804002 | RB1, BCL2, HIF1A, EGFR |
| GO:0004713~protein tyrosine kinase activity | 3 | 0.074277385 | INSR, ERBB2, EGFR |
| GO:0070330~aromatase activity | 2 | 0.087340459 | CYP1A1, CYP19A1 |
| GO:0051879~Hsp90 protein binding | 2 | 0.087340459 | AHR, HIF1A |
| GO:0035035~histone acetyltransferase binding | 2 | 0.090427061 | PCNA, HIF1A |
| GO:0046983~protein dimerization activity | 3 | 0.091240512 | MYC, ERBB2, AHR |
| GO:0050699~WW domain binding | 2 | 0.099625463 | CDC25C, TP63 |
