# Supplementary material for: Study the Mechanism of Gualou Niubang Decoction in Treating Plasma Cell Mastitis Based on Network Pharmacology and Molecular Docking
Source: Biomed Res Int. 2022 Jun 15;2022:5780936. doi: 10.1155/2022/5780936 (PMC9217541; doi:10.1155/2022/5780936)
Supplement: Supplementary Materials — S1: 240 active components of Trichosanthis Niubang decoction (including repeated values). S2: PubChem CID information of 151 active components of Trichosanthes Niubang decoction (excluding duplication). S3: Venn diagram of intersection of drugs and diseases. S4: component-ingredient-disease-target gene network data. S5: G0 enrichment analysis (35 cell compositions). S6: G0 enrichment analysis (242 biological processes). S7: G0 enrichment analysis (59 molecular functions). S8: 200 KEGG pathway enrichment analyses. [file 5780936.f1.zip › Table S 8 KEGG Enrichment pathways.docx]

S8 200 KEGG pathway enrichment analysis.

| Term | ID | P-Value | Gene |
| --- | --- | --- | --- |
| Pathways in cancer | hsa05200 | 4.08E-26 | BCL2L1，ERBB2，EGFR，HIF1A，RB1，MAPK1，MYC，IGF2，TCF7，CDKN2A，FOS，PPARG，VEGFA，CASP3，ESR1，CXCL8，CASP9，IL6，MMP9，MMP1，MMP2，BCL2 |
| Bladder cancer | hsa05219 | 1.52E-21 | ERBB2，CDKN2A，CXCL8，EGFR，RB1，MAPK1，VEGFA，MMP9，MYC，MMP2，MMP1 |
| AGE-RAGE signaling pathway in diabetic complications | hsa04933 | 1.25E-19 | NOS3，CASP3，CXCL8，IL6，MAPK1，VEGFA，VCAM1，ICAM1，SELE，COL3A1，MMP2，BCL2 |
| Hepatitis B | hsa05161 | 3.09E-17 | PCNA，TLR2，CASP3，FOS，CXCL8，RB1，CASP9，IL6，MAPK1，MMP9，MYC，BCL2 |
| PI3K-Akt signaling pathway | hsa04151 | 2.17E-16 | IGF2，BCL2L1，NOS3，ERBB2，EGFR，CASP9，IL6，INSR，MAPK1，VEGFA，MCL1，TLR2，MYC，BCL2 |
| Proteoglycans in cancer | hsa05205 | 3.77E-16 | IGF2，ERBB2，CASP3，ESR1，EGFR，HIF1A，TLR2，MAPK1，VEGFA，MMP9，MYC，MMP2 |
| Endocrine resistance | hsa01522 | 7.49E-16 | ERBB2，CDKN2A，ESR1，FOS，EGFR，RB1，MAPK1，MMP9，MMP2，BCL2 |
| HIF-1 signaling pathway | hsa04066 | 2.03E-15 | ERBB2，NOS3，TIMP1，EGFR，HIF1A，IL6，INSR，MAPK1，VEGFA，BCL2 |
| TNF signaling pathway | hsa04668 | 2.62E-15 | IRF1，CASP3，FOS，SELE，IL6，MAPK1，VCAM1，ICAM1，MMP9，MMP3 |
| Kaposi sarcoma-associated herpesvirus infection | hsa05167 | 7.02E-15 | CASP3，FOS，CXCL8，HIF1A，CASP9，IL6，MAPK1，VEGFA，RB1，ICAM1，MYC |
| Estrogen signaling pathway | hsa04915 | 1.88E-14 | NOS3，FOS，EGFR，ESR1，CTSD，MAPK1，PGR，MMP9，MMP2，BCL2 |
| MicroRNAs in cancer | hsa05206 | 3.14E-14 | ERBB2，TP63，CDKN2A，CASP3，CDC25C，EGFR，MAPK1，VEGFA，MCL1，MMP9，MYC，BCL2 |
| Prostate cancer | hsa05215 | 5.09E-14 | ERBB2，TCF7，EGFR，RB1，CASP9，MAPK1，MMP9，MMP3，BCL2 |
| Pancreatic cancer | hsa05212 | 4.92E-13 | BCL2L1，ERBB2，CDKN2A，EGFR，RB1，CASP9，MAPK1，VEGFA |
| Relaxin signaling pathway | hsa04926 | 6.12E-13 | NOS3，FOS，EGFR，MAPK1，VEGFA，MMP9，COL3A1，MMP2，MMP1 |
| Fluid shear stress and atherosclerosis | hsa05418 | 1.08E-12 | NOS3，FOS，SELE，VEGFA，VCAM1，ICAM1，MMP9，MMP2，BCL2 |
| Colorectal cancer | hsa05210 | 1.37E-12 | TCF7，CASP3，FOS，EGFR，CASP9，MAPK1，MYC，BCL2 |
| Breast cancer | hsa05224 | 1.75E-12 | ERBB2，TCF7，ESR1，FOS，EGFR，RB1，MAPK1，PGR，MYC |
| Human cytomegalovirus infection | hsa05163 | 1.96E-12 | CDKN2A，CASP3，CXCL8，EGFR，RB1，CASP9，IL6，MAPK1，VEGFA，MYC |
| Rheumatoid arthritis | hsa05323 | 2.10E-12 | TLR2，FOS，CXCL8，IL6，VEGFA，ICAM1，MMP3，MMP1 |
| Malaria | hsa05144 | 2.30E-12 | TLR2，CXCL8，CD36，IL6，VCAM1，ICAM1，SELE |
| IL-17 signaling pathway | hsa04657 | 2.47E-12 | CASP3，FOS，CXCL8，IL6，MAPK1，MMP9，MMP3，MMP1 |
| MAPK signaling pathway | hsa04010 | 2.57E-11 | IGF2，ERBB2，CASP3，FOS，EGFR，INSR，MAPK1，VEGFA，MYC，RASA1 |
| Platinum drug resistance | hsa01524 | 3.06E-11 | BCL2L1，ERBB2，CDKN2A，CASP3，CASP9，MAPK1，BCL2 |
| Apoptosis | hsa04210 | 4.38E-11 | BCL2L1，CASP3，FOS，CTSD，CASP9，MAPK1，MCL1，BCL2 |
| EGFR tyrosine kinase inhibitor resistance | hsa01521 | 5.14E-11 | BCL2L1，ERBB2，EGFR，IL6，MAPK1，VEGFA，BCL2 |
| Influenza A | hsa05164 | 2.09E-10 | PLG，PRSS1，CASP3，CXCL8，CASP9，IL6，MAPK1，ICAM1 |
| Hepatocellular carcinoma | hsa05225 | 2.18E-10 | IGF2，BCL2L1，TCF7，CDKN2A，EGFR，RB1，MAPK1，MYC |
| Toxoplasmosis | hsa05145 | 5.48E-10 | BCL2L1，CASP3，CASP9，TLR2，LDLR，MAPK1，BCL2 |
| Endometrial cancer | hsa05213 | 5.55E-10 | ERBB2，TCF7，EGFR，CASP9，MAPK1，MYC |
| Epstein-Barr virus infection | hsa05169 | 8.53E-10 | TLR2，CASP3，RB1，CASP9，IL6，ICAM1，MYC，BCL2 |
| Non-small cell lung cancer | hsa05223 | 1.15E-09 | ERBB2，CDKN2A，EGFR，RB1，CASP9，MAPK1 |
| Human immunodeficiency virus 1 infection | hsa05170 | 1.28E-09 | BCL2L1，CASP3，FOS，CDC25C，CASP9，TLR2，MAPK1，BCL2 |
| Human T-cell leukemia virus 1 infection | hsa05166 | 1.64E-09 | BCL2L1，CDKN2A，FOS，RB1，IL6，MAPK1，ICAM1，MYC |
| Measles | hsa05162 | 2.07E-09 | BCL2L1，TLR2，CASP3，FOS，CASP9，IL6，BCL2 |
| Pertussis | hsa05133 | 2.54E-09 | IRF1，CASP3，FOS，CXCL8，IL6，MAPK1 |
| Gastric cancer | hsa05226 | 3.44E-09 | ERBB2，TCF7，EGFR，RB1，MAPK1，MYC，BCL2 |
| Cushing syndrome | hsa04934 | 4.47E-09 | TCF7，CDKN2A，EGFR，RB1，AHR，LDLR，MAPK1 |
| Hepatitis C | hsa05160 | 4.47E-09 | CASP3，EGFR，RB1，CASP9，LDLR，MAPK1，MYC |
| Small cell lung cancer | hsa05222 | 7.98E-09 | BCL2L1，CASP3，RB1，CASP9，MYC，BCL2 |
| Tuberculosis | hsa05152 | 1.16E-08 | TLR2，CASP3，CTSD，CASP9，IL6，MAPK1，BCL2 |
| Transcriptional misregulation in cancer | hsa05202 | 1.50E-08 | BCL2L1，CXCL8，IL6，PPARG，MMP9，MYC，MMP3 |
| Legionellosis | hsa05134 | 3.01E-08 | IL6，CXCL8，TLR2，CASP3，CASP9 |
| Ras signaling pathway | hsa04014 | 6.50E-08 | IGF2，BCL2L1，EGFR，INSR，MAPK1，VEGFA，RASA1 |
| Central carbon metabolism in cancer | hsa05230 | 8.74E-08 | ERBB2，MYC，MAPK1，EGFR，HIF1A |
| Adherens junction | hsa04520 | 1.07E-07 | ERBB2，TCF7，MAPK1，EGFR，INSR |
| p53 signaling pathway | hsa04115 | 1.07E-07 | BCL2L1，CASP9，CDKN2A，CASP3，BCL2 |
| Chronic myeloid leukemia | hsa05220 | 1.38E-07 | BCL2L1，MYC，MAPK1，CDKN2A，RB1 |
| Cellular senescence | hsa04218 | 1.75E-07 | CDKN2A，CXCL8，RB1，IL6，MAPK1，MYC |
| Jak-STAT signaling pathway | hsa04630 | 1.88E-07 | BCL2L1，EGFR，IL6，MCL1，MYC，BCL2 |
| Apoptosis - multiple species | hsa04215 | 2.72E-07 | BCL2L1，CASP9，CASP3，BCL2 |
| PD-L1 expression and PD-1 checkpoint pathway in cancer | hsa05235 | 2.91E-07 | TLR2，MAPK1，EGFR，HIF1A，FOS |
| Amoebiasis | hsa05146 | 3.97E-07 | IL6，COL3A1，TLR2，CASP3，CXCL8 |
| African trypanosomiasis | hsa05143 | 4.15E-07 | IL6，SELE，VCAM1，ICAM1 |
| Thyroid cancer | hsa05216 | 4.15E-07 | PPARG，MYC，MAPK1，TCF7 |
| NF-kappa B signaling pathway | hsa04064 | 5.06E-07 | BCL2L1，CXCL8，VCAM1，ICAM1，BCL2 |
| Chagas disease (American trypanosomiasis) | hsa05142 | 5.82E-07 | IL6，CXCL8，MAPK1，TLR2，FOS |
| Toll-like receptor signaling pathway | hsa04620 | 6.10E-07 | IL6，CXCL8，MAPK1，TLR2，FOS |
| Human papillomavirus infection | hsa05165 | 6.62E-07 | TCF7，CASP3，EGFR，IRF1，RB1，MAPK1，VEGFA |
| Th17 cell differentiation | hsa04659 | 6.98E-07 | IL6，AHR，MAPK1，HIF1A，FOS |
| Thyroid hormone signaling pathway | hsa04919 | 1.16E-06 | CASP9，MYC，MAPK1，HIF1A，ESR1 |
| Ovarian steroidogenesis | hsa04913 | 1.18E-06 | CYP1A1，LDLR，CYP19A1，INSR |
| Amyotrophic lateral sclerosis (ALS) | hsa05014 | 1.38E-06 | BCL2L1，CASP9，CASP3，BCL2 |
| Cell cycle | hsa04110 | 1.40E-06 | CDC25C，PCNA，MYC，CDKN2A，RB1 |
| Autophagy - animal | hsa04140 | 1.63E-06 | BCL2L1，MAPK1，CTSD，HIF1A，BCL2 |
| VEGF signaling pathway | hsa04370 | 2.38E-06 | MAPK1，VEGFA，NOS3，CASP9 |
| Prolactin signaling pathway | hsa04917 | 4.55E-06 | MAPK1，IRF1，ESR1，FOS |
| Melanoma | hsa05218 | 5.06E-06 | CDKN2A，MAPK1，EGFR，RB1 |
| Glioma | hsa05214 | 5.91E-06 | CDKN2A，MAPK1，EGFR，RB1 |
| NOD-like receptor signaling pathway | hsa04621 | 7.77E-06 | IL6，CXCL8，MAPK1，BCL2L1，BCL2 |
| Salmonella infection | hsa05132 | 8.67E-06 | IL6，CXCL8，MAPK1，FOS |
| ErbB signaling pathway | hsa04012 | 9.49E-06 | ERBB2，MYC，MAPK1，EGFR |
| Focal adhesion | hsa04510 | 1.31E-05 | ERBB2，EGFR，MAPK1，VEGFA，BCL2 |
| Choline metabolism in cancer | hsa05231 | 1.69E-05 | MAPK1，EGFR，HIF1A，FOS |
| Parathyroid hormone synthesis, secretion and action | hsa04928 | 2.19E-05 | MAPK1，BCL2，EGFR，FOS |
| Insulin resistance | hsa04931 | 2.35E-05 | IL6，INSR，NOS3，CD36 |
| Cholinergic synapse | hsa04725 | 2.70E-05 | MAPK1，BCL2，CHRM3，FOS |
| Leukocyte transendothelial migration | hsa04670 | 2.70E-05 | MMP2，VCAM1，ICAM1，MMP9 |
| Sphingolipid signaling pathway | hsa04071 | 3.40E-05 | MAPK1，CTSD，NOS3，BCL2 |
| Yersinia infection | hsa05135 | 3.62E-05 | IL6，CXCL8，MAPK1，FOS |
| FoxO signaling pathway | hsa04068 | 5.03E-05 | IL6，INSR，MAPK1，EGFR |
| Cholesterol metabolism | hsa04979 | 6.78E-05 | LDLR，APOB，CD36 |
| Phospholipase D signaling pathway | hsa04072 | 7.76E-05 | CXCL8，INSR，MAPK1，EGFR |
| Non-alcoholic fatty liver disease (NAFLD) | hsa04932 | 7.95E-05 | IL6，CXCL8，INSR，CASP3 |
| Oxytocin signaling pathway | hsa04921 | 8.79E-05 | MAPK1，EGFR，NOS3，FOS |
| Herpes simplex virus 1 infection | hsa05168 | 9.31E-05 | BCL2L1，TLR2，CASP3，CASP9，IL6，BCL2 |
| Viral myocarditis | hsa05416 | 0.000113728 | CASP3，ICAM1，CASP9 |
| Acute myeloid leukemia | hsa05221 | 0.000149111 | MYC，MAPK1，TCF7 |
| Renal cell carcinoma | hsa05211 | 0.0001692 | MAPK1，VEGFA，HIF1A |
| Epithelial cell signaling in Helicobacter pylori infection | hsa05120 | 0.000176267 | CXCL8，EGFR，CASP3 |
| Leishmaniasis | hsa05140 | 0.000206445 | TLR2，MAPK1，FOS |
| Calcium signaling pathway | hsa04020 | 0.000210205 | ERBB2，EGFR，NOS3，CHRM3 |
| PPAR signaling pathway | hsa03320 | 0.000222712 | PPARG，CD36，MMP1 |
| Viral carcinogenesis | hsa05203 | 0.000244633 | CDKN2A，MAPK1，CASP3，RB1 |
| Rap1 signaling pathway | hsa04015 | 0.000288017 | EGFR，INSR，MAPK1，VEGFA |
| GnRH signaling pathway | hsa04912 | 0.000395306 | EGFR，MAPK1，MMP2 |
| Progesterone-mediated oocyte maturation | hsa04914 | 0.000472072 | CDC25C，MAPK1，PGR |
| C-type lectin receptor signaling pathway | hsa04625 | 0.000542872 | IL6，IRF1，MAPK1 |
| Serotonergic synapse | hsa04726 | 0.000721697 | CYP2C9，MAPK1，CASP3 |
| AMPK signaling pathway | hsa04152 | 0.000813978 | PPARG，INSR，CD36 |
| Platelet activation | hsa04611 | 0.000892963 | COL3A1，MAPK1，NOS3 |
| Osteoclast differentiation | hsa04380 | 0.000976655 | PPARG，MAPK1，FOS |
| Oocyte meiosis | hsa04114 | 0.000976655 | CDC25C，MAPK1，PGR |
| Natural killer cell mediated cytotoxicity | hsa04650 | 0.001042576 | MAPK1，CASP3，ICAM1 |
| Signaling pathways regulating pluripotency of stem cells | hsa04550 | 0.001257026 | MYC，MAPK1，TCF7 |
| Prion diseases | hsa05020 | 0.001378913 | IL6，MAPK1 |
| Cell adhesion molecules (CAMs) | hsa04514 | 0.001414327 | SELE，VCAM1，ICAM1 |
| Aldosterone-regulated sodium reabsorption | hsa04960 | 0.001531293 | INSR，MAPK1 |
| Fat digestion and absorption | hsa04975 | 0.001859016 | APOB，CD36 |
| cGMP-PKG signaling pathway | hsa04022 | 0.002060289 | INSR，MAPK1，NOS3 |
| Alzheimer disease | hsa05010 | 0.002200891 | MAPK1，CASP3，CASP9 |
| Type II diabetes mellitus | hsa04930 | 0.002311265 | INSR，MAPK1 |
| Huntington disease | hsa05016 | 0.003080544 | PPARG，CASP3，CASP9 |
| Steroid hormone biosynthesis | hsa00140 | 0.003823702 | CYP1A1，CYP19A1 |
| Regulation of actin cytoskeleton | hsa04810 | 0.004096474 | EGFR，MAPK1，CHRM3 |
| Mitophagy - animal | hsa04137 | 0.004449697 | BCL2L1，HIF1A |
| Inflammatory bowel disease (IBD) | hsa05321 | 0.004449697 | IL6，TLR2 |
| Retinol metabolism | hsa00830 | 0.004712481 | CYP2C9，CYP1A1 |
| Shigellosis | hsa05131 | 0.004846506 | CXCL8，MAPK1 |
| Staphylococcus aureus infection | hsa05150 | 0.004846506 | PLG，ICAM1 |
| Metabolism of xenobiotics by cytochrome P450 | hsa00980 | 0.005981206 | CYP2C9，CYP1A1 |
| B cell receptor signaling pathway | hsa04662 | 0.00690405 | MAPK1，FOS |
| Chemical carcinogenesis | hsa05204 | 0.00690405 | CYP2C9，CYP1A1 |
| Gap junction | hsa04540 | 0.007887215 | MAPK1，EGFR |
| Longevity regulating pathway | hsa04211 | 0.008056863 | PPARG，INSR |
| Protein digestion and absorption | hsa04974 | 0.008228151 | PRSS1，COL3A1 |
| Th1 and Th2 cell differentiation | hsa04658 | 0.008575627 | MAPK1，FOS |
| TGF-beta signaling pathway | hsa04350 | 0.008929603 | MYC，MAPK1 |
| Hematopoietic cell lineage | hsa04640 | 0.009472666 | IL6，CD36 |
| Circadian entrainment | hsa04713 | 0.009472666 | MAPK1，FOS |
| Pancreatic secretion | hsa04972 | 0.009656894 | PRSS1，CHRM3 |
| Viral protein interaction with cytokine and cytokine receptor | hsa04061 | 0.01003013 | IL6，CXCL8 |
| Melanogenesis | hsa04916 | 0.010219128 | TCF7，MAPK1 |
| T cell receptor signaling pathway | hsa04660 | 0.01060186 | MAPK1，FOS |
| Neurotrophin signaling pathway | hsa04722 | 0.013886605 | MAPK1，BCL2 |
| Neuroactive ligand-receptor interaction | hsa04080 | 0.014052715 | PLG，PRSS1，CHRM3 |
| Insulin signaling pathway | hsa04910 | 0.018038076 | INSR，MAPK1 |
| Apelin signaling pathway | hsa04371 | 0.018038076 | MAPK1，NOS3 |
| Parkinson disease | hsa05012 | 0.01927349 | CASP3，CASP9 |
| Adrenergic signaling in cardiomyocytes | hsa04261 | 0.02106122 | MAPK1，BCL2 |
| Phagosome | hsa04145 | 0.021847825 | TLR2，CD36 |
| mTOR signaling pathway | hsa04150 | 0.022112718 | INSR，MAPK1 |
| Hippo signaling pathway | hsa04390 | 0.02237895 | MYC，TCF7 |
| Wnt signaling pathway | hsa04310 | 0.02400421 | MYC，TCF7 |
| Tight junction | hsa04530 | 0.02681718 | ERBB2，PCNA |
| Axon guidance | hsa04360 | 0.030057458 | MAPK1，RASA1 |
| Arginine biosynthesis | hsa00220 | 0.031976786 | NOS3 |
| Chemokine signaling pathway | hsa04062 | 0.032818612 | CXCL8，MAPK1 |
| Mismatch repair | hsa03430 | 0.034833436 | PCNA |
| Vitamin digestion and absorption | hsa04977 | 0.036258653 | APOB |
| cAMP signaling pathway | hsa04024 | 0.040642218 | MAPK1，FOS |
| Linoleic acid metabolism | hsa00591 | 0.043353771 | CYP2C9 |
| Asthma | hsa05310 | 0.046177422 | RAF1 |
| Antifolate resistance | hsa01523 | 0.046177422 | IL6 |
| Base excision repair | hsa03410 | 0.048992882 | PCNA |
| Endocytosis | hsa04144 | 0.051297539 | LDLR，EGFR |
| DNA replication | hsa03030 | 0.053200765 | PCNA |
| Graft-versus-host disease | hsa05332 | 0.060173278 | IL6 |
| Tryptophan metabolism | hsa00380 | 0.061561712 | CYP1A1 |
| Hedgehog signaling pathway | hsa04340 | 0.068473706 | BCL2 |
| Nucleotide excision repair | hsa03420 | 0.068473706 | PCNA |
| Cytokine-cytokine receptor interaction | hsa04060 | 0.070965672 | IL6，CXCL8 |
| Intestinal immune network for IgA production | hsa04672 | 0.071224473 | IL6 |
| Endocrine and other factor-regulated calcium reabsorption | hsa04961 | 0.072596861 | ESR1 |
| Arginine and proline metabolism | hsa00330 | 0.072596861 | NOS3 |
| Regulation of lipolysis in adipocytes | hsa04923 | 0.079428964 | INSR |
| Long-term depression | hsa04730 | 0.086211597 | MAPK1 |
| Longevity regulating pathway - multiple species | hsa04213 | 0.088910877 | INSR |
| Basal cell carcinoma | hsa05217 | 0.090257578 | TCF7 |
| Cytosolic DNA-sensing pathway | hsa04623 | 0.090257578 | IL6 |
| Arachidonic acid metabolism | hsa00590 | 0.090257578 | CYP2C9 |
| Cortisol synthesis and secretion | hsa04927 | 0.092945112 | LDLR |
| Long-term potentiation | hsa04720 | 0.095624843 | MAPK1 |
| Amphetamine addiction | hsa05031 | 0.09696179 | FOS |
| Fc epsilon RI signaling pathway | hsa04664 | 0.09696179 | MAPK1 |
| Adipocytokine signaling pathway | hsa04920 | 0.098296794 | CD36 |
| RIG-I-like receptor signaling pathway | hsa04622 | 0.099629858 | CXCL8 |
| Bile secretion | hsa04976 | 0.102290179 | LDLR |
| Drug metabolism - cytochrome P450 | hsa00982 | 0.102290179 | CYP2C9 |
| Thyroid hormone synthesis | hsa04918 | 0.104942775 | ALB |
| Gastric acid secretion | hsa04971 | 0.106266183 | CHRM3 |
| Arrhythmogenic right ventricular cardiomyopathy (ARVC) | hsa05412 | 0.108907232 | TCF7 |
| Complement and coagulation cascades | hsa04610 | 0.111540611 | PLG |
| Taste transduction | hsa04742 | 0.116784445 | CHRM3 |
| ECM-receptor interaction | hsa04512 | 0.120697349 | CD36 |
| Insulin secretion | hsa04911 | 0.120697349 | CHRM3 |
| Salivary secretion | hsa04970 | 0.125888063 | CHRM3 |
| Hypertrophic cardiomyopathy (HCM) | hsa05410 | 0.125888063 | IL6 |
| Fc gamma R-mediated phagocytosis | hsa04666 | 0.13104866 | MAPK1 |
| Aldosterone synthesis and secretion | hsa04925 | 0.136179313 | LDLR |
| Glutamatergic synapse | hsa04724 | 0.156405873 | MAPK1 |
| Metabolic pathways | hsa01100 | 0.161972614 | CYP2C9，CYP1A1，CYP19A1，NOS3 |
| Lysosome | hsa04142 | 0.167578009 | CTSD |
| Dopaminergic synapse | hsa04728 | 0.17738664 | FOS |
| Vascular smooth muscle contraction | hsa04270 | 0.178604704 | MAPK1 |
| Retrograde endocannabinoid signaling | hsa04723 | 0.197854378 | MAPK1 |
| Necroptosis | hsa04217 | 0.214333668 | BCL2 |
| Protein processing in endoplasmic reticulum | hsa04141 | 0.217821415 | BCL2 |
| Alcoholism | hsa05034 | 0.235033195 | MAPK1 |
| Thermogenesis | hsa04714 | 0.29081506 | PPARG |
