# Supplementary material for: Study the Mechanism of Gualou Niubang Decoction in Treating Plasma Cell Mastitis Based on Network Pharmacology and Molecular Docking
Source: Biomed Res Int. 2022 Jun 15;2022:5780936. doi: 10.1155/2022/5780936 (PMC9217541; doi:10.1155/2022/5780936)
Supplement: Supplementary Materials — S1: 240 active components of Trichosanthis Niubang decoction (including repeated values). S2: PubChem CID information of 151 active components of Trichosanthes Niubang decoction (excluding duplication). S3: Venn diagram of intersection of drugs and diseases. S4: component-ingredient-disease-target gene network data. S5: G0 enrichment analysis (35 cell compositions). S6: G0 enrichment analysis (242 biological processes). S7: G0 enrichment analysis (59 molecular functions). S8: 200 KEGG pathway enrichment analyses. [file 5780936.f1.zip › Table S1 Ingredients (including duplicate values) (1).docx]

S 1 240 Active Components of Trichosanthis Niubang Decoction (including repeated values).

| Herb | | [Mol ID](https://old.tcmsp-e.com/tcmspsearch.php?qr=Arum Ternatum Thunb.&qsr=herb_en_name&token=0a62180c0cce4570733651f856e9e670) | [Molecule Name](https://old.tcmsp-e.com/tcmspsearch.php?qr=Arum Ternatum Thunb.&qsr=herb_en_name&token=0a62180c0cce4570733651f856e9e670) | | | [OB (%)](https://old.tcmsp-e.com/tcmspsearch.php?qr=Arum Ternatum Thunb.&qsr=herb_en_name&token=0a62180c0cce4570733651f856e9e670) | | [DL](https://old.tcmsp-e.com/tcmspsearch.php?qr=Arum Ternatum Thunb.&qsr=herb_en_name&token=0a62180c0cce4570733651f856e9e670) | |
| --- | --- | --- | --- | --- | --- | --- | --- | --- | --- |
| Niu Bangzi | | MOL010868 | neoarctin A | | | 39.99 | | 0.27 | |
| Niu Bangzi | | MOL000522 | arctiin | | | 34.45 | | 0.84 | |
| Niu Bangzi | | MOL000358 | beta-sitosterol | | | 36.91 | | 0.75 | |
| Niu Bangzi | | MOL000422 | kaempferol | | | 41.88 | | 0.24 | |
| Niu Bangzi | | MOL001506 | Supraene | | | 33.55 | | 0.42 | |
| Niu Bangzi | | MOL002773 | beta-carotene | | | 37.18 | | 0.58 | |
| Niu Bangzi | | MOL003290 | (3R,4R)-3,4-bis[(3,4-dimethoxyphenyl)methyl]oxolan-2-one | | | 52.3 | | 0.48 | |
| Niu Bangzi | | MOL007326 | Cynarin(e) | | | 31.76 | | 0.68 | |
| Tian Huafen | | MOL004355 | Spinasterol | | | 42.98 | | 0.76 | |
| Tian Huafen | | MOL006756 | Schottenol | | | 37.42 | | 0.75 | |
| Zhizi | | MOL001406 | crocetin | | | 35.3 | | 0.26 | |
| Zhizi | | MOL001663 | (4aS,6aR,6aS,6bR,8aR,10R,12aR,14bS)-10-hydroxy-2,2,6a,6b,9,9,12a-heptamethyl-1,3,4,5,6,6a,7,8,8a,10,11,12,13,14b-tetradecahydropicene-4a-carboxylic acid | | | 32.03 | | 0.76 | |
| Zhizi | | MOL001941 | Ammidin | | | 34.55 | | 0.22 | |
| Zhizi | | MOL004561 | Sudan III | | | 84.07 | | 0.59 | |
| Zhizi | | MOL000098 | quercetin | | | 46.43 | | 0.28 | |
| Zhizi | | MOL000358 | beta-sitosterol | | | 36.91 | | 0.75 | |
| Zhizi | | MOL000422 | kaempferol | | | 41.88 | | 0.24 | |
| Zhizi | | MOL000449 | Stigmasterol | | | 43.83 | | 0.76 | |
| Zhizi | | MOL001494 | Mandenol | | | 42 | | 0.19 | |
| Zhizi | | MOL001506 | Supraene | | | 33.55 | | 0.42 | |
| Zhizi | | MOL001942 | isoimperatorin | | | 45.46 | | 0.23 | |
| Zhizi | | MOL002883 | Ethyl oleate (NF) | | | 32.4 | | 0.19 | |
| Zhizi | | MOL003095 | 5-hydroxy-7-methoxy-2-(3,4,5-trimethoxyphenyl)chromone | | | 51.96 | | 0.41 | |
| Zhizi | | MOL007245 | 3-Methylkempferol | | | 60.16 | | 0.26 | |
| Zhizi | | MOL009038 | GBGB | | | 45.58 | | 0.83 | |
| Zao Jiaoci | | MOL013179 | fisetin | | | 52.6 | | 0.24 | |
| Zao Jiaoci | | MOL013296 | Fustin | | | 50.91 | | 0.24 | |
| Zao Jiaoci | | MOL001736 | (-)-taxifolin | | | 60.51 | | 0.27 | |
| Zao Jiaoci | | MOL002914 | Eriodyctiol (flavanone) | | | 41.35 | | 0.24 | |
| Zao Jiaoci | | MOL000358 | beta-sitosterol | | | 36.91 | | 0.75 | |
| Zao Jiaoci | | MOL000359 | sitosterol | | | 36.91 | | 0.75 | |
| Zao Jiaoci | | MOL000422 | kaempferol | | | 41.88 | | 0.24 | |
| Zao Jiaoci | | MOL000449 | Stigmasterol | | | 43.83 | | 0.76 | |
| Zao Jiaoci | | MOL006358 | Stigmast-4-ene-3,6-dione | | | 39.12 | | 0.79 | |
| Zao Jiaoci | | MOL000073 | ent-Epicatechin | | | 48.96 | | 0.24 | |
| Zao Jiaoci | | MOL000098 | quercetin | | | 46.43 | | 0.28 | |
| Qingpi | | MOL001798 | neohesperidin_qt | | | 71.17 | | 0.27 | |
| Qingpi | | MOL001803 | Sinensetin | | | 50.56 | | 0.45 | |
| Qingpi | | MOL004328 | naringenin | | | 59.29 | | 0.21 | |
| Qingpi | | MOL005100 | 5,7-dihydroxy-2-(3-hydroxy-4-methoxyphenyl)chroman-4-one | | | 47.74 | | 0.27 | |
| Qingpi | | MOL005828 | nobiletin | | | 61.67 | | 0.52 | |
| Chaihu | | MOL001645 | Linoleyl acetate | | | 42.1 | | 0.2 | |
| Chaihu | | MOL002776 | Baicalin | | | 40.12 | | 0.75 | |
| Chaihu | | MOL000449 | Stigmasterol | | | 43.83 | | 0.76 | |
| Chaihu | | MOL000354 | isorhamnetin | | | 49.6 | | 0.31 | |
| Chaihu | | MOL000422 | kaempferol | | | 41.88 | | 0.24 | |
| Chaihu | | MOL004598 | 3,5,6,7-tetramethoxy-2-(3,4,5-trimethoxyphenyl)chromone | | | 31.97 | | 0.59 | |
| Chaihu | | MOL004609 | Areapillin | | | 48.96 | | 0.41 | |
| Chaihu | | MOL013187 | Cubebin | | | 57.13 | | 0.64 | |
| Chaihu | | MOL004624 | Longikaurin A | | | 47.72 | | 0.53 | |
| Chaihu | | MOL004628 | Octalupine | | | 47.82 | | 0.28 | |
| Chaihu | | MOL004644 | Sainfuran | | | 79.91 | | 0.23 | |
| Chaihu | | MOL004648 | Troxerutin | | | 31.6 | | 0.28 | |
| Chaihu | | MOL004653 | (+)-Anomalin | | | 46.06 | | 0.66 | |
| Chaihu | | MOL004702 | saikosaponin c_qt | | | 30.5 | | 0.63 | |
| Chaihu | | MOL004718 | α-spinasterol | | | 42.98 | | 0.76 | |
| Chaihu | | MOL000490 | petunidin | | | 30.05 | | 0.31 | |
| Chaihu | | MOL000098 | quercetin | | | 46.43 | | 0.28 | |
| Huangqin | | MOL001689 | acacetin | | | 34.97 | | 0.24 | |
| Huangqin | | MOL000173 | wogonin | | | 30.68 | | 0.23 | |
| Huangqin | | MOL000228 | (2R)-7-hydroxy-5-methoxy-2-phenylchroman-4-one | | | 55.23 | | 0.2 | |
| Huangqin | | MOL002714 | baicalein | | | 33.52 | | 0.21 | |
| Huangqin | | MOL002908 | 5,8,2'-Trihydroxy-7-methoxyflavone | | | 37.01 | | 0.27 | |
| Huangqin | | MOL002909 | 5,7,2,5-tetrahydroxy-8,6-dimethoxyflavone | | | 33.82 | | 0.45 | |
| Huangqin | | MOL002910 | Carthamidin | | | 41.15 | | 0.24 | |
| Huangqin | | MOL002911 | 2,6,2',4'-tetrahydroxy-6'-methoxychaleone | | | 69.04 | | 0.22 | |
| Huangqin | | MOL002913 | Dihydrobaicalin_qt | | | 40.04 | | 0.21 | |
| Huangqin | | MOL002914 | Eriodyctiol (flavanone) | | | 41.35 | | 0.24 | |
| Huangqin | | MOL002915 | Salvigenin | | | 49.07 | | 0.33 | |
| Huangqin | | MOL002917 | 5,2',6'-Trihydroxy-7,8-dimethoxyflavone | | | 45.05 | | 0.33 | |
| Huangqin | | MOL002925 | 5,7,2',6'-Tetrahydroxyflavone | | | 37.01 | | 0.24 | |
| Huangqin | | MOL002926 | dihydrooroxylin A | | | 38.72 | | 0.23 | |
| Huangqin | | MOL002927 | Skullcapflavone II | | | 69.51 | | 0.44 | |
| Huangqin | | MOL002928 | oroxylin a | | | 41.37 | | 0.23 | |
| Huangqin | | MOL002932 | Panicolin | | | 76.26 | | 0.29 | |
| Huangqin | | MOL002933 | 5,7,4'-Trihydroxy-8-methoxyflavone | | | 36.56 | | 0.27 | |
| Huangqin | | MOL002934 | NEOBAICALEIN | | | 104.34 | | 0.44 | |
| Huangqin | | MOL002937 | DIHYDROOROXYLIN | | | 66.06 | | 0.23 | |
| Huangqin | | MOL000358 | beta-sitosterol | | | 36.91 | | 0.75 | |
| Huangqin | | MOL000359 | sitosterol | | | 36.91 | | 0.75 | |
| Huangqin | | MOL000525 | Norwogonin | | | 39.4 | | 0.21 | |
| Huangqin | | MOL000552 | 5,2'-Dihydroxy-6,7,8-trimethoxyflavone | | | 31.71 | | 0.35 | |
| Huangqin | | MOL000073 | ent-Epicatechin | | | 48.96 | | 0.24 | |
| Huangqin | | MOL000449 | Stigmasterol | | | 43.83 | | 0.76 | |
| Huangqin | | MOL001458 | coptisine | | | 30.67 | | 0.86 | |
| Huangqin | | MOL001490 | bis[(2S)-2-ethylhexyl] benzene-1,2-dicarboxylate | | | 43.59 | | 0.35 | |
| Huangqin | | MOL001506 | Supraene | | | 33.55 | | 0.42 | |
| Huangqin | | MOL002879 | Diop | | | 43.59 | | 0.39 | |
| Huangqin | | MOL002897 | epiberberine | | | 43.09 | | 0.78 | |
| Huangqin | | MOL008206 | Moslosooflavone | | | 44.09 | | 0.25 | |
| Huangqin | | MOL010415 | 11,13-Eicosadienoic acid, methyl ester | | | 39.28 | | 0.23 | |
| Huangqin | | MOL012245 | 5,7,4'-trihydroxy-6-methoxyflavanone | | | 36.63 | | 0.27 | |
| Huangqin | | MOL012246 | 5,7,4'-trihydroxy-8-methoxyflavanone | | | 74.24 | | 0.26 | |
| Huangqin | | MOL012266 | rivularin | | | 37.94 | | 0.37 | |
| Jin Yinhua | | MOL001494 | Mandenol | | | 42 | | 0.19 | |
| Jin Yinhua | | MOL001495 | Ethyl linolenate | | | 46.1 | | 0.2 | |
| Jin Yinhua | | MOL002707 | phytofluene | | | 43.18 | | 0.5 | |
| Jin Yinhua | | MOL002914 | Eriodyctiol (flavanone) | | | 41.35 | | 0.24 | |
| Jin Yinhua | | MOL003006 | (-)-(3R,8S,9R,9aS,10aS)-9-ethenyl-8-(beta-D-glucopyranosyloxy)-2,3,9,9a,10,10a-hexahydro-5-oxo-5H,8H-pyrano[4,3-d]oxazolo[3,2-a]pyridine-3-carboxylic acid_qt | | | 87.47 | | 0.23 | |
| Jin Yinhua | | MOL003014 | secologanic dibutylacetal_qt | | | 53.65 | | 0.29 | |
| Jin Yinhua | | MOL002773 | beta-carotene | | | 37.18 | | 0.58 | |
| Jin Yinhua | | MOL003036 | ZINC03978781 | | | 43.83 | | 0.76 | |
| Jin Yinhua | | MOL003044 | Chryseriol | | | 35.85 | | 0.27 | |
| Jin Yinhua | | MOL003059 | kryptoxanthin | | | 47.25 | | 0.57 | |
| Jin Yinhua | | MOL003062 | 4,5'-Retro-.beta.,.beta.-Carotene-3,3'-dione, 4',5'-didehydro- | | | 31.22 | | 0.55 | |
| Jin Yinhua | | MOL003095 | 5-hydroxy-7-methoxy-2-(3,4,5-trimethoxyphenyl)chromone | | | 51.96 | | 0.41 | |
| Jin Yinhua | | MOL003101 | 7-epi-Vogeloside | | | 46.13 | | 0.58 | |
| Jin Yinhua | | MOL003108 | Caeruloside C | | | 55.64 | | 0.73 | |
| Jin Yinhua | | MOL003111 | Centauroside_qt | | | 55.79 | | 0.5 | |
| Jin Yinhua | | MOL003117 | Ioniceracetalides B_qt | | | 61.19 | | 0.19 | |
| Jin Yinhua | | MOL003124 | XYLOSTOSIDINE | | | 43.17 | | 0.64 | |
| Jin Yinhua | | MOL003128 | dinethylsecologanoside | | | 48.46 | | 0.48 | |
| Jin Yinhua | | MOL000358 | beta-sitosterol | | | 36.91 | | 0.75 | |
| Jin Yinhua | | MOL000422 | kaempferol | | | 41.88 | | 0.24 | |
| Jin Yinhua | | MOL000449 | Stigmasterol | | | 43.83 | | 0.76 | |
| Jin Yinhua | | MOL000006 | luteolin | | | 36.16 | | 0.25 | |
| Jin Yinhua | | MOL000098 | quercetin | | | 46.43 | | 0.28 | |
| Lianqiao | | MOL000173 | wogonin | | | 30.68 | | 0.23 | |
| Lianqiao | | MOL003281 | 20(S)-dammar-24-ene-3β,20-diol-3-acetate | | | 40.23 | | 0.82 | |
| Lianqiao | | MOL003283 | (2R,3R,4S)-4-(4-hydroxy-3-methoxy-phenyl)-7-methoxy-2,3-dimethylol-tetralin-6-ol | | | 66.51 | | 0.39 | |
| Lianqiao | | MOL003290 | (3R,4R)-3,4-bis[(3,4-dimethoxyphenyl)methyl]oxolan-2-one | | | 52.3 | | 0.48 | |
| Lianqiao | | MOL003295 | (+)-pinoresinol monomethyl ether | | | 53.08 | | 0.57 | |
| Lianqiao | | MOL003305 | PHILLYRIN | | | 36.4 | | 0.86 | |
| Lianqiao | | MOL003306 | ACon1_001697 | | | 85.12 | | 0.57 | |
| Lianqiao | | MOL003308 | (+)-pinoresinol monomethyl ether-4-D-beta-glucoside_qt | | | 61.2 | | 0.57 | |
| Lianqiao | | MOL003315 | 3beta-Acetyl-20,25-epoxydammarane-24alpha-ol | | | 33.07 | | 0.79 | |
| Lianqiao | | MOL000211 | Mairin | | | 55.38 | | 0.78 | |
| Lianqiao | | MOL003322 | FORSYTHINOL | | | 81.25 | | 0.57 | |
| Lianqiao | | MOL003330 | (-)-Phillygenin | | | 95.04 | | 0.57 | |
| Lianqiao | | MOL003344 | β-amyrin acetate | | | 42.06 | | 0.74 | |
| Lianqiao | | MOL003347 | hyperforin | | | 44.03 | | 0.6 | |
| Lianqiao | | MOL003348 | adhyperforin | | | 44.03 | | 0.61 | |
| Lianqiao | | MOL003365 | Lactucasterol | | | 40.99 | | 0.85 | |
| Lianqiao | | MOL003370 | Onjixanthone I | | | 79.16 | | 0.3 | |
| Lianqiao | | MOL000358 | beta-sitosterol | | | 36.91 | | 0.75 | |
| Lianqiao | | MOL000422 | kaempferol | | | 41.88 | | 0.24 | |
| Lianqiao | | MOL000522 | arctiin | | | 34.45 | | 0.84 | |
| Lianqiao | | MOL000006 | luteolin | | | 36.16 | | 0.25 | |
| Lianqiao | | MOL000791 | bicuculline | | | 69.67 | | 0.88 | |
| Lianqiao | | MOL000098 | quercetin | | | 46.43 | | 0.28 | |
| Chenpi | | MOL000359 | sitosterol | | | 36.91 | | 0.75 | |
| Chenpi | | MOL004328 | naringenin | | | 59.29 | | 0.21 | |
| Chenpi | | MOL005100 | 5,7-dihydroxy-2-(3-hydroxy-4-methoxyphenyl)chroman-4-one | | | 47.74 | | 0.27 | |
| Chenpi | | MOL005815 | Citromitin | | | 86.9 | | 0.51 | |
| Chenpi | | MOL005828 | nobiletin | | | 61.67 | | 0.52 | |
| Gancao | | MOL001484 | Inermine | | | 75.18 | | 0.54 | |
| Gancao | | MOL001792 | DFV | | | 32.76 | | 0.18 | |
| Gancao | | MOL000211 | Mairin | | | 55.38 | | 0.78 | |
| Gancao | | MOL002311 | Glycyrol | | | 90.78 | | 0.67 | |
| Gancao | | MOL000239 | Jaranol | | | 50.83 | | 0.29 | |
| Gancao | | MOL002565 | Medicarpin | | | 49.22 | | 0.34 | |
| Gancao | | MOL000354 | isorhamnetin | | | 49.6 | | 0.31 | |
| Gancao | | MOL000359 | sitosterol | | | 36.91 | | 0.75 | |
| Gancao | | MOL003656 | Lupiwighteone | | | 51.64 | | 0.37 | |
| Gancao | | MOL003896 | 7-Methoxy-2-methyl isoflavone | | | 42.56 | | 0.2 | |
| Gancao | | MOL000392 | formononetin | | | 69.67 | | 0.21 | |
| Gancao | | MOL000417 | Calycosin | | | 47.75 | | 0.24 | |
| Gancao | | MOL000422 | kaempferol | | | 41.88 | | 0.24 | |
| Gancao | | MOL004328 | naringenin | | | 59.29 | | 0.21 | |
| Gancao | | MOL004805 | (2S)-2-[4-hydroxy-3-(3-methylbut-2-enyl)phenyl]-8,8-dimethyl-2,3-dihydropyrano[2,3-f]chromen-4-one | | | 31.79 | | 0.72 | |
| Gancao | | MOL004806 | euchrenone | | | 30.29 | | 0.57 | |
| Gancao | | MOL004808 | glyasperin B | | | 65.22 | | 0.44 | |
| Gancao | | MOL004810 | glyasperin F | | | 75.84 | | 0.54 | |
| Gancao | | MOL004811 | Glyasperin C | | | 45.56 | | 0.4 | |
| Gancao | | MOL004814 | Isotrifoliol | | | 31.94 | | 0.42 | |
| Gancao | | MOL004815 | (E)-1-(2,4-dihydroxyphenyl)-3-(2,2-dimethylchromen-6-yl)prop-2-en-1-one | | | 39.62 | | 0.35 | |
| Gancao | | MOL004820 | kanzonols W | | | 50.48 | | 0.52 | |
| Gancao | | MOL004824 | (2S)-6-(2,4-dihydroxyphenyl)-2-(2-hydroxypropan-2-yl)-4-methoxy-2,3-dihydrofuro[3,2-g]chromen-7-one | | | 60.25 | | 0.63 | |
| Gancao | | MOL004827 | Semilicoisoflavone B | | | 48.78 | | 0.55 | |
| Gancao | | MOL004828 | Glepidotin A | | | 44.72 | | 0.35 | |
| Gancao | | MOL004829 | Glepidotin B | | | 64.46 | | 0.34 | |
| Gancao | | MOL004833 | Phaseolinisoflavan | | | 32.01 | | 0.45 | |
| Gancao | | MOL004835 | Glypallichalcone | | | 61.6 | | 0.19 | |
| Gancao | | MOL004838 | 8-(6-hydroxy-2-benzofuranyl)-2,2-dimethyl-5-chromenol | | | 58.44 | | 0.38 | |
| Gancao | | MOL004841 | Licochalcone B | | | 76.76 | | 0.19 | |
| Gancao | | MOL004848 | licochalcone G | | | 49.25 | | 0.32 | |
| Gancao | | MOL004849 | 3-(2,4-dihydroxyphenyl)-8-(1,1-dimethylprop-2-enyl)-7-hydroxy-5-methoxy-coumarin | | | 59.62 | | 0.43 | |
| Gancao | | MOL004855 | Licoricone | | | 63.58 | | 0.47 | |
| Gancao | | MOL004856 | Gancaonin A | | | 51.08 | | 0.4 | |
| Gancao | | MOL004857 | Gancaonin B | | | 48.79 | | 0.45 | |
| Gancao | | MOL004860 | licorice glycoside E | | | 32.89 | | 0.27 | |
| Gancao | | MOL004863 | 3-(3,4-dihydroxyphenyl)-5,7-dihydroxy-8-(3-methylbut-2-enyl)chromone | | | 66.37 | | 0.41 | |
| Gancao | | MOL004864 | 5,7-dihydroxy-3-(4-methoxyphenyl)-8-(3-methylbut-2-enyl)chromone | | | 30.49 | | 0.41 | |
| Gancao | | MOL004866 | 2-(3,4-dihydroxyphenyl)-5,7-dihydroxy-6-(3-methylbut-2-enyl)chromone | | | 44.15 | | 0.41 | |
| Gancao | | MOL004879 | Glycyrin | | | 52.61 | | 0.47 | |
| Gancao | | MOL004882 | Licocoumarone | | | 33.21 | | 0.36 | |
| Gancao | | MOL004883 | Licoisoflavone | | | 41.61 | | 0.42 | |
| Gancao | | MOL004884 | Licoisoflavone B | | | 38.93 | | 0.55 | |
| Gancao | | MOL004885 | licoisoflavanone | | | 52.47 | | 0.54 | |
| Gancao | | MOL004891 | shinpterocarpin | | | 80.3 | | 0.73 | |
| Gancao | | MOL004898 | (E)-3-[3,4-dihydroxy-5-(3-methylbut-2-enyl)phenyl]-1-(2,4-dihydroxyphenyl)prop-2-en-1-one | | | 46.27 | | 0.31 | |
| Gancao | | MOL004903 | liquiritin | | | 65.69 | | 0.74 | |
| Gancao | | MOL004904 | licopyranocoumarin | | | 80.36 | | 0.65 | |
| Gancao | | MOL004905 | 3,22-Dihydroxy-11-oxo-delta(12)-oleanene-27-alpha-methoxycarbonyl-29-oic acid | | | 34.32 | | 0.55 | |
| Gancao | | MOL004907 | Glyzaglabrin | | | 61.07 | | 0.35 | |
| Gancao | | MOL004908 | Glabridin | | | 53.25 | | 0.47 | |
| Gancao | | MOL004910 | Glabranin | | | 52.9 | | 0.31 | |
| Gancao | | MOL004911 | Glabrene | | | 46.27 | | 0.44 | |
| Gancao | | MOL004912 | Glabrone | | | 52.51 | | 0.5 | |
| Gancao | | MOL004913 | 1,3-dihydroxy-9-methoxy-6-benzofurano[3,2-c]chromenone | | | 48.14 | | 0.43 | |
| Gancao | | MOL004914 | 1,3-dihydroxy-8,9-dimethoxy-6-benzofurano[3,2-c]chromenone | | | 62.9 | | 0.53 | |
| Gancao | | MOL004915 | Eurycarpin A | | | 43.28 | | 0.37 | |
| Gancao | | MOL004917 | glycyroside | | | 37.25 | | 0.79 | |
| Gancao | | MOL004924 | (-)-Medicocarpin | | | 40.99 | | 0.95 | |
| Gancao | | MOL004935 | Sigmoidin-B | | | 34.88 | | 0.41 | |
| Gancao | | MOL004941 | (2R)-7-hydroxy-2-(4-hydroxyphenyl)chroman-4-one | | | 71.12 | | 0.18 | |
| Gancao | | MOL004945 | (2S)-7-hydroxy-2-(4-hydroxyphenyl)-8-(3-methylbut-2-enyl)chroman-4-one | | | 36.57 | | 0.32 | |
| Gancao | | MOL004948 | Isoglycyrol | | | 44.7 | | 0.84 | |
| Gancao | | MOL004949 | Isolicoflavonol | | | 45.17 | | 0.42 | |
| Gancao | | MOL004957 | HMO | | | 38.37 | | 0.21 | |
| Gancao | | MOL004959 | 1-Methoxyphaseollidin | | | 69.98 | | 0.64 | |
| Gancao | | MOL004961 | Quercetin der. | | | 46.45 | | 0.33 | |
| Gancao | | MOL004966 | 3'-Hydroxy-4'-O-Methylglabridin | | | 43.71 | | 0.57 | |
| Gancao | | MOL000497 | licochalcone a | | | 40.79 | | 0.29 | |
| Gancao | | MOL004974 | 3'-Methoxyglabridin | | | 46.16 | | 0.57 | |
| Gancao | | MOL004978 | 2-[(3R)-8,8-dimethyl-3,4-dihydro-2H-pyrano[6,5-f]chromen-3-yl]-5-methoxyphenol | | | 36.21 | | 0.52 | |
| Gancao | | MOL004980 | Inflacoumarin A | | | 39.71 | | 0.33 | |
| Gancao | | MOL004985 | icos-5-enoic acid | | | 30.7 | | 0.2 | |
| Gancao | | MOL004988 | Kanzonol F | | | 32.47 | | 0.89 | |
| Gancao | | MOL004989 | 6-prenylated eriodictyol | | | 39.22 | | 0.41 | |
| Gancao | | MOL004990 | 7,2',4'-trihydroxy－5-methoxy-3－arylcoumarin | | | 83.71 | | 0.27 | |
| Gancao | | MOL004991 | 7-Acetoxy-2-methylisoflavone | | | 38.92 | | 0.26 | |
| Gancao | | MOL004993 | 8-prenylated eriodictyol | | | 53.79 | | 0.4 | |
| Gancao | | MOL004996 | gadelaidic acid | | | 30.7 | | 0.2 | |
| Gancao | | MOL000500 | Vestitol | | | 74.66 | | 0.21 | |
| Gancao | | MOL005000 | Gancaonin G | | | 60.44 | | 0.39 | |
| Gancao | | MOL005001 | Gancaonin H | | | 50.1 | | 0.78 | |
| Gancao | | MOL005003 | Licoagrocarpin | | | 58.81 | | 0.58 | |
| Gancao | | MOL005007 | Glyasperins M | | | 72.67 | | 0.59 | |
| Gancao | | MOL005008 | Glycyrrhiza flavonol A | | | 41.28 | | 0.6 | |
| Gancao | | MOL005012 | Licoagroisoflavone | | | 57.28 | | 0.49 | |
| Gancao | | MOL005013 | 18α-hydroxyglycyrrhetic acid | | | 41.16 | | 0.71 | |
| Gancao | | MOL005016 | Odoratin | | | 49.95 | | 0.3 | |
| Gancao | | MOL005017 | Phaseol | | | 78.77 | | 0.58 | |
| Gancao | | MOL005018 | Xambioona | | | 54.85 | | 0.87 | |
| Gancao | | MOL005020 | dehydroglyasperins C | | | 53.82 | | 0.37 | |
| Gancao | | MOL000098 | quercetin | | | 46.43 | | 0.28 | |
| Gua Louren | MOL002881 | | | Diosmetin | 31.14 | | 0.27 | |  |
| Gua Louren | MOL000449 | | | Stigmasterol | 43.83 | | 0.76 | |  |
| Gua Louren | MOL003044 | | | Chryseriol | 35.85 | | 0.27 | |  |
