# Supplementary material for: Study the Mechanism of Gualou Niubang Decoction in Treating Plasma Cell Mastitis Based on Network Pharmacology and Molecular Docking
Source: Biomed Res Int. 2022 Jun 15;2022:5780936. doi: 10.1155/2022/5780936 (PMC9217541; doi:10.1155/2022/5780936)
Supplement: Supplementary Materials — S1: 240 active components of Trichosanthis Niubang decoction (including repeated values). S2: PubChem CID information of 151 active components of Trichosanthes Niubang decoction (excluding duplication). S3: Venn diagram of intersection of drugs and diseases. S4: component-ingredient-disease-target gene network data. S5: G0 enrichment analysis (35 cell compositions). S6: G0 enrichment analysis (242 biological processes). S7: G0 enrichment analysis (59 molecular functions). S8: 200 KEGG pathway enrichment analyses. [file 5780936.f1.zip › Table S2 Ingredients after screening.docx]

S 2 Pubchem CID information of 151 active components of Trichosanthes Niubang Decoction (excluding duplication).

| Herb | MOLname | Compounds | OB | DL | PubChem CID |
| --- | --- | --- | --- | --- | --- |
| Niu Bangzi  Niu Bangzi | MOL010868 | neoarctin A | 39.99 | 0.27 | 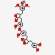 |
|  |  |  |  |  |  |
| Niu Bangzi  Niu Bangzi | MOL000522 | arctiin | 34.45 | 0.84 | 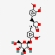 |
|  |  |  |  |  |  |
| Niu Bangzi  Niu Bangzi | MOL000358 | beta-sitosterol | 36.91 | 0.75 | 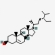 |
|  |  |  |  |  |  |
| Niu Bangzi  Niu Bangzi | MOL000422 | kaempferol | 41.88 | 0.24 | 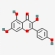 |
|  |  |  |  |  |  |
| Niu Bangzi  Niu Bangzi | MOL001506 | Supraene | 33.55 | 0.42 | 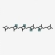 |
|  |  |  |  |  |  |
| Niu Bangzi  Niu Bangzi | MOL002773 | beta-carotene | 37.18 | 0.58 | 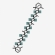 |
|  |  |  |  |  |  |
| Niu Bangzi  Niu Bangzi | MOL003290 | (3R,4R)-3,4-bis[(3,4-dimethoxyphenyl)methyl]oxolan-2-one | 52.3 | 0.48 | 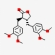 |
|  |  |  |  |  |  |
| Niu Bangzi | MOL007326 | Cynarin(e) | 31.76 | 0.68 | 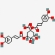 |
|  |  |  |  |  |  |
| Tian Huafen  Tian Huafen | MOL004355 | Spinasterol | 42.98 | 0.76 | 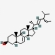 |
|  |  |  |  |  |  |
| Tian Huafen | MOL006756 | Schottenol | 37.42 | 0.75 | 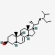 |
|  |  |  |  |  |  |
| Zhi zi  Zhi zi | MOL001406 | crocetin | 35.3 | 0.26 | 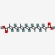 |
|  |  |  |  |  |  |
| Zhi zi | MOL001941 | Ammidin | 34.55 | 0.22 |  |
| Zhi zi  Zhi zi | MOL004561 | Sudan III | 84.07 | 0.59 | 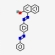 |
|  |  |  |  |  |  |
| Zhi zi  Zhi zi | MOL000098 | quercetin | 46.43 | 0.28 | 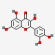 |
|  |  |  |  |  |  |
| Zhi zi  Zhi zi | MOL000449 | Stigmasterol | 43.83 | 0.76 | 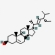 |
|  |  |  |  |  |  |
| Zhi zi  Zhi zi | MOL001494 | Mandenol | 42 | 0.19 | ~~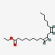~~ |
|  |  |  |  |  |  |
| Zhi zi  Zhi zi | MOL001506 | Supraene | 33.55 | 0.42 | 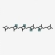 |
|  |  |  |  |  |  |
| Zhi zi  Zhi zi | MOL001942 | isoimperatorin | 45.46 | 0.23 | 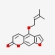 |
|  |  |  |  |  |  |
| Zhi zi  Zhi zi | MOL002883 | Ethyl oleate (NF) | 32.4 | 0.19 | 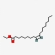 |
|  |  |  |  |  |  |
| Zhi zi  Zhi zi | MOL007245 | 3-Methylkempferol | 60.16 | 0.26 | 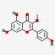 |
|  |  |  |  |  |  |
| Zhi zi | MOL009038 | GBGB | 45.58 | 0.83 | 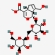 |
|  |  |  |  |  |  |
| Zao Jiaoci  Zao Jiaoci | MOL013179 | fisetin | 52.6 | 0.24 | 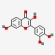 |
|  |  |  |  |  |  |
| Zao Jiaoci  Zao Jiaoci | MOL013296 | Fustin | 50.91 | 0.24 | 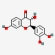 |
|  |  |  |  |  |  |
| Zao Jiaoci  Zao Jiaoci | MOL001736 | (-)-taxifolin | 60.51 | 0.27 | 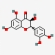 |
|  |  |  |  |  |  |
| Zao Jiaoci  Zao Jiaoci | MOL002914 | Eriodyctiol (flavanone) | 41.35 | 0.24 | 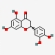 |
|  |  |  |  |  |  |
| Zao Jiaoci | MOL000358 | beta-sitosterol | 36.91 | 0.75 |  |
| Zao Jiaoci  Zao Jiaoci | MOL000359 | sitosterol | 36.91 | 0.75 | 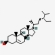 |
|  |  |  |  |  |  |
| Zao Jiaoci  Zao Jiaoci | MOL006358 | Stigmast-4-ene-3,6-dione | 39.12 | 0.79 | 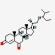 |
|  |  |  |  |  |  |
| Zao Jiaoci | MOL000073 | ent-Epicatechin | 48.96 | 0.24 | 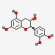 |
|  |  |  |  |  |  |
| Qingpi | MOL001803 | Sinensetin | 50.56 | 0.45 | 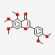 |
|  |  |  |  |  |  |
| Qingpi  Qingpi | MOL004328 | naringenin | 59.29 | 0.21 | 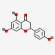 |
|  |  |  |  |  |  |
| Qingpi | MOL005100 | 5,7-dihydroxy-2-(3-hydroxy-4-methoxyphenyl)chroman-4-one | 47.74 | 0.27 | 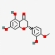 |
|  |  |  |  |  |  |
| Qingpi | MOL005828 | nobiletin | 61.67 | 0.52 | 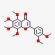 |
|  |  |  |  |  |  |
| Chaihu | MOL001645 | Linoleyl acetate | 42.1 | 0.2 | 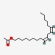 |
|  |  |  |  |  |  |
| Chaihu  Chaihu | MOL002776 | Baicalin | 40.12 | 0.75 | 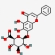 |
|  |  |  |  |  |  |
| Chaihu | MOL000449 | Stigmasterol | 43.83 | 0.76 |  |
| Chaihu  Chaihu | MOL000354 | isorhamnetin | 49.6 | 0.31 | 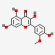 |
|  |  |  |  |  |  |
| Chaihu  Chaihu | MOL013187 | Cubebin | 57.13 | 0.64 | 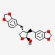 |
|  |  |  |  |  |  |
| Chaihu  Chaihu | MOL004624 | Longikaurin A | 47.72 | 0.53 | 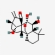 |
|  |  |  |  |  |  |
| Chaihu  Chaihu | MOL004628 | Octalupine | 47.82 | 0.28 | 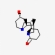 |
|  |  |  |  |  |  |
| Chaihu  Chaihu | MOL004644 | Sainfuran | 79.91 | 0.23 | 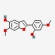 |
|  |  |  |  |  |  |
| Chaihu  Chaihu | MOL004648 | Troxerutin | 31.6 | 0.28 | 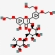 |
|  |  |  |  |  |  |
| Chaihu  Chaihu | MOL004653 | (+)-Anomalin | 46.06 | 0.66 | 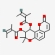 |
|  |  |  |  |  |  |
| Chaihu  Chaihu | MOL004718 | α-spinasterol | 42.98 | 0.76 | 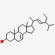 |
|  |  |  |  |  |  |
| Chaihu  Chaihu | MOL000490 | petunidin | 30.05 | 0.31 | 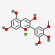 |
|  |  |  |  |  |  |
| Chaihu  Chaihu | MOL001689 | acacetin | 34.97 | 0.24 | 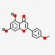 |
|  |  |  |  |  |  |
| Chaihu  Chaihu | MOL000173 | wogonin | 30.68 | 0.23 | 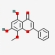 |
|  |  |  |  |  |  |
| Chaihu  Chaihu | MOL002714 | baicalein | 33.52 | 0.21 | 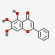 |
|  |  |  |  |  |  |
| Chaihu  Chaihu | MOL002908 | 5,8,2'-Trihydroxy-7-methoxyflavone | 37.01 | 0.27 | 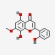 |
|  |  |  |  |  |  |
| Chaihu  Chaihu | MOL002910 | Carthamidin | 41.15 | 0.24 | 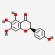 |
|  |  |  |  |  |  |
| Chaihu | MOL002913 | Dihydrobaicalin_qt | 40.04 | 0.21 |  |
| Chaihu  Chaihu | MOL002914 | Eriodyctiol (flavanone) | 41.35 | 0.24 | 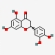 |
|  |  |  |  |  |  |
| Chaihu  Chaihu | MOL002915 | Salvigenin | 49.07 | 0.33 | 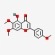 |
|  |  |  |  |  |  |
| Chaihu  Chaihu | MOL002925 | 5,7,2',6'-Tetrahydroxyflavone | 37.01 | 0.24 | 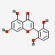 |
|  |  |  |  |  |  |
| Chaihu  Chaihu | MOL002926 | dihydrooroxylin A | 38.72 | 0.23 | 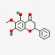 |
|  |  |  |  |  |  |
| Chaihu  Chaihu | MOL002927 | Skullcapflavone II | 69.51 | 0.44 | 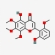 |
|  |  |  |  |  |  |
| Chaihu  Chaihu | MOL002928 | oroxylin a | 41.37 | 0.23 | 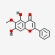 |
|  |  |  |  |  |  |
| Chaihu | MOL002932 | Panicolin | 76.26 | 0.29 |  |
| Chaihu  Chaihu | MOL002933 | 5,7,4'-Trihydroxy-8-methoxyflavone | 36.56 | 0.27 | 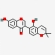 |
|  |  |  |  |  |  |
| Chaihu  Chaihu | MOL002934 | NEOBAICALEIN | 104.34 | 0.44 | 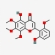 |
|  |  |  |  |  |  |
| Chaihu  Chaihu | MOL002937 | DIHYDROOROXYLIN | 66.06 | 0.23 | 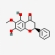 |
|  |  |  |  |  |  |
| Chaihu  Chaihu | MOL000525 | Norwogonin | 39.4 | 0.21 | 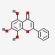 |
|  |  |  |  |  |  |
| Chaihu  Chaihu | MOL000552 | 5,2'-Dihydroxy-6,7,8-trimethoxyflavone | 31.71 | 0.35 | 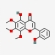 |
|  |  |  |  |  |  |
| Chaihu  Chaihu | MOL000073 | ent-Epicatechin | 48.96 | 0.24 | 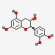 |
|  |  |  |  |  |  |
| Chaihu  Chaihu | MOL001458 | coptisine | 30.67 | 0.86 | 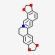 |
|  |  |  |  |  |  |
| Chaihu  Chaihu | MOL001490 | bis[(2S)-2-ethylhexyl] benzene-1,2-dicarboxylate | 43.59 | 0.35 | 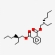 |
|  |  |  |  |  |  |
| Chaihu  Chaihu | MOL002897 | epiberberine | 43.09 | 0.78 | 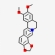 |
|  |  |  |  |  |  |
| Chaihu  Chaihu | MOL008206 | Moslosooflavone | 44.09 | 0.25 | 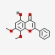 |
|  |  |  |  |  |  |
| Chaihu  Chaihu | MOL010415 | 11,13-Eicosadienoic acid, methyl ester | 39.28 | 0.23 | 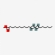 |
|  |  |  |  |  |  |
| Chaihu  Chaihu | MOL012245 | 5,7,4'-trihydroxy-6-methoxyflavanone | 36.63 | 0.27 | 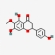 |
|  |  |  |  |  |  |
| Chaihu  Chaihu | MOL012246 | 5,7,4'-trihydroxy-8-methoxyflavanone | 74.24 | 0.26 | 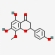 |
|  |  |  |  |  |  |
| Chaihu | MOL012266 | rivularin | 37.94 | 0.37 | 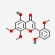 |
|  |  |  |  |  |  |
| Lonicerae Japonicae Flos | MOL001494 | Mandenol | 42 | 0.19 |  |
| Jin Yinhua | MOL001495 | Ethyl linolenate | 46.1 | 0.2 | 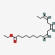 |
|  |  |  |  |  |  |
| Jin Yinhua  Jin Yinhua | MOL002707 | phytofluene | 43.18 | 0.5 | 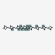 |
|  |  |  |  |  |  |
| Jin Yinhua  Jin Yinhua | MOL002914 | Eriodyctiol (flavanone) | 41.35 | 0.24 | 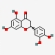 |
|  |  |  |  |  |  |
| Jin Yinhua  Jin Yinhua | MOL002773 | beta-carotene | 37.18 | 0.58 | 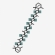 |
|  |  |  |  |  |  |
| Jin Yinhua  Jin Yinhua | MOL003044 | Chryseriol | 35.85 | 0.27 | 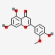 |
|  |  |  |  |  |  |
| Jin Yinhua  Jin Yinhua | MOL003059 | kryptoxanthin | 47.25 | 0.57 | 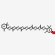 |
|  |  |  |  |  |  |
| Jin Yinhua  Jin Yinhua | MOL003062 | 4,5'-Retro-.beta.,.beta.-Carotene-3,3'-dione, 4',5'-didehydro- | 31.22 | 0.55 | 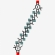 |
|  |  |  |  |  |  |
| Jin Yinhua | MOL003095 | 5-hydroxy-7-methoxy-2-(3,4,5-trimethoxyphenyl)chromone | 51.96 | 0.41 |  |
| Jin Yinhua  Jin Yinhua | MOL003101 | 7-epi-Vogeloside | 46.13 | 0.58 | 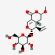 |
|  |  |  |  |  |  |
| Jin Yinhua | MOL003108 | Caeruloside C | 55.64 | 0.73 |  |
| Jin Yinhua | MOL003111 | Centauroside_qt | 55.79 | 0.5 |  |
| Jin Yinhua | MOL003117 | Ioniceracetalides B_qt | 61.19 | 0.19 |  |
| Jin Yinhua | MOL003124 | XYLOSTOSIDINE | 43.17 | 0.64 | 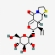 |
|  |  |  |  |  |  |
| Lianqiao | MOL003315 | 3beta-Acetyl-20,25-epoxydammarane-24alpha-ol | 33.07 | 0.79 | 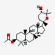 |
|  |  |  |  |  |  |
| Lianqiao  Lianqiao | MOL000211 | Mairin | 55.38 | 0.78 | 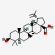 |
|  |  |  |  |  |  |
| Lianqiao  Lianqiao | MOL003322 | FORSYTHINOL | 81.25 | 0.57 | 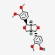 |
|  |  |  |  |  |  |
| Lianqiao | MOL003330 | (-)-Phillygenin | 95.04 | 0.57 |  |
| Lianqiao  Lianqiao | MOL003344 | β-amyrin acetate | 42.06 | 0.74 | 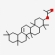 |
|  |  |  |  |  |  |
| Lianqiao  Lianqiao | MOL003347 | hyperforin | 44.03 | 0.6 | 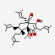 |
|  |  |  |  |  |  |
| Lianqiao  Lianqiao | MOL003348 | adhyperforin | 44.03 | 0.61 | 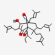 |
|  |  |  |  |  |  |
| Lianqiao  Lianqiao | MOL003365 | Lactucasterol | 40.99 | 0.85 | 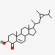 |
|  |  |  |  |  |  |
| Lianqiao  Lianqiao | MOL003370 | Onjixanthone I | 79.16 | 0.3 | 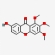 |
|  |  |  |  |  |  |
| Lianqiao | MOL000791 | bicuculline | 69.67 | 0.88 | 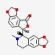 |
|  |  |  |  |  |  |
| Chenpi | MOL000359 | sitosterol | 36.91 | 0.75 | 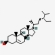 |
|  |  |  |  |  |  |
| Chenpi  Chenpi | MOL004328 | naringenin | 59.29 | 0.21 | 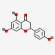 |
|  |  |  |  |  |  |
| Chenpi  Chenpi | MOL005100 | 5,7-dihydroxy-2-(3-hydroxy-4-methoxyphenyl)chroman-4-one | 47.74 | 0.27 | 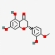 |
|  |  |  |  |  |  |
| Chenpi  Chenpi | MOL005815 | Citromitin | 86.9 | 0.51 | 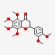 |
|  |  |  |  |  |  |
| Chenpi | MOL005828 | nobiletin | 61.67 | 0.52 | 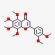 |
|  |  |  |  |  |  |
| Gancao | MOL001484 | Inermine | 75.18 | 0.54 | 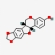 |
|  |  |  |  |  |  |
| Gancao  Gancao | MOL001792 | DFV | 32.76 | 0.18 | 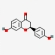 |
|  |  |  |  |  |  |
| Gancao  Gancao | MOL002311 | Glycyrol | 90.78 | 0.67 | 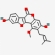 |
|  |  |  |  |  |  |
| Gancao  Gancao | MOL000239 | Jaranol | 50.83 | 0.29 | 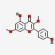 |
|  |  |  |  |  |  |
| Gancao  Gancao | MOL002565 | Medicarpin | 49.22 | 0.34 | 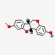 |
|  |  |  |  |  |  |
| Gancao  Gancao | MOL000354 | isorhamnetin | 49.6 | 0.31 | 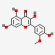 |
|  |  |  |  |  |  |
| Gancao  Gancao | MOL003656 | Lupiwighteone | 51.64 | 0.37 | 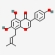 |
|  |  |  |  |  |  |
| Gancao  Gancao | MOL003896 | 7-Methoxy-2-methyl isoflavone | 42.56 | 0.2 | 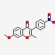 |
|  |  |  |  |  |  |
| Gancao  Gancao | MOL000392 | formononetin | 69.67 | 0.21 | 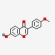 |
|  |  |  |  |  |  |
| Gancao  Gancao | MOL000417 | Calycosin | 47.75 | 0.24 | 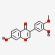 |
|  |  |  |  |  |  |
| Gancao  Gancao | MOL004328 | naringenin | 59.29 | 0.21 |  |
|  |  |  |  |  |  |
| Gancao  Gancao | MOL004805 | (2S)-2-[4-hydroxy-3-(3-methylbut-2-enyl)phenyl]-8,8-dimethyl-2,3-dihydropyrano[2,3-f]chromen-4-one | 31.79 | 0.72 |  |
|  |  |  |  |  |  |
| Gancao | MOL004806 | euchrenone | 30.29 | 0.57 |  |
| Gancao  Gancao | MOL004808 | glyasperin B | 65.22 | 0.44 |  |
|  |  |  |  |  |  |
| Gancao  Gancao | MOL004810 | glyasperin F | 75.84 | 0.54 |  |
|  |  |  |  |  |  |
| Gancao  Gancao | MOL004811 | Glyasperin C | 45.56 | 0.4 |  |
|  |  |  |  |  |  |
| Gancao  Gancao | MOL004814 | Isotrifoliol | 31.94 | 0.42 |  |
|  |  |  |  |  |  |
| Gancao  Gancao | MOL004815 | (E)-1-(2,4-dihydroxyphenyl)-3-(2,2-dimethylchromen-6-yl)prop-2-en-1-one | 39.62 | 0.35 |  |
|  |  |  |  |  |  |
| Gancao | MOL004820 | kanzonols W | 50.48 | 0.52 |  |
| Gancao  Gancao | MOL004824 | (2S)-6-(2,4-dihydroxyphenyl)-2-(2-hydroxypropan-2-yl)-4-methoxy-2,3-dihydrofuro[3,2-g]chromen-7-one | 60.25 | 0.63 |  |
|  |  |  |  |  |  |
| Gancao  Gancao | MOL004827 | Semilicoisoflavone B | 48.78 | 0.55 |  |
|  |  |  |  |  |  |
| Gancao  Gancao | MOL004828 | Glepidotin A | 44.72 | 0.35 |  |
|  |  |  |  |  |  |
| Gancao  Gancao | MOL004829 | Glepidotin B | 64.46 | 0.34 |  |
|  |  |  |  |  |  |
| Gancao  Gancao | MOL004833 | Phaseolinisoflavan | 32.01 | 0.45 |  |
|  |  |  |  |  |  |
| Gancao  Gancao | MOL004835 | Glypallichalcone | 61.6 | 0.19 |  |
|  |  |  |  |  |  |
| Gancao  Gancao | MOL004841 | Licochalcone B | 76.76 | 0.19 |  |
|  |  |  |  |  |  |
| Gancao  Gancao | MOL004848 | licochalcone G | 49.25 | 0.32 |  |
|  |  |  |  |  |  |
| Gancao  Gancao | MOL004856 | Gancaonin A | 51.08 | 0.4 |  |
|  |  |  |  |  |  |
| Gancao  Gancao | MOL004857 | Gancaonin B | 48.79 | 0.45 |  |
|  |  |  |  |  |  |
| Gancao  Gancao | MOL004860 | licorice glycoside E | 32.89 | 0.27 |  |
|  |  |  |  |  |  |
| Gancao  Gancao | MOL004879 | Glycyrin | 52.61 | 0.47 |  |
|  |  |  |  |  |  |
| Gancao  Gancao | MOL004882 | Licocoumarone | 33.21 | 0.36 |  |
|  |  |  |  |  |  |
| Gancao  Gancao | MOL004883 | Licoisoflavone | 41.61 | 0.42 |  |
|  |  |  |  |  |  |
| Gancao  Gancao | MOL004884 | Licoisoflavone B | 38.93 | 0.55 |  |
|  |  |  |  |  |  |
| Gancao  Gancao | MOL004885 | licoisoflavanone | 52.47 | 0.54 |  |
|  |  |  |  |  |  |
| Gancao  Gancao | MOL004891 | shinpterocarpin | 80.3 | 0.73 |  |
|  |  |  |  |  |  |
| Gancao  Gancao | MOL004898 | (E)-3-[3,4-dihydroxy-5-(3-methylbut-2-enyl)phenyl]-1-(2,4-dihydroxyphenyl)prop-2-en-1-one | 46.27 | 0.31 |  |
|  |  |  |  |  |  |
| Gancao  Gancao | MOL004903 | liquiritin | 65.69 | 0.74 |  |
|  |  |  |  |  |  |
| Gancao  Gancao | MOL004904 | licopyranocoumarin | 80.36 | 0.65 |  |
|  |  |  |  |  |  |
| Gancao  Gancao | MOL004905 | 3,22-Dihydroxy-11-oxo-delta(12)-oleanene-27-alpha-methoxycarbonyl-29-oic acid | 34.32 | 0.55 |  |
|  |  |  |  |  |  |
| Gancao  Gancao | MOL004907 | Glyzaglabrin | 61.07 | 0.35 |  |
|  |  |  |  |  |  |
| Gancao  Gancao | MOL004908 | Glabridin | 53.25 | 0.47 |  |
|  |  |  |  |  |  |
| Gancao  Gancao | MOL004910 | Glabranin | 52.9 | 0.31 |  |
|  |  |  |  |  |  |
| Gancao  Gancao | MOL004911 | Glabrene | 46.27 | 0.44 |  |
|  |  |  |  |  |  |
| Gancao  Gancao | MOL004912 | Glabrone | 52.51 | 0.5 |  |
|  |  |  |  |  |  |
| Gancao  Gancao | MOL004915 | Eurycarpin A | 43.28 | 0.37 |  |
|  |  |  |  |  |  |
| Gancao  Gancao | MOL004917 | glycyroside | 37.25 | 0.79 |  |
|  |  |  |  |  |  |
| Gancao  Gancao | MOL004924 | (-)-Medicocarpin | 40.99 | 0.95 |  |
|  |  |  |  |  |  |
| Gancao  Gancao | MOL004935 | Sigmoidin-B | 34.88 | 0.41 |  |
|  |  |  |  |  |  |
| Gancao  Gancao | MOL004948 | Isoglycyrol | 44.7 | 0.84 |  |
|  |  |  |  |  |  |
| Gancao  Gancao | MOL004949 | Isolicoflavonol | 45.17 | 0.42 |  |
|  |  |  |  |  |  |
| Gancao | MOL004957 | HMO | 38.37 | 0.21 |  |
| Gancao  Gancao | MOL004959 | 1-Methoxyphaseollidin | 69.98 | 0.64 |  |
|  |  |  |  |  |  |
| Gancao  Gancao | MOL004961 | Quercetin der. | 46.45 | 0.33 |  |
|  |  |  |  |  |  |
| Gancao  Gancao | MOL004966 | 3'-Hydroxy-4'-O-Methylglabridin | 43.71 | 0.57 |  |
|  |  |  |  |  |  |
| Gancao  Gancao | MOL000497 | licochalcone a | 40.79 | 0.29 |  |
|  |  |  |  |  |  |
| Gancao  Gancao | MOL004974 | 3'-Methoxyglabridin | 46.16 | 0.57 |  |
|  |  |  |  |  |  |
| Gancao  Gancao | MOL004980 | Inflacoumarin A | 39.71 | 0.33 |  |
|  |  |  |  |  |  |
| Gancao  Gancao | MOL004985 | icos-5-enoic acid | 30.7 | 0.2 |  |
|  |  |  |  |  |  |
| Gancao  Gancao | MOL004988 | Kanzonol F | 32.47 | 0.89 |  |
|  |  |  |  |  |  |
| Gancao  Gancao | MOL004990 | 7,2',4'-trihydroxy－5-methoxy-3－arylcoumarin | 83.71 | 0.27 |  |
|  |  |  |  |  |  |
| Gancao  Gancao | MOL004991 | 7-Acetoxy-2-methylisoflavone | 38.92 | 0.26 |  |
|  |  |  |  |  |  |
| Gancao  Gancao | MOL004996 | gadelaidic acid | 30.7 | 0.2 |  |
|  |  |  |  |  |  |
| Gancao  Gancao | MOL000500 | Vestitol | 74.66 | 0.21 |  |
|  |  |  |  |  |  |
| Gancao  Gancao | MOL005000 | Gancaonin G | 60.44 | 0.39 |  |
|  |  |  |  |  |  |
| Gancao  Gancao | MOL005001 | Gancaonin H | 50.1 | 0.78 |  |
|  |  |  |  |  |  |
| Gancao  Gancao | MOL005003 | Licoagrocarpin | 58.81 | 0.58 |  |
|  |  |  |  |  |  |
| Gancao  Gancao | MOL005008 | Glycyrrhiza flavonol A | 41.28 | 0.6 |  |
|  |  |  |  |  |  |
| Gancao  Gancao | MOL005012 | Licoagroisoflavone | 57.28 | 0.49 |  |
|  |  |  |  |  |  |
| Gancao  Gancao | MOL005013 | 18α-hydroxyglycyrrhetic acid | 41.16 | 0.71 |  |
|  |  |  |  |  |  |
| Gancao  Gancao | MOL005016 | Odoratin | 49.95 | 0.3 |  |
|  |  |  |  |  |  |
| Gancao  Gancao | MOL005017 | Phaseol | 78.77 | 0.58 |  |
|  |  |  |  |  |  |
| Gancao  Gancao | MOL005018 | Xambioona | 54.85 | 0.87 |  |
|  |  |  |  |  |  |
| Gancao  Gancao | MOL002881 | Diosmetin | 31.14 | 0.27 |  |
|  |  |  |  |  |  |
| Gancao  Gancao | MOL000449 | Stigmasterol | 43.83 | 0.76 |  |
|  |  |  |  |  |  |
| Gancao | MOL003044 | Chryseriol | 35.85 | 0.27 |  |
