# Supplementary material for: Study the Mechanism of Gualou Niubang Decoction in Treating Plasma Cell Mastitis Based on Network Pharmacology and Molecular Docking
Source: Biomed Res Int. 2022 Jun 15;2022:5780936. doi: 10.1155/2022/5780936 (PMC9217541; doi:10.1155/2022/5780936)
Supplement: Supplementary Materials — S1: 240 active components of Trichosanthis Niubang decoction (including repeated values). S2: PubChem CID information of 151 active components of Trichosanthes Niubang decoction (excluding duplication). S3: Venn diagram of intersection of drugs and diseases. S4: component-ingredient-disease-target gene network data. S5: G0 enrichment analysis (35 cell compositions). S6: G0 enrichment analysis (242 biological processes). S7: G0 enrichment analysis (59 molecular functions). S8: 200 KEGG pathway enrichment analyses. [file 5780936.f1.zip › Table S3 venn.docx]

S3 Veen diagram of intersection of drugs and diseases

MUC1

PGR

CHRM3

BCL2

CASP9

CASP3

PON1

PPARG

PRSS1

CYP1A1

ICAM1

SELE

VCAM1

AHR

VEGFA

ALB

MYC

ESR1

EGFR

FOS

RB1

IL6

CDKN2A

TP63

POR

RAF1

HIF1A

ERBB2

COL3A1

CRP

CTSD

IGF2

IRF1

RASA1

PCNA

MCL1

CDC25C

CD36

TCF7

LDLR

APOB

TIMP1

CYP2C9

NOS3

GBA

CYP19A1

CXCL8

MMP1

MMP2

MMP3

MMP9

PLG

INSR

BCL2L1

TOP1

MAPK1

TLR2

SHBG
