# Supplementary material for: Study the Mechanism of Gualou Niubang Decoction in Treating Plasma Cell Mastitis Based on Network Pharmacology and Molecular Docking
Source: Biomed Res Int. 2022 Jun 15;2022:5780936. doi: 10.1155/2022/5780936 (PMC9217541; doi:10.1155/2022/5780936)
Supplement: Supplementary Materials — S1: 240 active components of Trichosanthis Niubang decoction (including repeated values). S2: PubChem CID information of 151 active components of Trichosanthes Niubang decoction (excluding duplication). S3: Venn diagram of intersection of drugs and diseases. S4: component-ingredient-disease-target gene network data. S5: G0 enrichment analysis (35 cell compositions). S6: G0 enrichment analysis (242 biological processes). S7: G0 enrichment analysis (59 molecular functions). S8: 200 KEGG pathway enrichment analyses. [file 5780936.f1.zip › Table S4 Network construction.docx]

S4 component-ingredient- disease- target gene network data

| IS | MOL000354 | CH | ESR1 |
| --- | --- | --- | --- |
| IS | MOL000354 | CH | MMP9 |
| IS | MOL000354 | CH | MUC1 |
| IS | MOL000354 | CH | EGFR |
| IS | MOL000354 | CH | PCNA |
| IS | MOL000354 | CH | PPARG |
| IS | MOL000354 | CH | PRSS1 |
| IS | MOL000354 | CH | IL6 |
| IS | MOL000354 | CH | AHR |
| K | MOL000422 | CH | BCL2 |
| K | MOL000422 | CH | CASP3 |
| K | MOL000422 | CH | CYP1A1 |
| K | MOL000422 | CH | ICAM1 |
| K | MOL000422 | CH | MAPK1 |
| K | MOL000422 | CH | MMP9 |
| K | MOL000422 | CH | PGR |
| K | MOL000422 | CH | PPARG |
| K | MOL000422 | CH | PRSS1 |
| K | MOL000422 | CH | IL6 |
| K | MOL000422 | CH | RB1 |
| K | MOL000422 | CH | ESR1 |
| K | MOL000422 | CH | VEGFA |
| K | MOL000422 | CH | AHR |
| K | MOL000422 | CH | BCL2L1 |
| Q | MOL000098 | CH | CASP3 |
| Q | MOL000098 | CH | CASP9 |
| Q | MOL000098 | CH | CDKN2A |
| Q | MOL000098 | CH | COL3A1 |
| Q | MOL000098 | CH | CRP |
| Q | MOL000098 | CH | CTSD |
| Q | MOL000098 | CH | CYP1A1 |
| Q | MOL000098 | CH | EGFR |
| Q | MOL000098 | CH | ERBB2 |
| Q | MOL000098 | CH | FOS |
| Q | MOL000098 | CH | HIF1A |
| Q | MOL000098 | CH | ICAM1 |
| Q | MOL000098 | CH | IGF2 |
| Q | MOL000098 | CH | IL6 |
| Q | MOL000098 | CH | IRF1 |
| Q | MOL000098 | CH | MMP1 |
| Q | MOL000098 | CH | MYC |
| Q | MOL000098 | CH | NOS3 |
| Q | MOL000098 | CH | ALB |
| Q | MOL000098 | CH | NOS7 |
| Q | MOL000098 | CH | NOS16 |
| Q | MOL000098 | CH | PGR |
| Q | MOL000098 | CH | POR |
| Q | MOL000098 | CH | PPARG |
| Q | MOL000098 | CH | PRSS1 |
| Q | MOL000098 | CH | RAF1 |
| Q | MOL000098 | CH | RB1 |
| Q | MOL000098 | CH | TOP1 |
| Q | MOL000098 | CH | TLR2 |
| Q | MOL000098 | CH | VEGFA |
| Q | MOL000098 | CH | CHRM3 |
| Q | MOL000098 | CH | AHR |
| Q | MOL000098 | CH | BCL2 |
| Q | MOL000098 | CH | BCL2L1 |
| Q | MOL000098 | CH | EPHB2 |
| ST | MOL000449 | CH | ESR1 |
|  | MOL000422 | CH | ESR1 |
|  | MOL000098 | CH | IGF2 |
|  | MOL000422 | CH | IGF2 |
|  | MOL000422 | CH | MUC1 |
|  | MOL000098 | CH | IL6 |
|  | MOL000098 | CH | PCNA |
|  | MOL000449 | CH | NOS3 |
|  | MOL000098 | CH | NOS3 |
|  | MOL000098 | CH | ALB |
|  | MOL000422 | CH | ALB |
|  | MOL000098 | CH | EGFR |
|  | MOL000098 | CH | CASP3 |
|  | MOL000354 | CH | CASP3 |
|  | MOL004598 | CH | CASP3 |
|  | MOL000098 | CH | PGR |
|  | MOL000422 | CH | PGR |
|  | MOL000449 | CH | PGR |
|  | MOL000354 | CH | PGR |
|  | MOL000422 | CH | PPARG |
|  | MOL000098 | CH | PPARG |
|  | MOL000098 | CH | PRSS1 |
|  | MOL000422 | CH | PRSS1 |
|  | MOL000449 | CH | PRSS1 |
|  | MOL000354 | CH | PRSS1 |
|  | MOL004609 | CH | PRSS1 |
|  | MOL000449 | CH | RAF1 |
|  | MOL000098 | CH | RAF1 |
| NA | MOL004328 | CP | CASP9 |
| NA | MOL004328 | CP | ICAM1 |
| NA | MOL004328 | CP | MMP1 |
| NA | MOL004328 | CP | TIMP1 |
| NA | MOL004328 | CP | PRSS1 |
| NA | MOL004328 | CP | VEGFA |
| NA | MOL004328 | CP | ESR1 |
| NA | MOL004328 | CP | APOB |
| NA | MOL004328 | CP | BCL2 |
| NA | MOL004328 | CP | CASP3 |
| NO | MOL005828 | CP | LDLR |
| NO | MOL005828 | CP | MMP2 |
| NO | MOL005828 | CP | PON1 |
| NO | MOL005828 | CP | PPARG |
| NO | MOL005828 | CP | EGFR |
| NO | MOL005828 | CP | ALB |
| NO | MOL005828 | CP | TLR2 |
| NO | MOL005828 | CP | VCAM1 |
| NO | MOL005828 | CP | MYC |
| SI | MOL000359 | CP | ESR1 |
| SI | MOL000359 | CP | CASP3 |
| SI | MOL000359 | CP | CASP9 |
|  | MOL004328 | CP | CASP9 |
|  | MOL004328 | CP | ESR1 |
|  | MOL005828 | CP | ESR1 |
|  | MOL005828 | CP | MAPK1 |
|  | MOL004328 | CP | MAPK1 |
|  | MOL005828 | CP | NOS3 |
|  | MOL004328 | CP | IL6 |
|  | MOL005828 | CP | EGFR |
|  | MOL004328 | CP | ALB |
|  | MOL000359 | CP | ALB |
|  | MOL005815 | CP | PGR |
|  | MOL005828 | CP | PPARG |
|  | MOL000359 | CP | PRSS1 |
|  | MOL004328 | CP | PRSS1 |
| IS | MOL000354 | GC | ESR1 |
| IS | MOL000354 | GC | NOS3 |
| IS | MOL000354 | GC | HIF1A |
| IS | MOL000354 | GC | PPARG |
| IS | MOL000354 | GC | PRSS1 |
| IS | MOL000354 | GC | AHR |
| IS | MOL000354 | GC | BCL2 |
| IS | MOL000354 | GC | CASP3 |
| K | MOL000422 | GC | CYP1A1 |
| K | MOL000422 | GC | ICAM1 |
| K | MOL000422 | GC | MAPK1 |
| K | MOL000422 | GC | NOS3 |
| K | MOL000422 | GC | FOS |
| K | MOL000422 | GC | PGR |
| K | MOL000422 | GC | PPARG |
| K | MOL000422 | GC | PRSS1 |
| K | MOL000422 | GC | RAF1 |
| K | MOL000422 | GC | ALB |
| K | MOL000422 | GC | VEGFA |
| K | MOL000422 | GC | EGFR |
| K | MOL000422 | GC | IL6 |
| K | MOL000422 | GC | ESR1 |
| K | MOL000422 | GC | TP63 |
| K | MOL000422 | GC | APOB |
| MA | MOL000211 | GC | BCL2 |
| NA | MOL004328 | GC | CASP3 |
| NA | MOL004328 | GC | ESR1 |
| NA | MOL004328 | GC | CASP9 |
| NA | MOL004328 | GC | MAPK1 |
| NA | MOL004328 | GC | TLR2 |
| NA | MOL004328 | GC | AHR |
| NA | MOL004328 | GC | BCL2 |
| NA | MOL004328 | GC | BCL2L1 |
| NA | MOL004328 | GC | CDKN2A |
| Q | MOL000098 | GC | COL3A1 |
| Q | MOL000098 | GC | CRP |
| Q | MOL000098 | GC | CTSD |
| Q | MOL000098 | GC | CYP1A1 |
| Q | MOL000098 | GC | EGFR |
| Q | MOL000098 | GC | ERBB2 |
| Q | MOL000098 | GC | FOS |
| Q | MOL000098 | GC | HIF1A |
| Q | MOL000098 | GC | ICAM1 |
| Q | MOL000098 | GC | IGF2 |
| Q | MOL000098 | GC | IL6 |
| Q | MOL000098 | GC | IRF1 |
| Q | MOL000098 | GC | MMP1 |
| Q | MOL000098 | GC | MYC |
| Q | MOL000098 | GC | ALB |
| Q | MOL000098 | GC | NOS4 |
| Q | MOL000098 | GC | NOS10 |
| Q | MOL000098 | GC | VEGFA |
| Q | MOL000098 | GC | PCNA |
| Q | MOL000098 | GC | PGR |
| Q | MOL000098 | GC | PON1 |
| Q | MOL000098 | GC | POR |
| Q | MOL000098 | GC | PPARG |
| Q | MOL000098 | GC | VCAM1 |
| Q | MOL000098 | GC | CASP3 |
| Q | MOL000098 | GC | MMP2 |
| Q | MOL000098 | GC | RAF1 |
| Q | MOL000098 | GC | AHR |
| Q | MOL000098 | GC | BCL2 |
| Q | MOL000098 | GC | CASP9 |
| SI | MOL000359 | GC | CASP9 |
|  | MOL000422 | GC | CASP9 |
|  | MOL000098 | GC | CASP9 |
|  | MOL004328 | GC | CASP9 |
|  | MOL000422 | GC | CD36 |
|  | MOL000098 | GC | CDC25C |
|  | MOL004328 | GC | CDKN2A |
|  | MOL000497 | GC | CDKN2A |
|  | MOL000098 | GC | CDKN2A |
|  | MOL000422 | GC | CDKN2A |
|  | MOL000098 | GC | CHRM3 |
|  | MOL001484 | GC | CHRM3 |
|  | MOL002565 | GC | CHRM3 |
|  | MOL003896 | GC | CHRM3 |
|  | MOL004891 | GC | CHRM3 |
|  | MOL005003 | GC | CHRM3 |
|  | MOL000422 | GC | CHRM3 |
|  | MOL004328 | GC | CHRM3 |
|  | MOL001792 | GC | CHRM3 |
|  | MOL002311 | GC | CHRM3 |
|  | MOL003656 | GC | CHRM3 |
|  | MOL000392 | GC | CHRM3 |
|  | MOL000417 | GC | CHRM3 |
|  | MOL004806 | GC | COL3A1 |
|  | MOL004808 | GC | COL3A1 |
|  | MOL004810 | GC | COL3A1 |
|  | MOL004811 | GC | COL3A1 |
|  | MOL004814 | GC | COL3A1 |
|  | MOL004820 | GC | COL3A1 |
|  | MOL004827 | GC | CRP |
|  | MOL004828 | GC | CRP |
|  | MOL004829 | GC | CRP |
|  | MOL004833 | GC | CRP |
|  | MOL004835 | GC | CRP |
|  | MOL004841 | GC | CRP |
|  | MOL004848 | GC | CTSD |
|  | MOL004855 | GC | CTSD |
|  | MOL004856 | GC | CTSD |
|  | MOL004857 | GC | CTSD |
|  | MOL004879 | GC | CTSD |
|  | MOL004882 | GC | CTSD |
|  | MOL004883 | GC | CYCS |
|  | MOL004884 | GC | CYP1A1 |
|  | MOL004885 | GC | CYP1A1 |
|  | MOL004891 | GC | CYP1A1 |
|  | MOL004904 | GC | CYP1A1 |
|  | MOL004907 | GC | ERBB2 |
|  | MOL004908 | GC | ESR1 |
|  | MOL004910 | GC | ESR1 |
|  | MOL004911 | GC | ESR1 |
|  | MOL004912 | GC | ESR1 |
|  | MOL004915 | GC | ESR1 |
|  | MOL004935 | GC | ESR1 |
|  | MOL004941 | GC | ESR1 |
|  | MOL004945 | GC | ESR1 |
|  | MOL004948 | GC | ESR1 |
|  | MOL004949 | GC | ESR1 |
|  | MOL004957 | GC | GBA |
|  | MOL004959 | GC | GBA |
|  | MOL004961 | GC | GBA |
|  | MOL004966 | GC | GBA |
|  | MOL000497 | GC | GBA |
|  | MOL004974 | GC | MMP3 |
|  | MOL004980 | GC | MMP3 |
|  | MOL004988 | GC | MMP9 |
|  | MOL004989 | GC | MMP9 |
|  | MOL004991 | GC | HIF1A |
|  | MOL004993 | GC | HIF1A |
|  | MOL000500 | GC | HIF1A |
|  | MOL005000 | GC | HIF1A |
|  | MOL005001 | GC | HIF1A |
|  | MOL005003 | GC | MAPK1 |
|  | MOL005007 | GC | MAPK1 |
|  | MOL005008 | GC | MAPK1 |
|  | MOL005012 | GC | MCL1 |
|  | MOL005016 | GC | MCL1 |
|  | MOL005017 | GC | MCL1 |
|  | MOL005018 | GC | MCL1 |
|  | MOL005020 | GC | MCL1 |
|  | MOL000354 | GC | MMP1 |
|  | MOL000098 | GC | MMP1 |
|  | MOL000098 | GC | MMP2 |
|  | MOL000422 | GC | MMP2 |
|  | MOL000098 | GC | MUC1 |
|  | MOL000098 | GC | MYC |
|  | MOL004328 | GC | MYC |
|  | MOL002565 | GC | MYC |
|  | MOL000422 | GC | MYC |
|  | MOL003896 | GC | MMP9 |
|  | MOL004820 | GC | MMP9 |
|  | MOL004829 | GC | MMP9 |
|  | MOL004833 | GC | MMP9 |
|  | MOL004835 | GC | MMP9 |
|  | MOL004885 | GC | MMP3 |
|  | MOL004891 | GC | MMP3 |
|  | MOL004908 | GC | MMP3 |
|  | MOL004959 | GC | MMP3 |
|  | MOL004966 | GC | MMP3 |
|  | MOL004993 | GC | MUC1 |
|  | MOL005007 | GC | MUC1 |
|  | MOL000354 | GC | MUC1 |
|  | MOL000422 | GC | IL6 |
|  | MOL000098 | GC | IL6 |
|  | MOL000359 | GC | PCNA |
|  | MOL000239 | GC | NOS3 |
|  | MOL003656 | GC | NOS3 |
|  | MOL003896 | GC | NOS3 |
|  | MOL000417 | GC | IL6 |
|  | MOL004808 | GC | EGFR |
|  | MOL004811 | GC | EGFR |
|  | MOL004820 | GC | EGFR |
|  | MOL004848 | GC | ALB |
|  | MOL004855 | GC | ALB |
|  | MOL004856 | GC | ALB |
|  | MOL004857 | GC | ALB |
|  | MOL004879 | GC | NOS3 |
|  | MOL004883 | GC | NOS3 |
|  | MOL004908 | GC | NOS3 |
|  | MOL004911 | GC | NOS9 |
|  | MOL004949 | GC | NOS11 |
|  | MOL004959 | GC | NOS13 |
|  | MOL004961 | GC | NOS15 |
|  | MOL004966 | GC | ESR1 |
|  | MOL000497 | GC | MMP2 |
|  | MOL004974 | GC | MAPK1 |
|  | MOL004980 | GC | ESR1 |
|  | MOL004985 | GC | MMP2 |
|  | MOL004988 | GC | IL6 |
|  | MOL004991 | GC | MYC |
|  | MOL004996 | GC | ESR1 |
|  | MOL005000 | GC | EGFR |
|  | MOL005001 | GC | VEGFA |
|  | MOL005003 | GC | ESR1 |
|  | MOL005007 | GC | MMP9 |
|  | MOL005016 | GC | FOS |
|  | MOL005018 | GC | IL6 |
|  | MOL005020 | GC | VEGFA |
|  | MOL000354 | GC | MYC |
|  | MOL000098 | GC | MMP9 |
|  | MOL000098 | GC | MAPK1 |
|  | MOL000098 | GC | FOS |
|  | MOL000422 | GC | VEGFA |
|  | MOL000359 | GC | ESR1 |
|  | MOL000354 | GC | ERBB2 |
|  | MOL000098 | GC | PPARG |
|  | MOL000422 | GC | ICAM1 |
|  | MOL000211 | GC | PGR |
|  | MOL000098 | GC | PGR |
|  | MOL004328 | GC | PGR |
|  | MOL000098 | GC | EGFR |
|  | MOL000422 | GC | EGFR |
|  | MOL000422 | GC | PON1 |
|  | MOL000098 | GC | PON1 |
|  | MOL004328 | GC | PON1 |
|  | MOL002311 | GC | PON1 |
|  | MOL003656 | GC | PON1 |
|  | MOL003896 | GC | PON1 |
|  | MOL000392 | GC | PON1 |
|  | MOL000417 | GC | PON1 |
|  | MOL004808 | GC | PON1 |
|  | MOL004810 | GC | PON1 |
|  | MOL004811 | GC | PON1 |
|  | MOL004820 | GC | POR |
|  | MOL004827 | GC | POR |
|  | MOL004828 | GC | POR |
|  | MOL004833 | GC | POR |
|  | MOL004835 | GC | POR |
|  | MOL004841 | GC | POR |
|  | MOL004848 | GC | PPARG |
|  | MOL004855 | GC | PPARG |
|  | MOL004856 | GC | PPARG |
|  | MOL004857 | GC | PPARG |
|  | MOL004879 | GC | PPARG |
|  | MOL004883 | GC | PPARG |
|  | MOL004884 | GC | PPARG |
|  | MOL004885 | GC | PPARG |
|  | MOL004891 | GC | PPARG |
|  | MOL004904 | GC | PPARG |
|  | MOL004907 | GC | PPARG |
|  | MOL004908 | GC | PPARG |
|  | MOL004911 | GC | PPARG |
|  | MOL004912 | GC | PPARG |
|  | MOL004915 | GC | PPARG |
|  | MOL004949 | GC | PPARG |
|  | MOL004957 | GC | PPARG |
|  | MOL004959 | GC | PPARG |
|  | MOL004961 | GC | PPARG |
|  | MOL004966 | GC | PPARG |
|  | MOL000497 | GC | PPARG |
|  | MOL004974 | GC | PPARG |
|  | MOL004980 | GC | PPARG |
|  | MOL004991 | GC | PPARG |
|  | MOL000500 | GC | PPARG |
|  | MOL005000 | GC | PPARG |
|  | MOL005003 | GC | PPARG |
|  | MOL005007 | GC | PPARG |
|  | MOL005012 | GC | PPARG |
|  | MOL005016 | GC | PPARG |
|  | MOL005017 | GC | PPARG |
|  | MOL005020 | GC | PRSS1 |
|  | MOL000354 | GC | PRSS1 |
|  | MOL000098 | GC | PRSS1 |
|  | MOL000422 | GC | PRSS1 |
|  | MOL001484 | GC | PRSS1 |
|  | MOL000239 | GC | PRSS1 |
|  | MOL002565 | GC | PRSS1 |
|  | MOL003656 | GC | PRSS1 |
|  | MOL003896 | GC | PRSS1 |
|  | MOL000392 | GC | PRSS1 |
|  | MOL000417 | GC | PRSS1 |
|  | MOL004808 | GC | PRSS1 |
|  | MOL004810 | GC | PRSS1 |
|  | MOL004811 | GC | PRSS1 |
|  | MOL004820 | GC | PRSS1 |
|  | MOL004827 | GC | PRSS1 |
|  | MOL004828 | GC | PRSS1 |
|  | MOL004833 | GC | PRSS1 |
|  | MOL004855 | GC | PRSS1 |
|  | MOL004856 | GC | PRSS1 |
|  | MOL004857 | GC | PRSS1 |
|  | MOL004879 | GC | PRSS1 |
|  | MOL004883 | GC | PRSS1 |
|  | MOL004884 | GC | RAF1 |
| C | MOL003044 | GLR | ESR1 |
| C | MOL003044 | GLR | MAPK1 |
| C | MOL003044 | GLR | MYC |
| C | MOL003044 | GLR | PPARG |
| C | MOL003044 | GLR | PRSS1 |
| C | MOL003044 | GLR | RAF1 |
| ST | MOL000449 | GLR | PRSS1 |
| ST | MOL000449 | GLR | RAF1 |
| ST | MOL000449 | GLR | RASA1 |
| ST | MOL000449 | GLR | BCL2 |
| ST | MOL000449 | GLR | HIF1A |
| ST | MOL000449 | GLR | MUC1 |
| ST | MOL000449 | GLR | NOS3 |
|  | MOL000449 | GLR | EGFR |
|  | MOL000449 | GLR | PRSS1 |
|  | MOL000449 | GLR |  |
| B | MOL000358 | HQ | BCL2 |
| B | MOL000358 | HQ | CASP3 |
| B | MOL000358 | HQ | CASP9 |
| B | MOL000358 | HQ | CHRM3 |
| B | MOL000358 | HQ | MMP9 |
| B | MOL000358 | HQ | NOS3 |
| B | MOL000358 | HQ | PGR |
| B | MOL000358 | HQ | PON1 |
| B | MOL000358 | HQ | PPARG |
| B | MOL000358 | HQ | IL6 |
| C | MOL000073 | HQ | ESR1 |
| C | MOL000073 | HQ | ALB |
| C | MOL002914 | HQ | NOS3 |
| C | MOL002914 | HQ | IL6 |
| SI | MOL000359 | HQ | PPARG |
| SI | MOL000359 | HQ | PRSS1 |
| SI | MOL000359 | HQ | RAF1 |
| ST | MOL000449 | HQ | ESR1 |
| ST | MOL000449 | HQ | RASA1 |
| ST | MOL000449 | HQ | TLR2 |
|  | MOL002714 | HQ | GBA |
|  | MOL000358 | HQ | GBA |
|  | MOL000173 | HQ | MMP3 |
|  | MOL001689 | HQ | MMP9 |
|  | MOL002714 | HQ | MMP9 |
|  | MOL002928 | HQ | APOB |
|  | MOL000358 | HQ | APOB |
|  | MOL000173 | HQ | APOB |
|  | MOL001689 | HQ | BCL2 |
|  | MOL002714 | HQ | BCL2 |
|  | MOL002928 | HQ | CASP3 |
|  | MOL000358 | HQ | CASP3 |
|  | MOL000173 | HQ | CASP3 |
|  | MOL000449 | HQ | CASP3 |
|  | MOL000228 | HQ | CASP9 |
|  | MOL002879 | HQ | CASP9 |
|  | MOL002714 | HQ | CYP1A1 |
|  | MOL002928 | HQ | ERBB2 |
|  | MOL000173 | HQ | ESR1 |
|  | MOL000228 | HQ | ESR1 |
|  | MOL002934 | HQ | ESR1 |
|  | MOL000073 | HQ | ESR1 |
|  | MOL001458 | HQ | ESR1 |
|  | MOL002897 | HQ | ESR1 |
|  | MOL002714 | HQ | ESR1 |
|  | MOL000173 | HQ | FOS |
|  | MOL002928 | HQ | FOS |
|  | MOL000449 | HQ | FOS |
|  | MOL001689 | HQ | IL6 |
|  | MOL002714 | HQ | IL6 |
|  | MOL002927 | HQ | IL6 |
|  | MOL002928 | HQ | IL6 |
|  | MOL002932 | HQ | IL6 |
|  | MOL002937 | HQ | IL6 |
|  | MOL008206 | HQ | IL6 |
|  | MOL012266 | HQ | IL6 |
|  | MOL000358 | HQ | IL6 |
|  | MOL000449 | HQ | IL6 |
|  | MOL002914 | HQ | IL6 |
|  | MOL000359 | HQ | IL6 |
|  | MOL001689 | HQ | NOS3 |
|  | MOL002714 | HQ | NOS3 |
|  | MOL002915 | HQ | ALB |
|  | MOL002927 | HQ | ALB |
|  | MOL002928 | HQ | ALB |
|  | MOL002934 | HQ | EGFR |
|  | MOL002897 | HQ | EGFR |
|  | MOL012266 | HQ | CASP3 |
|  | MOL002714 | HQ | CASP3 |
|  | MOL000359 | HQ | MYC |
|  | MOL000358 | HQ | PCNA |
|  | MOL000449 | HQ | PGR |
|  | MOL000359 | HQ | PGR |
|  | MOL000358 | HQ | PGR |
|  | MOL000173 | HQ | PGR |
|  | MOL002934 | HQ | PGR |
|  | MOL000525 | HQ | PGR |
|  | MOL008206 | HQ | PGR |
|  | MOL001689 | HQ | PGR |
|  | MOL002714 | HQ | PGR |
|  | MOL002915 | HQ | PPARG |
|  | MOL002927 | HQ | PPARG |
|  | MOL002928 | HQ | PPARG |
|  | MOL002932 | HQ | PPARG |
|  | MOL002934 | HQ | PPARG |
|  | MOL001458 | HQ | PPARG |
|  | MOL002897 | HQ | PPARG |
|  | MOL008206 | HQ | PPARG |
|  | MOL012266 | HQ | PPARG |
|  | MOL000173 | HQ | PPARG |
|  | MOL000358 | HQ | PPARG |
|  | MOL000449 | HQ | PPARG |
|  | MOL002914 | HQ | PPARG |
|  | MOL001689 | HQ | PPARG |
|  | MOL000228 | HQ | PPARG |
|  | MOL002714 | HQ | PRSS1 |
|  | MOL002910 | HQ | PRSS1 |
|  | MOL002913 | HQ | PRSS1 |
|  | MOL002915 | HQ | PRSS1 |
|  | MOL002927 | HQ | PRSS1 |
|  | MOL002928 | HQ | PRSS1 |
|  | MOL002932 | HQ | RAF1 |
| B | MOL000358 | JYH | BCL2 |
| B | MOL000358 | JYH | CASP3 |
| B | MOL000358 | JYH | CASP9 |
| B | MOL000358 | JYH | CHRM3 |
| B | MOL000358 | JYH | ALB |
| B | MOL000358 | JYH | PGR |
| B | MOL000358 | JYH | PON1 |
| B | MOL000358 | JYH | PPARG |
| B | MOL000358 | JYH | EGFR |
| BE | MOL002773 | JYH | ALB |
| BE | MOL002773 | JYH | BCL2 |
| BE | MOL002773 | JYH | CASP3 |
| BE | MOL002773 | JYH | CASP9 |
| BE | MOL002773 | JYH | MYC |
| BE | MOL002773 | JYH | VEGFA |
| C | MOL002914 | JYH | ALB |
| C | MOL002914 | JYH | ESR1 |
| K | MOL000422 | JYH | AHR |
| K | MOL000422 | JYH | BCL2 |
| K | MOL000422 | JYH | CASP3 |
| K | MOL000422 | JYH | CYP1A1 |
| K | MOL000422 | JYH | ICAM1 |
| K | MOL000422 | JYH | MAPK1 |
| K | MOL000422 | JYH | PCNA |
| K | MOL000422 | JYH | MMP2 |
| K | MOL000422 | JYH | PGR |
| K | MOL000422 | JYH | PPARG |
| K | MOL000422 | JYH | PRSS1 |
| K | MOL000422 | JYH | ESR1 |
| K | MOL000422 | JYH | RB1 |
| K | MOL000422 | JYH | SELE |
| LU | MOL000006 | JYH | SELE |
| LU | MOL000006 | JYH | TOP1 |
| LU | MOL000006 | JYH | TLR2 |
| LU | MOL000006 | JYH | VCAM1 |
| LU | MOL000006 | JYH | BCL2L1 |
| LU | MOL000006 | JYH | BCL2 |
| LU | MOL000006 | JYH | ESR1 |
| LU | MOL000006 | JYH | AHR |
| MA | MOL001494 | JYH | CASP3 |
| MA | MOL001494 | JYH | CASP9 |
| Q | MOL000098 | JYH | CDKN2A |
| Q | MOL000098 | JYH | COL3A1 |
| Q | MOL000098 | JYH | CRP |
| Q | MOL000098 | JYH | CTSD |
| Q | MOL000098 | JYH | CYP1A1 |
| Q | MOL000098 | JYH | EGFR |
| Q | MOL000098 | JYH | ERBB2 |
| Q | MOL000098 | JYH | FOS |
| Q | MOL000098 | JYH | HIF1A |
| Q | MOL000098 | JYH | ICAM1 |
| Q | MOL000098 | JYH | IGF2 |
| Q | MOL000098 | JYH | IRF1 |
| Q | MOL000098 | JYH | MMP1 |
| Q | MOL000098 | JYH | MYC |
| Q | MOL000098 | JYH | ALB |
| Q | MOL000098 | JYH | NOS3 |
| Q | MOL000098 | JYH | NOS8 |
| Q | MOL000098 | JYH | PCNA |
| Q | MOL000098 | JYH | PGR |
| Q | MOL000098 | JYH | PON1 |
| Q | MOL000098 | JYH | POR |
| Q | MOL000098 | JYH | PPARG |
| Q | MOL000098 | JYH | PRSS1 |
| Q | MOL000098 | JYH | RAF1 |
| Q | MOL000098 | JYH | RASA1 |
| Q | MOL000098 | JYH | TIMP1 |
| Q | MOL000098 | JYH | TP63 |
| Q | MOL000098 | JYH | MMP9 |
| Q | MOL000098 | JYH | MMP3 |
| Q | MOL000098 | JYH | MUC1 |
| Q | MOL000098 | JYH | MAPK1 |
| Q | MOL000098 | JYH | AHR |
| Q | MOL000098 | JYH | BCL2 |
| Q | MOL000098 | JYH | BCL2L1 |
| Q | MOL000098 | JYH | CASP3 |
| Q | MOL000098 | JYH | CASP9 |
| ST | MOL000449 | JYH | CASP9 |
| ST | MOL000449 | JYH | CYP1A1 |
| ST | MOL000449 | JYH | ERBB2 |
| ST | MOL000449 | JYH | ESR1 |
|  | MOL000422 | JYH | ESR1 |
|  | MOL000098 | JYH | ESR1 |
|  | MOL000358 | JYH | ESR1 |
|  | MOL000006 | JYH | ESR1 |
|  | MOL000358 | JYH | FOS |
|  | MOL000422 | JYH | FOS |
|  | MOL000006 | JYH | FOS |
|  | MOL000098 | JYH | IL6 |
|  | MOL000358 | JYH | IRF1 |
|  | MOL000006 | JYH | IRF1 |
|  | MOL000098 | JYH | IRF1 |
|  | MOL000449 | JYH | IRF1 |
|  | MOL000098 | JYH | LDLR |
|  | MOL000098 | JYH | MUC1 |
|  | MOL000422 | JYH | MUC1 |
|  | MOL000098 | JYH | MAPK1 |
|  | MOL000006 | JYH | MAPK1 |
|  | MOL000098 | JYH | NOS3 |
|  | MOL000006 | JYH | NOS3 |
|  | MOL003014 | JYH | NOS3 |
|  | MOL003044 | JYH | ALB |
|  | MOL003111 | JYH | IL6 |
|  | MOL000098 | JYH | EGFR |
|  | MOL000422 | JYH | EGFR |
|  | MOL000006 | JYH | EGFR |
|  | MOL000098 | JYH | MYC |
|  | MOL000006 | JYH | PCNA |
|  | MOL000098 | JYH | PGR |
|  | MOL000422 | JYH | PGR |
|  | MOL000006 | JYH | PGR |
|  | MOL000098 | JYH | PPARG |
|  | MOL003044 | JYH | PPARG |
|  | MOL003111 | JYH | PPARG |
|  | MOL000449 | JYH | PPARG |
|  | MOL001494 | JYH | PPARG |
|  | MOL001495 | JYH | PPARG |
|  | MOL003036 | JYH | PPARG |
|  | MOL003111 | JYH | PRSS1 |
|  | MOL000358 | JYH | PRSS1 |
|  | MOL000422 | JYH | PRSS1 |
|  | MOL000006 | JYH | PRSS1 |
|  | MOL000098 | JYH | PRSS1 |
|  | MOL000449 | JYH | PRSS1 |
|  | MOL002914 | JYH | PRSS1 |
|  | MOL000006 | JYH | RAF1 |
|  | MOL000098 | JYH | RAF1 |
| B | MOL000358 | LQ | MUC1 |
| B | MOL000358 | LQ | BCL2 |
| B | MOL000358 | LQ | CASP3 |
| B | MOL000358 | LQ | CASP9 |
| B | MOL000358 | LQ | CHRM3 |
| B | MOL000358 | LQ | IL6 |
| B | MOL000358 | LQ | PGR |
| B | MOL000358 | LQ | PON1 |
| K | MOL000422 | LQ | PPARG |
| K | MOL000422 | LQ | EGFR |
| K | MOL000422 | LQ | AHR |
| K | MOL000422 | LQ | BCL2 |
| K | MOL000422 | LQ | CASP3 |
| K | MOL000422 | LQ | CYP1A1 |
| K | MOL000422 | LQ | ICAM1 |
| K | MOL000422 | LQ | MAPK1 |
| K | MOL000422 | LQ | NOS3 |
| K | MOL000422 | LQ | PGR |
| K | MOL000422 | LQ | PRSS1 |
| K | MOL000422 | LQ | CASP9 |
| K | MOL000422 | LQ | TLR2 |
| Q | MOL000098 | LQ | BCL2 |
| Q | MOL000098 | LQ | BCL2L1 |
| Q | MOL000098 | LQ | CASP3 |
| Q | MOL000098 | LQ | CASP9 |
| Q | MOL000098 | LQ | CDKN2A |
| Q | MOL000098 | LQ | COL3A1 |
| Q | MOL000098 | LQ | CRP |
| Q | MOL000098 | LQ | CTSD |
| Q | MOL000098 | LQ | CYP1A1 |
| Q | MOL000098 | LQ | EGFR |
| Q | MOL000098 | LQ | ERBB2 |
| Q | MOL000098 | LQ | FOS |
| Q | MOL000098 | LQ | HIF1A |
| Q | MOL000098 | LQ | ICAM1 |
| Q | MOL000098 | LQ | IGF2 |
| Q | MOL000098 | LQ | IL6 |
| Q | MOL000098 | LQ | IRF1 |
| Q | MOL000098 | LQ | MMP1 |
| Q | MOL000098 | LQ | MYC |
| Q | MOL000098 | LQ | ALB |
| Q | MOL000098 | LQ | NOS3 |
| Q | MOL000098 | LQ | VEGFA |
| Q | MOL000098 | LQ | MMP2 |
| Q | MOL000098 | LQ | PGR |
| Q | MOL000098 | LQ | PON1 |
| Q | MOL000098 | LQ | POR |
| Q | MOL000098 | LQ | PPARG |
| Q | MOL000098 | LQ | TP63 |
| Q | MOL000098 | LQ | VCAM1 |
| Q | MOL000098 | LQ | PCNA |
| Q | MOL000098 | LQ | AHR |
|  | MOL000422 | LQ | CASP3 |
|  | MOL000098 | LQ | CASP3 |
|  | MOL000358 | LQ | CASP3 |
|  | MOL000422 | LQ | CASP9 |
|  | MOL000098 | LQ | CYP1A1 |
|  | MOL000173 | LQ | CYP1A1 |
|  | MOL000006 | LQ | ERBB2 |
|  | MOL000098 | LQ | ESR1 |
|  | MOL000358 | LQ | ESR1 |
|  | MOL000422 | LQ | ESR1 |
|  | MOL000006 | LQ | ESR1 |
|  | MOL000173 | LQ | ESR1 |
|  | MOL003330 | LQ | ESR1 |
|  | MOL000098 | LQ | FOS |
|  | MOL000422 | LQ | GBA |
|  | MOL000098 | LQ | GBA |
|  | MOL000006 | LQ | GBA |
|  | MOL000098 | LQ | LDLR |
|  | MOL000006 | LQ | MAPK1 |
|  | MOL000098 | LQ | MAPK1 |
|  | MOL000173 | LQ | MAPK1 |
|  | MOL000791 | LQ | MAPK1 |
|  | MOL000422 | LQ | MUC1 |
|  | MOL000006 | LQ | MUC1 |
|  | MOL003347 | LQ | NOS3 |
|  | MOL000098 | LQ | NOS3 |
|  | MOL000098 | LQ | IL6 |
|  | MOL000006 | LQ | IL6 |
|  | MOL000098 | LQ | EGFR |
|  | MOL000173 | LQ | EGFR |
|  | MOL000422 | LQ | ALB |
|  | MOL000006 | LQ | PCNA |
|  | MOL000173 | LQ | PCNA |
|  | MOL000006 | LQ | PGR |
|  | MOL000522 | LQ | PGR |
|  | MOL000098 | LQ | PGR |
|  | MOL003295 | LQ | PPARG |
|  | MOL003306 | LQ | PPARG |
|  | MOL003308 | LQ | PPARG |
|  | MOL003322 | LQ | PPARG |
|  | MOL000522 | LQ | PPARG |
|  | MOL000358 | LQ | PPARG |
|  | MOL000422 | LQ | PRSS1 |
|  | MOL000006 | LQ | PRSS1 |
|  | MOL000098 | LQ | PRSS1 |
|  | MOL003295 | LQ | PRSS1 |
|  | MOL003306 | LQ | PRSS1 |
|  | MOL003308 | LQ | PRSS1 |
|  | MOL003322 | LQ | PRSS1 |
|  | MOL003330 | LQ | PRSS1 |
|  | MOL000098 | LQ | RAF1 |
|  | MOL000422 | LQ | RAF1 |
|  | MOL000098 | LQ | VEGFA |
|  | MOL000522 | NBZ | ESR1 |
| K | MOL000422 | NBZ | RAF1 |
| B | MOL000358 | NBZ | BCL2 |
| B | MOL000358 | NBZ | CASP3 |
| B | MOL000358 | NBZ | CASP9 |
| B | MOL000358 | NBZ | PON1 |
| B | MOL000358 | NBZ | PPARG |
| K | MOL000422 | NBZ | RB1 |
| K | MOL000422 | NBZ | AHR |
| K | MOL000422 | NBZ | BCL2 |
| K | MOL000422 | NBZ | CASP3 |
| K | MOL000422 | NBZ | GBA |
| K | MOL000422 | NBZ | ICAM1 |
| K | MOL000422 | NBZ | ALB |
| K | MOL000422 | NBZ | VEGFA |
| K | MOL000422 | NBZ | PGR |
| K | MOL000422 | NBZ | PPARG |
| K | MOL000422 | NBZ | PRSS1 |
| K | MOL000422 | NBZ | CASP9 |
| BE | MOL002773 | NBZ | MYC |
| BE | MOL002773 | NBZ | VEGFA |
| BE | MOL002773 | NBZ | EGFR |
| BE | MOL002773 | NBZ | ALB |
| BE | MOL002773 | NBZ | AHR |
| BE | MOL002773 | NBZ | HIF1A |
|  | MOL000358 | NBZ | NOS3 |
|  | MOL000358 | NBZ | CDKN2A |
|  | MOL000358 | NBZ | PPARG |
|  | MOL000358 | NBZ | ESR1 |
|  | MOL000358 | NBZ | BCL2 |
|  | MOL000358 | NBZ | ERBB2 |
|  | MOL000358 | NBZ | IL6 |
|  | MOL000422 | NBZ | EGFR |
|  | MOL000422 | NBZ | PCNA |
|  | MOL000422 | NBZ | PPARG |
|  | MOL000422 | NBZ | PRSS1 |
|  | MOL000422 | NBZ | CASP3 |
|  | MOL000422 | NBZ | ESR1 |
|  | MOL000422 | NBZ | RAF1 |
|  | MOL000422 | NBZ | MMP9 |
|  | MOL000422 | NBZ | PLG |
|  | MOL000422 | NBZ | AHR |
|  | MOL000422 | NBZ | ICAM1 |
|  | MOL000422 | NBZ | NOS3 |
|  | MOL000422 | NBZ | RB1 |
|  | MOL002773 | NBZ | CXCL8 |
|  | MOL002773 | NBZ | BCL2 |
|  | MOL002773 | NBZ | ERBB2 |
|  | MOL002773 | NBZ | ESR1 |
|  | MOL002773 | NBZ | ICAM1 |
|  | MOL002773 | NBZ | NOS3 |
|  | MOL000358 | NBZ | EGFR |
|  | MOL000358 | NBZ | PCNA |
|  | MOL000358 | NBZ | PRSS1 |
| NA | MOL004328 | QP | CASP3 |
| NA | MOL004328 | QP | ERBB2 |
| NA | MOL004328 | QP | MCL1 |
| NA | MOL004328 | QP | ALB |
| NA | MOL004328 | QP | PPARG |
| NA | MOL004328 | QP | RB1 |
| NA | MOL004328 | QP | VCAM1 |
| NA | MOL004328 | QP | PGR |
| NA | MOL004328 | QP | APOB |
| NA | MOL004328 | QP | BCL2 |
| NO | MOL005828 | QP | ESR1 |
| NO | MOL005828 | QP | LDLR |
| NO | MOL005828 | QP | MMP2 |
| NO | MOL005828 | QP | EGFR |
| NO | MOL005828 | QP | PON1 |
| NO | MOL005828 | QP | PPARG |
| NO | MOL005828 | QP | VEGFA |
| NO | MOL005828 | QP | RB1 |
| NO | MOL005828 | QP | ALB |
| NO | MOL005828 | QP | SELE |
|  | MOL004328 | QP | SELE |
|  | MOL004328 | QP | CHRM3 |
|  | MOL005828 | QP | PGR |
|  | MOL004328 | QP | CASP3 |
|  | MOL005828 | QP | CASP3 |
|  | MOL004328 | QP | ESR1 |
|  | MOL005828 | QP | ESR1 |
|  | MOL004328 | QP | IGF2 |
|  | MOL001803 | QP | IGF2 |
|  | MOL001803 | QP | EGFR |
|  | MOL004328 | QP | PGR |
|  | MOL005828 | QP | PPARG |
|  | MOL001803 | QP | PPARG |
|  | MOL005828 | QP | PRSS1 |
|  | MOL001798 | QP | PRSS1 |
|  | MOL001803 | QP | PRSS1 |
|  | MOL004328 | QP | PRSS1 |
| LU | MOL000006 | THF | ICAM1 |
| Q | MOL000098 | THF | PCNA |
| Q | MOL000098 | THF | NOS3 |
| B | MOL000358 | ZJC | BCL2 |
| B | MOL000358 | ZJC | CASP3 |
| B | MOL000358 | ZJC | CASP9 |
| B | MOL000358 | ZJC | CHRM3 |
| B | MOL000358 | ZJC | GBA |
| B | MOL000358 | ZJC | NOS3 |
| B | MOL000358 | ZJC | PGR |
| B | MOL000358 | ZJC | PON1 |
| B | MOL000358 | ZJC | PPARG |
| B | MOL000358 | ZJC | VEGFA |
| C | MOL000073 | ZJC | ESR1 |
| C | MOL000073 | ZJC | VEGFA |
| C | MOL002914 | ZJC | NOS3 |
| C | MOL002914 | ZJC | VEGFA |
| K | MOL000422 | ZJC | AHR |
| K | MOL000422 | ZJC | BCL2 |
| K | MOL000422 | ZJC | CASP3 |
| K | MOL000422 | ZJC | CYP1A1 |
| K | MOL000422 | ZJC | ICAM1 |
| K | MOL000422 | ZJC | MAPK1 |
| K | MOL000422 | ZJC | EGFR |
| K | MOL000422 | ZJC | PGR |
| K | MOL000422 | ZJC | PPARG |
| K | MOL000422 | ZJC | PRSS1 |
| K | MOL000422 | ZJC | RB1 |
| K | MOL000422 | ZJC | TEP1 |
| K | MOL000422 | ZJC | EPHB2 |
| K | MOL000422 | ZJC | TP63 |
| Q | MOL000098 | ZJC | BCL2 |
| Q | MOL000098 | ZJC | BCL2L1 |
| Q | MOL000098 | ZJC | CASP3 |
| Q | MOL000098 | ZJC | CASP9 |
| Q | MOL000098 | ZJC | CDKN2A |
| Q | MOL000098 | ZJC | COL3A1 |
| Q | MOL000098 | ZJC | CRP |
| Q | MOL000098 | ZJC | CTSD |
| Q | MOL000098 | ZJC | CYP1A1 |
| Q | MOL000098 | ZJC | EGFR |
| Q | MOL000098 | ZJC | ERBB2 |
| Q | MOL000098 | ZJC | FOS |
| Q | MOL000098 | ZJC | MMP3 |
| Q | MOL000098 | ZJC | HIF1A |
| Q | MOL000098 | ZJC | ICAM1 |
| Q | MOL000098 | ZJC | IGF2 |
| Q | MOL000098 | ZJC | IL6 |
| Q | MOL000098 | ZJC | IRF1 |
| Q | MOL000098 | ZJC | MMP1 |
| Q | MOL000098 | ZJC | MYC |
| Q | MOL000098 | ZJC | PCNA |
| Q | MOL000098 | ZJC | ALB |
| Q | MOL000098 | ZJC | NOS6 |
| Q | MOL000098 | ZJC | NOS14 |
| Q | MOL000098 | ZJC | MMP9 |
| Q | MOL000098 | ZJC | MAPK1 |
| Q | MOL000098 | ZJC | PON1 |
| Q | MOL000098 | ZJC | POR |
| Q | MOL000098 | ZJC | PRSS1 |
| Q | MOL000098 | ZJC | RAF1 |
| Q | MOL000098 | ZJC | VEGFA |
| Q | MOL000098 | ZJC | RB1 |
| Q | MOL000098 | ZJC | SELE |
| Q | MOL000098 | ZJC | PGR |
| Q | MOL000098 | ZJC | CHRM3 |
| Q | MOL000098 | ZJC | AHR |
| SI | MOL000359 | ZJC | BCL2 |
| SI | MOL000359 | ZJC | BCL2L1 |
| ST | MOL000449 | ZJC | CASP3 |
| ST | MOL000449 | ZJC | CYP1A1 |
| ST | MOL000449 | ZJC | EGFR |
|  | MOL000422 | ZJC | EGFR |
|  | MOL000098 | ZJC | EPHB2 |
|  | MOL000358 | ZJC | ESR1 |
|  | MOL000422 | ZJC | ESR1 |
|  | MOL000098 | ZJC | ESR1 |
|  | MOL013179 | ZJC | ESR1 |
|  | MOL013179 | ZJC | ICAM1 |
|  | MOL000098 | ZJC | ICAM1 |
|  | MOL000358 | ZJC | ICAM1 |
|  | MOL000449 | ZJC | ICAM1 |
|  | MOL000098 | ZJC | IGF2 |
|  | MOL000098 | ZJC | MUC1 |
|  | MOL000098 | ZJC | IL6 |
|  | MOL000422 | ZJC | IL6 |
|  | MOL013179 | ZJC | MAPK1 |
|  | MOL000098 | ZJC | PCNA |
|  | MOL000073 | ZJC | PCNA |
|  | MOL000098 | ZJC | NOS3 |
|  | MOL000358 | ZJC | NOS3 |
|  | MOL000422 | ZJC | NOS3 |
|  | MOL000449 | ZJC | NOS3 |
|  | MOL000098 | ZJC | EGFR |
|  | MOL013179 | ZJC | CASP3 |
|  | MOL000098 | ZJC | CASP3 |
|  | MOL000422 | ZJC | CASP3 |
|  | MOL013179 | ZJC | TLR2 |
|  | MOL000098 | ZJC | PGR |
|  | MOL000449 | ZJC | PGR |
|  | MOL000358 | ZJC | PPARG |
|  | MOL000422 | ZJC | PPARG |
|  | MOL000449 | ZJC | PPARG |
|  | MOL000098 | ZJC | PPARG |
|  | MOL013296 | ZJC | PRSS1 |
|  | MOL002914 | ZJC | PRSS1 |
|  | MOL000359 | ZJC | PRSS1 |
|  | MOL000098 | ZJC | PRSS1 |
|  | MOL013179 | ZJC | PRSS1 |
|  | MOL000422 | ZJC | PRSS1 |
|  | MOL000449 | ZJC | RAF1 |
| B | MOL000358 | ZZ | PGR |
| B | MOL000358 | ZZ | AHR |
| K | MOL000422 | ZZ | BCL2 |
| K | MOL000422 | ZZ | BCL2L1 |
| K | MOL000422 | ZZ | CASP3 |
| K | MOL000422 | ZZ | CYP1A1 |
| K | MOL000422 | ZZ | CYP2C9 |
| K | MOL000422 | ZZ | EGFR |
| K | MOL000422 | ZZ | ERBB2 |
| K | MOL000422 | ZZ | MUC1 |
| K | MOL000422 | ZZ | IL6 |
| Q | MOL000098 | ZZ | IL6 |
| Q | MOL000098 | ZZ | PCNA |
| Q | MOL000098 | ZZ | NOS3 |
| Q | MOL000098 | ZZ | VEGFA |
| Q | MOL000098 | ZZ | ALB |
| Q | MOL000098 | ZZ | ESR1 |
| Q | MOL000098 | ZZ | EGFR |
| Q | MOL000098 | ZZ | CASP3 |
| ST | MOL000449 | ZZ | CASP3 |
|  | MOL000422 | ZZ | CASP3 |
|  | MOL000098 | ZZ | CASP3 |
|  | MOL000358 | ZZ | CASP3 |
|  | MOL000098 | ZZ | MYC |
|  | MOL000358 | ZZ | MYC |
|  | MOL000422 | ZZ | MYC |
|  | MOL000098 | ZZ | RAF1 |
|  | MOL001406 | ZZ | RAF1 |
|  | MOL000358 | ZZ | RAF1 |
|  | MOL000449 | ZZ | RASA1 |
|  | MOL000098 | ZZ | RASA1 |
|  | MOL000422 | ZZ | RASA1 |
|  | MOL000098 | ZZ | RB1 |
|  | MOL004561 | ZZ | RB1 |
|  | MOL000358 | ZZ | ALB |
|  | MOL000422 | ZZ | ALB |
|  | MOL000449 | ZZ | ALB |
|  | MOL000098 | ZZ | ALB |
|  | MOL000422 | ZZ | IL6 |
|  | MOL000098 | ZZ | IL6 |
|  | MOL000098 | ZZ | SELE |
|  | MOL004561 | ZZ | SELE |
|  | MOL000422 | ZZ | SELE |
|  | MOL000449 | ZZ | SELE |
|  | MOL001406 | ZZ | SELE |
|  | MOL000358 | ZZ | SELE |
|  | MOL001494 | ZZ | SELE |
|  | MOL002883 | ZZ | SELE |
|  | MOL000098 | ZZ | TOP1 |
|  | MOL000422 | ZZ | Symbol |
|  | MOL000098 | ZZ | TCF7 |
|  | MOL000449 | ZZ | TIMP1 |
|  | MOL000098 | ZZ | TIMP1 |
|  | MOL000358 | ZZ | TIMP1 |
|  | MOL000422 | ZZ | TLR2 |
|  | MOL000449 | ZZ | TLR2 |
|  | MOL000098 | ZZ | TP63 |
|  | MOL000358 | ZZ | TP63 |
|  | MOL000422 | ZZ | TP63 |
|  | MOL000098 | ZZ | VCAM1 |
|  | MOL000358 | ZZ | VCAM1 |
|  | MOL000422 | ZZ | VCAM1 |
|  | MOL001494 | ZZ | VCAM1 |
|  | MOL007245 | ZZ | VCAM1 |
|  | MOL000449 | ZZ | VCAM1 |
|  | MOL000422 | ZZ | VEGFA |
|  | MOL000098 | ZZ | VEGFA |
|  | MOL001406 | ZZ | VEGFA |
