# Supplementary material for: Study the Mechanism of Gualou Niubang Decoction in Treating Plasma Cell Mastitis Based on Network Pharmacology and Molecular Docking
Source: Biomed Res Int. 2022 Jun 15;2022:5780936. doi: 10.1155/2022/5780936 (PMC9217541; doi:10.1155/2022/5780936)
Supplement: Supplementary Materials — S1: 240 active components of Trichosanthis Niubang decoction (including repeated values). S2: PubChem CID information of 151 active components of Trichosanthes Niubang decoction (excluding duplication). S3: Venn diagram of intersection of drugs and diseases. S4: component-ingredient-disease-target gene network data. S5: G0 enrichment analysis (35 cell compositions). S6: G0 enrichment analysis (242 biological processes). S7: G0 enrichment analysis (59 molecular functions). S8: 200 KEGG pathway enrichment analyses. [file 5780936.f1.zip › TableS 5 GO-CC.docx]

S 5 G0 enrichment analysis (35 cell composition).

| Term | Count | PValue | Genes |
| --- | --- | --- | --- |
| GO:0005615~extracellular space | 22 | 2.42E-10 | CRP, VCAM1, CXCL8, MMP2, PON1, GBA, MMP3, IGF2, PLG, SELE, MMP9, EGFR, ICAM1, VEGFA, COL3A1, MUC1, IL6, ALB, TIMP1, CD36, APOB, CTSD |
| GO:0005576~extracellular region | 18 | 5.08E-06 | CRP, PRSS1, CXCL8, MMP1, MMP2, PON1, MMP3, IGF2, PLG, MMP9, VEGFA, COL3A1, IL6, ALB, TIMP1, APOB, SHBG, CTSD |
| GO:0031093~platelet alpha granule lumen | 5 | 2.60E-05 | ALB, IGF2, PLG, TIMP1, VEGFA |
| GO:0045121~membrane raft | 7 | 4.35E-05 | CASP3, CD36, SELE, CTSD, EGFR, ICAM1, TLR2 |
| GO:0005901~caveola | 4 | 0.001104404 | NOS3, INSR, MAPK1, SELE |
| GO:0005741~mitochondrial outer membrane | 5 | 0.001211039 | BCL2, PGR, RAF1, BCL2L1, MCL1 |
| GO:0005794~Golgi apparatus | 10 | 0.001336448 | VCAM1, ALB, MAPK1, PPARG, CD36, APOB, RAF1, ESR1, LDLR, TLR2 |
| GO:0009986~cell surface | 8 | 0.001444431 | VCAM1, PLG, CD36, LDLR, EGFR, ICAM1, TLR2, VEGFA |
| GO:0005578~proteinaceous extracellular matrix | 6 | 0.001486681 | MMP1, MMP2, MMP3, TIMP1, MMP9, VEGFA |
| GO:0010008~endosome membrane | 5 | 0.002669711 | INSR, ERBB2, APOB, LDLR, EGFR |
| GO:0005581~collagen trimer | 4 | 0.002991478 | COL3A1, MMP1, TIMP1, CD36 |
| GO:0005667~transcription factor complex | 5 | 0.003108777 | TCF7, FOS, AHR, HIF1A, TP63 |
| GO:0005654~nucleoplasm | 18 | 0.003959765 | RB1, PCNA, CDKN2A, TCF7, AHR, FOS, CDC25C, HIF1A, ESR1, MYC, CASP3, IRF1, MAPK1, PGR, PPARG, TOP1, TP63, MCL1 |
| GO:0070062~extracellular exosome | 18 | 0.004381357 | CRP, PRSS1, VCAM1, PCNA, INSR, PON1, GBA, IGF2, PLG, MMP9, ICAM1, MUC1, ALB, MAPK1, TIMP1, APOB, SHBG, CTSD |
| GO:0009897~external side of plasma membrane | 5 | 0.004417326 | IL6, VCAM1, CD36, LDLR, ICAM1 |
| GO:0016020~membrane | 15 | 0.006727629 | VCAM1, INSR, FOS, ESR1, CYP19A1, EGFR, ICAM1, VEGFA, POR, ERBB2, BCL2, CD36, LDLR, MCL1, BCL2L1 |
| GO:0043235~receptor complex | 4 | 0.007353094 | INSR, ERBB2, LDLR, EGFR |
| GO:0005739~mitochondrion | 11 | 0.007689895 | CASP9, POR, CDKN2A, MYC, MMP2, BCL2, CYP1A1, MAPK1, TP63, BCL2L1, MCL1 |
| GO:0043231~intracellular membrane-bounded organelle | 7 | 0.007757858 | CYP2C9, POR, INSR, PON1, CYP1A1, PPARG, APOB |
| GO:0097136~Bcl-2 family protein complex | 2 | 0.009354425 | BCL2L1, MCL1 |
| GO:0072562~blood microparticle | 4 | 0.011981336 | PRSS1, PON1, ALB, PLG |
| GO:0005634~nucleus | 26 | 0.01623027 | RB1, PCNA, TCF7, AHR, HIF1A, EGFR, CASP9, MYC, CASP3, ERBB2, MAPK1, TP63, MCL1, CDKN2A, NOS3, MMP2, FOS, CDC25C, ESR1, IRF1, ALB, BCL2, PPARG, PGR, TOP1, RAF1 |
| GO:0016323~basolateral plasma membrane | 4 | 0.018774908 | CHRM3, ERBB2, LDLR, EGFR |
| GO:0005829~cytosol | 18 | 0.022051201 | CDKN2A, NOS3, FOS, CDC25C, HIF1A, CASP9, MYC, RASA1, CASP3, IRF1, BCL2, MAPK1, PPARG, RAF1, APOB, TP63, MCL1, BCL2L1 |
| GO:0000790~nuclear chromatin | 4 | 0.022522873 | MUC1, IRF1, ESR1, TP63 |
| GO:0045177~apical part of cell | 3 | 0.023051382 | VCAM1, CD36, LDLR |
| GO:0005769~early endosome | 4 | 0.034879671 | VCAM1, MAPK1, APOB, LDLR |
| GO:0043234~protein complex | 5 | 0.039786283 | CDKN2A, MYC, ALB, MAPK1, TP63 |
| GO:0034362~low-density lipoprotein particle | 2 | 0.042924223 | APOB, LDLR |
| GO:0005789~endoplasmic reticulum membrane | 7 | 0.051678505 | CYP2C9, POR, BCL2, CYP1A1, APOB, CYP19A1, EGFR |
| GO:0031143~pseudopodium | 2 | 0.051883978 | MAPK1, RAF1 |
| GO:0016324~apical plasma membrane | 4 | 0.062786429 | MUC1, ERBB2, CD36, EGFR |
| GO:0031012~extracellular matrix | 4 | 0.0653844 | COL3A1, MMP1, MMP2, CTSD |
| GO:0043209~myelin sheath | 3 | 0.081942197 | ERBB2, ALB, BCL2 |
| GO:0090575~RNA polymerase II transcription factor complex | 2 | 0.098295398 | PPARG, HIF1A |
